# Pangenome insights into the diversification and disease specificity of worldwide *Xanthomonas* outbreaks

**DOI:** 10.3389/fmicb.2023.1213261

**Published:** 2023-07-05

**Authors:** Viplav Agarwal, Rachel Stubits, Zain Nassrullah, Marcus M. Dillon

**Affiliations:** ^1^Department of Biology, University of Toronto Mississauga, Mississauga, ON, Canada; ^2^Department of Ecology and Evolutionary Biology, University of Toronto, Toronto, ON, Canada

**Keywords:** *Xanthomonas*, plant pathogens, comparative genomics, recombination, pangenomes, virulence factors, type III secretion systems, effectors

## Abstract

The bacterial genus *Xanthomonas* is responsible for disease outbreaks in several hundred plant species, many of them economically important crops. In the era of next-generation sequencing, thousands of strains from this genus have now been sequenced as part of isolated studies that focus on outbreak characterization, host range, diversity, and virulence factor identification. However, these data have not been synthesized and we lack a comprehensive phylogeny for the genus, with some species designations in public databases still relying on phenotypic similarities and representative sequence typing. The extent of genetic cohesiveness among *Xanthomonas* strains, the distribution of virulence factors across strains, and the impact of evolutionary history on host range across the genus are also poorly understood. In this study, we present a pangenome analysis of 1,910 diverse *Xanthomonas* genomes, highlighting their evolutionary relationships, the distribution of virulence-associated genes across strains, and rates of horizontal gene transfer. We find a number of broadly conserved classes of virulence factors and considerable diversity in the Type 3 Secretion Systems (T3SSs) and Type 3 Secreted Effector (T3SE) repertoires of different *Xanthomonas* species. We also use these data to re-assign incorrectly classified strains to phylogenetically informed species designations and find evidence of both monophyletic host specificity and convergent evolution of phylogenetically distant strains to the same host. Finally, we explore the role of recombination in maintaining genetic cohesion within the *Xanthomonas* genus as a result of both ancestral and recent recombination events. Understanding the evolutionary history of *Xanthomonas* species and the relationship of key virulence factors with host-specificity provides valuable insight into the mechanisms through which *Xanthomonas* species shift between hosts and will enable us to develop more robust resistance strategies against these highly virulent pathogens.

## 1. Introduction

*Xanthomonas* is a genus of globally distributed, Gram-negative bacterial pathogens that can infect and cause disease on more than 400 different plant species, many of them economically important crops. Specifically, different *Xanthomonas* strains can devastate wheat, rice, sugarcane, bean, pepper, tomato, citrus, and banana crops by causing diseases like vascular wilts, cankers, leaf spots, and leaf blights (Leyns et al., 1984; White et al., 2009). More recently, *X. cucurbitae* has been implicated in major losses of pumpkin crops in Illinois (Babadoost and Ravanlou, 2012), *X. vasicola* has led to banana, plantain, and enset crop loss in Africa (Tripathi and Mwangi, 2009; Nakato et al., 2018), *X. campestris* has devastated cabbage crops in China (Chen et al., 2021), and various xanthomonads have led to major tomato crop outbreaks in Florida (Horvath et al., 2012). While *Xanthomonas* has the greatest impact in humid tropical climates, this optimal range is likely to expand as climate change creates conditions more suitable to pathogen success (Velásquez et al., 2018).

The phylogenetics and taxonomy of the *Xanthomonas* genus has a long history and has been revised substantially as more sequencing data have become available. All xanthomonads can be traced back to a deep branch of Gammaproteobacteria, forming a monophyletic clade that includes *Xylella fastidiosa*, termed *Xanthomonadales* (Lima et al., 2008). The *Xanthomonas* lineage is then further divided into two early branching subclades (Clades 1A and 1B), and a late branching clade that contains the majority of classified species (Clade 2) (Koebnik et al., 2021). Early species-level classifications relied on *in vitro* bacteriological assays and phenotypic observations like host specificity to categorize strains (Young et al., 1978). These classifications were later refined by molecular techniques including DNA–DNA hybridization, repetitive element PCR (Rep-PCR), and restriction-fragment length polymorphism (RFLP) (Louws et al., 1994; Vauterin et al., 1995; Rademaker et al., 2000; Simões et al., 2007). Most recently, sequencing of housekeeping genes, multi-locus sequence typing, and whole-genome approaches have enabled more fine-scale classification of *Xanthomonas* species that have led to substantial revisions in *Xanthomonas* taxonomy (Hauben et al., 1997; Young et al., 2008; Parkinson et al., 2009; Rodriguez-R et al., 2012; Timilsina et al., 2020). At least 35 species have been proposed in this genus, and many species have been further subdivided into pathovars based on the plant hosts that they infect. The majority of these species share several unifying qualities, including modest genome sizes (~5 Mb, ~4,500 genes) and relatively high GC-contents (~65% GC), but it remains unclear whether all classified species form monophyletic groups in a genus-wide context. Furthermore, several strains that have been deposited in public databases still lack species designations altogether and are classified simply as *Xanthomonas* sp.

While phylogenetic approaches have been an effective tool for delineating *Xanthomonas* species, horizontal gene transfer (HGT) is also an important evolutionary force for maintaining genetic cohesion between xanthomonads and transferring key virulence genes between lineages. Indeed, frequent recombination events have been observed in many *Xanthomonas* species such as *X. campestris*, *X. citri*, *X. euvesicatoria*, and *X. perforans*, leading to the evolution of highly plastic and diverse genomes (Huang et al., 2015; Timilsina et al., 2015; Jibrin et al., 2018). On the other hand, some lineages appear to recombine less frequently and are more clonal, such as certain strains of *X. perforans* in Florida (Timilsina et al., 2019b). Virulence associated genes are especially interesting in this context because exchange of these genes can result in rapid host range shifts and convergent evolution of distantly related strains to the same host. Type III Secreted Effector (T3SE) genes, which can both enhance virulence via interference with host resistance pathways and diminish virulence via recognition by host resistance factors, may be especially prone to HGT because they are frequently located on pathogenicity islands and associated with mobile elements (Jones and Dangl, 2006; Deb et al., 2022). *XopG*, *xopO*, *xopT*, and *xopAJ* are all recent examples of T3SEs that are suspected to have been horizontally transferred between distantly related *Xanthomonas* species (Hajri et al., 2012; Deb et al., 2022), although the fact that T3SE repertoires remain a relatively good descriptor of *Xanthomonas* subclades suggests that many effectors are also being vertically inherited (Guy et al., 2013).

Regardless of the relative roles of vertical inheritance and horizontal transfer, individual *Xanthomonas* strains have clearly undergone substantial diversification that has led to a broad genus-wide host range, but a high degree of host and tissue specificity at the strain level. For example, strains of *X. oryzae* vary in their ability to infect different accessions of rice (Zhang et al., 2021), and pathovars *X. oryzae* pv. *oryzae* and *X. oryzae* pv. *oryzicola* have become specialized to infect vascular and mesophilic tissues, respectively (Ryan et al., 2011). There have also been a number of recently documented cases of host shifts in the genus, including the existence of closely related *X. euvesicatoria* and *X. citri* strains with distinct host specificities, and the emergence of a novel banana pathogen in *X. vasicola* pv. *musacearum,* which was previously an enset pathogen (Tushemereirwe et al., 2004; Bansal et al., 2022; Harrison et al., 2023). This host and disease specificity in *Xanthomonas* is largely determined by the diversification of virulence factors across strains belonging to this genus (An et al., 2020). In particular, key *Xanthomonas* virulence factors include the type-II secretion system and its associated cell wall degrading enzymes, the type-III secretion system (T3SS) and its associated T3SEs, and other toxins, adhesins, transporters, signaling pathways, and regulators (Büttner and Bonas, 2010; Ryan et al., 2011; Jacques et al., 2016; Timilsina et al., 2020). T3SEs in *Xanthomonas* can be further broken down into canonical T3SEs, which are both structurally and functionally diverse, and transcription activator-like effectors (TALEs), which directly modulate gene expression to make conditions favorable for the pathogen (Ryan et al., 2011). Both canonical T3SEs and TALEs can elicit ETI via plant resistance genes (R-genes), while TALEs can also activate plant executor genes (E-genes) (Ji et al., 2022). The prevalence and distribution of these critical virulence factors across the *Xanthomonas* genus can help us understand their role in modulating virulence and host range in this critical pathogen, while also providing novel insight into host-pathogen coevolutionary dynamics.

Over the past decade, an explosion of whole-genome sequencing data from a diverse collection of *Xanthomonas* strains has paved the way for the development of a more comprehensive description of the diversity within the genus, a more fine-scale phylogenetic analysis of the relationships between species, and a detailed evolutionary framework for tracking virulence gene evolution across strains. There are now more than 2,000 *Xanthomonas* genomes available from the National Center for Biotechnology Information (NCBI), many of which are accompanied by geographical, host, and disease metadata. Here, we perform a pangenome analysis of 1,910 *Xanthomonas* strains to explore the genus-wide genetic diversity and use this data to generate a genus-wide phylogeny. We then reclassify strains into species clusters using a phylogenetic framework and identify gaps in sampling via rarefaction analyses. We also analyze the distribution and evolution of key virulence-associated genes, focusing on the T3SSs and T3SEs that play a critical role in host specificity, and quantify both ancestral and recent recombination rates for all gene families. This refined phylogenetic framework enhances our ability to explore host range evolution in the *Xanthomonas* genus and assess the relative role of different virulence factors in determining host specificity.

## 2. Materials and methods

### 2.1. Genome collection and quality control

We downloaded FASTA formatted genome assemblies for all 1,940 *Xanthomonas* genomes available on NCBI in the summer of 2021, along with the metadata associated with each genome (host of isolation, geographical location, disease phenotype). These strains were assigned to 35 distinct species designations and included 40 assigned type strains, 34 assigned pathotype strains, and 5 assigned NCBI reference strains. Any species designation that has not been validly published based on the International Code of Nomenclature of Prokaryotes (ICNP) is annotated with quotations throughout this manuscript. We also downloaded the genome assembly of *Xylella fastidiosa* strain 9a5c for comparative purposes. To annotate and identify all coding sequences in each of these genomes with a consistent and reliable pipeline, we first ran Prokka v1.14.5 on each assembly, which coordinates five external feature prediction tools (Prodigal, RNAmmer, Aragorn, SignalP, and Infernal) for gene identification and uses a BLAST approach to assign a function to each gene (Laslett and Canback, 2004; Lagesen et al., 2007; Hyatt et al., 2010; Kolbe and Eddy, 2011; Petersen et al., 2011; Seemann, 2014). In addition to Prokka’s default settings, we used “rfam” to search for ncRNAs, enabled “addgenes” to add gene features for each CDS, and used the option “compliant” to force compliance with GenBank (which also sets the minimum contig length to 200). We then evaluated the quality of each genome assembly using Quast v5.0.2 in order to quantify the genome size, N50, GC content, and number of contigs in each assembly using default settings and used this information to identify low-quality genomes that should be discarded before proceeding with further analyses (Gurevich et al., 2013).

All *Xanthomonas* strains that had low contiguity (>2,000 contigs; 7 genomes), had an unusually high or low number of coding sequences (<2,800, >5,000, 8 genomes), had a GC content distinct from the typical *Xanthomonas* range (<60, >72%, 2 genomes), or were duplicates of other strains in the collection were discarded (same name, 5 genomes). Following the exclusion of these strains, we also used an initial pangenome analysis (see below) of the remaining 1,918 *Xanthomonas* strains and *X. fastidiosa* 9a5c to identify any additional genomes that were of questionable validity. This resulted in the removal of 8 additional genomes that we were not sufficiently confident belonged in the *Xanthomonas* genus because they formed long and isolated branches that were phylogenetically distant from all validly published early branching and late branching strains. Specifically, these genomes included four *Xanthomonas* strains with no species designation (XNM01, WM.035, 60, AmX2), two “*X. massiliensis*” strains (SN8, 01534), one “*X. retroflexus*” strain (Sp953), and one incorrectly assigned *X. citri* strain (XC01). In summary, after these quality control steps, we were left with 1,910 high-quality *Xanthomonas* genomes that were used for all analyses presented in this study (Supplementary Dataset S1).

### 2.2. Pangenome analyses

To perform a genus-wide pangenome analysis for *Xanthomonas*, we classified all genes in the 1,910 *Xanthomonas* genomes and *X. fastidiosa* 9a5c into orthologous families using PIRATE (Pangenome Iterative Refinement and Threshold Evaluation) v1.0.4 with default settings (Bayliss et al., 2019). PIRATE clusters gene families over a range of thresholds to differentiate orthologs, paralogs, and putative fission/fusion events, which makes it especially well-suited to analyze our multi-species dataset. Once all gene families were clustered, we used the PIRATE output to assess the distribution of each family across all xanthomonads and established the hard-core (present in all strains), the soft-core (present in more than 95% of strains), and the pangenome (present in at least one strain) of the genus. We also ran PIRATE individually on each *Xanthomonas* species by sub-setting our genome assemblies once phylogenetically informative species designations had been established (see below).

A rarefaction analysis for the *Xanthomonas* genus was performed using the gene family presence-absence data from PIRATE (Bayliss et al., 2019) and an in-house R script to extract the number of core and accessory gene families present as genomes from the dataset were sequentially sampled (Script S1). This analysis was repeated 1,000 times to generate the genus-wide rarefaction curves and summarize the *Xanthomonas* pangenome. This same pipeline was also used to perform a rarefaction analysis, generate rarefaction curves, and summarize the pangenome of each *Xanthomonas* species that contained more than two sequenced strains (Script S2). Here, however, curves were generated with only 500 iterations. We then used the “heaps” function in the R package “micropan” (Snipen and Liland, 2015), which fits a Heap’s law model to rarefaction curves to estimate the alpha parameter and evaluate the openness of pangenomes. For both the genus as a whole and individual species with more than two strains, the number of permutations was set to 500. Finally, to estimate the average number of new gene families sampled per strain after the *Xanthomonas* pangenome had stabilized, we calculated average number of new gene families added per genome after 1,000 strains had been sampled.

### 2.3. Phylogenetic analyses

We used the set of 1,908 soft-core genes present in 95% of our strains as the initial input for our core-genome phylogenetic analysis. First, we used an in-house Python script to translate the concatenated nucleotide alignment obtained from PIRATE into a concatenated amino acid alignment (Script S3). We then filtered this concatenated alignment using Gblocks v0.91.1 using default settings to extract phylogenetically informative sites (Castresana, 2000). Finally, we used FastTree v2.1.10 to generate an approximate maximum-likelihood core-genome phylogenetic tree (Price et al., 2010). This genus-wide core-genome phylogenetic tree was used to re-classify strains into 30 monophyletic species based on the branching patterns observed, and PIRATE was run separately on each of the species that contained more than two strains in order to generate species-level core-genome trees. The same pipeline described above for the whole genus (PIRATE > GBlocks > FastTree) was used to generate these species-level trees, using the concatenated soft-core nucleotide alignment obtained from the PIRATE analysis for each species.

In addition to our basic core-genome trees, we also created a pan-genome content tree for *Xanthomonas* using the binary presence-absence matrix of all ortholog families identified by PIRATE as input for FastTree v2.1.10 (Price et al., 2010). Here, we filtered out any families that were present in all *Xanthomonas* genomes or no *Xanthomonas* genomes (only present in *X. fastidiosa* 9a5c) before running FastTree with default settings (Price et al., 2010). Finally, we generated a subset core-genome tree with only one representative strain per species to use in summary figures. Here, representative strains were extracted from the core-genome alignment and used to generate the tree with Gblocks and FastTree, as described above (Castresana, 2000; Price et al., 2010). All trees were visually enhanced using iTOL and branches with less than 50% bootstrap support were collapsed (Letunic and Bork, 2021).

### 2.4. Average nucleotide identity analysis

In order to verify the species designations assigned by our phylogenetic classification approach, we also conducted a pairwise ANI analysis to group strains at a traditional cut-off of 95% genome-wide ANI. Specifically, ANI was calculated for all pairwise combinations of *Xanthomonas* and *Xyella* strains in our collection using FastANI v.1.33 with default settings (Jain et al., 2018). Whole-genome FASTA files from each of our genomes were used as input. Pairwise ANI values calculated by FastANI were then parsed to cluster all strains that shared either direct or transitive ANI of greater than 95% into the same species group.

### 2.5. Virulence associated gene identification

To assess the prevalence and distribution of general virulence factor categories across the *Xanthomonas* genus, we downloaded protein sequences of a suite of known and predicted bacterial virulence factors from the Virulence Factor Database (VFDB) (Liu et al., 2022). We then ran a protein blast analysis (BLASTP) (v2.13.0) where the collection of known and predicted virulence factors from the VFDB were used as queries and the coding sequences from each *Xanthomonas* genome were used as subjects (Altschul et al., 1990). All *Xanthomonas* gene hits with an *e*-value of less than 10^−10^ and coverage of at least 50% were assigned to the corresponding category of virulence factors. The total number of virulence factor genes belonging to each category was then calculated for each strain, and used to quantify the average number of genes in each category for each species.

Given our interest in the T3SS and their associated T3SEs, we also independently characterized the distribution of different forms of the T3SS across the genus. Specifically, we used a collection of 12 representative structural genes from the T3SS, including both Hrp (hypersensitive response and pathogenicity) and Hrc (hypersensitive response conserved) proteins to identify T3SS islands in each *Xanthomonas* genome. These 12 structural proteins included HrcC, HrcJ, HrcN, HrcQ, HrcR, HrcS, HrcT, HrcU, HrcV, HrpB1, HrpD5, and HrpF (Supplementary Table S1). Each of these representative structural proteins was queried against all protein sequences of each *Xanthomonas* genome using BLASTP (v2.13.0) and the corresponding gene was considered present if the *e*-value was less than 10^−5^ (Altschul et al., 1990). A functional T3SS was considered present if at least 8 of the 12 structural genes were identified in the genome within the same local context (or two local contexts that were separated into two contigs). For genomes that had two T3SSs, we also partitioned T3SS structural genes based on their location in the assembly.

In order to analyze the diversity in T3SSs across *Xanthomonas* strains, we then aligned all genes from each family using MUSCLE v5.1 with default settings (Edgar, 2021). These families were then concatenated to generate one contiguous alignment per T3SS and used to generate a T3SS tree with FastTree v2.1.11 and iTOL (Price et al., 2010; Letunic and Bork, 2021). This T3SS tree enabled us to identify clusters of T3SS diversity in *Xanthomonas* based on their phylogenetic relationships. We then visualized the genomic architecture of each cluster using CLINKER v0.0.27, with one representative T3SS per cluster (Gilchrist and Chooi, 2021). Specifically, we visualized the T3SS islands from the following strains: *X. albilineans* strain LKA070 for the “Xal T3SS,” *X. campestris* strain ATCC33913 for the “Xca T3SS,” *X. translucens* strain XtKm33 for the “Xtr T3SS,” and *X. phaseoli* strain CFBP412 for the duplicate “Xca” and “Xal T3SSs.”

The distribution of all T3SEs that are secreted by these T3SSs were identified using a curated set of representative effectors belonging to all known T3SE families from the *Xanthomonas* resource^1^ and a collection of studies reporting the discovery of new T3SEs. Specifically, we used our collection of representative T3SEs to perform a BLASTP analysis (v2.13.0) where representative T3SEs were used to query all proteins in each *Xanthomonas* genome. We assigned a protein as a T3SE family if there was a hit with an *e*-value of less than 10^−18^ and a coverage of over 40%, cutoffs that we have optimized extensively for the identification of T3SEs. Once all putative T3SEs from each genome were extracted, we classified all T3SEs into families using consistent criteria using with an all-vs-all BLASTP analysis. Specifically, all T3SEs with pairwise *e*-values of less than 10^−15^ and pairwise coverages greater than 40% were clustered into the same family. This groups all T3SEs that share a direct or a transitive relationship with other T3SEs into the same family (i.e., if A was similar to B and B to C but not A to C, all three would end up in the same family). We found that although transitive relationships are vanishingly rare, they are sometimes required to recapitulate existing T3SE family designations that fall just below the thresholds used above. This final collection of T3SEs and their corresponding family assignments were used to quantify the number of effectors in each *Xanthomonas* genome and to study their prevalence at both the species and strain levels.

### 2.6. Detection and quantification of recombination

We used FastGear v1.0.0 to detect HGT across the *Xanthomonas* genus due to its efficiency in handling large datasets and its high sensitivity for detecting recombination events between divergent taxa (Mostowy et al., 2017). Specifically, we ran FastGear on each individual gene family alignment generated by PIRATE v1.0.4 that had an average of 1.25 or fewer gene copies per genome and a presence in at least 10% of *Xanthomonas* strains. We set a variable upper bound to the number of clusters in FastGear (10, 15, 20, 30, 50, 70, 100) as the gene families contained a wide range of sequences (191 to 1910). We also set the “Run clustering for all upper bounds” option to “no,” but used default settings for all other parameters. As we are more likely to detect recombination in longer gene sequences and families that are more broadly distributed, we calculated the normalized recombination rates for each family by dividing the number of recombination events detected by the gene length (in kb) and the number of pairwise combinations of gene sequences in each family. We also annotated each cluster as either virulence associated or non-virulence associated based on the results of our VFDB analysis and used a Wilcoxon rank-sum test to test whether there was a significant difference between the recombination rates of virulence associated and non-virulence associated genes. Finally, a linear regression between recent and ancestral recombination rates was performed for both virulence-associated and non-virulence associated genes to test whether these patterns are conserved through evolutionary time.

## 3. Results

### 3.1. Genome assemblies and annotations

During the summer of 2021, we downloaded all genomes classified as *Xanthomonas* (1,940) from NCBI along with *Xylella fastidiosa* strain 9a5c to use for comparative purposes. Among the *Xanthomonas* strains, 40 were labeled as type strains, 34 were labeled as pathotype strains, and 5 were labeled as NCBI reference strains. A total of 35 species designations were given to the various *Xanthomonas* strains in this collection. In order to quality control our collection, we first amalgamated all of the metadata available for each strain and evaluated various quality control metrics for each genome assembly, including N50, the number of contigs, the GC-content, and the total genome length (Gurevich et al., 2013). We also reannotated the genes from each genome using Prokka v1.14.5 to ensure consistency in the annotation pipeline (Seemann, 2014). Our initial quality control analysis led us to discard 17 genomes: 7 genomes were discarded because of a lack of contiguity (>2,000 contigs), 8 genomes were discarded because they harbored an abnormally high or abnormally low number of CDSs for *Xanthomonas* (<2,800, >5,000), and 2 were discarded because they had abnormally low GC-content for *Xanthomonas* (<60%). We also discarded 5 genomes that were duplicates of other strains already included in the study based on their strain names and 8 genomes that do not appear to be part of the *Xanthomonas* lineage based on our initial phylogenetic analysis of 1,918 *Xanthomonas* genomes and *X. fastidiosa* 9a5c. Our final collection presented here includes 1,910 *Xanthomonas* genomes and *X. fastidiosa* 9a5c.

**Figure 1:**
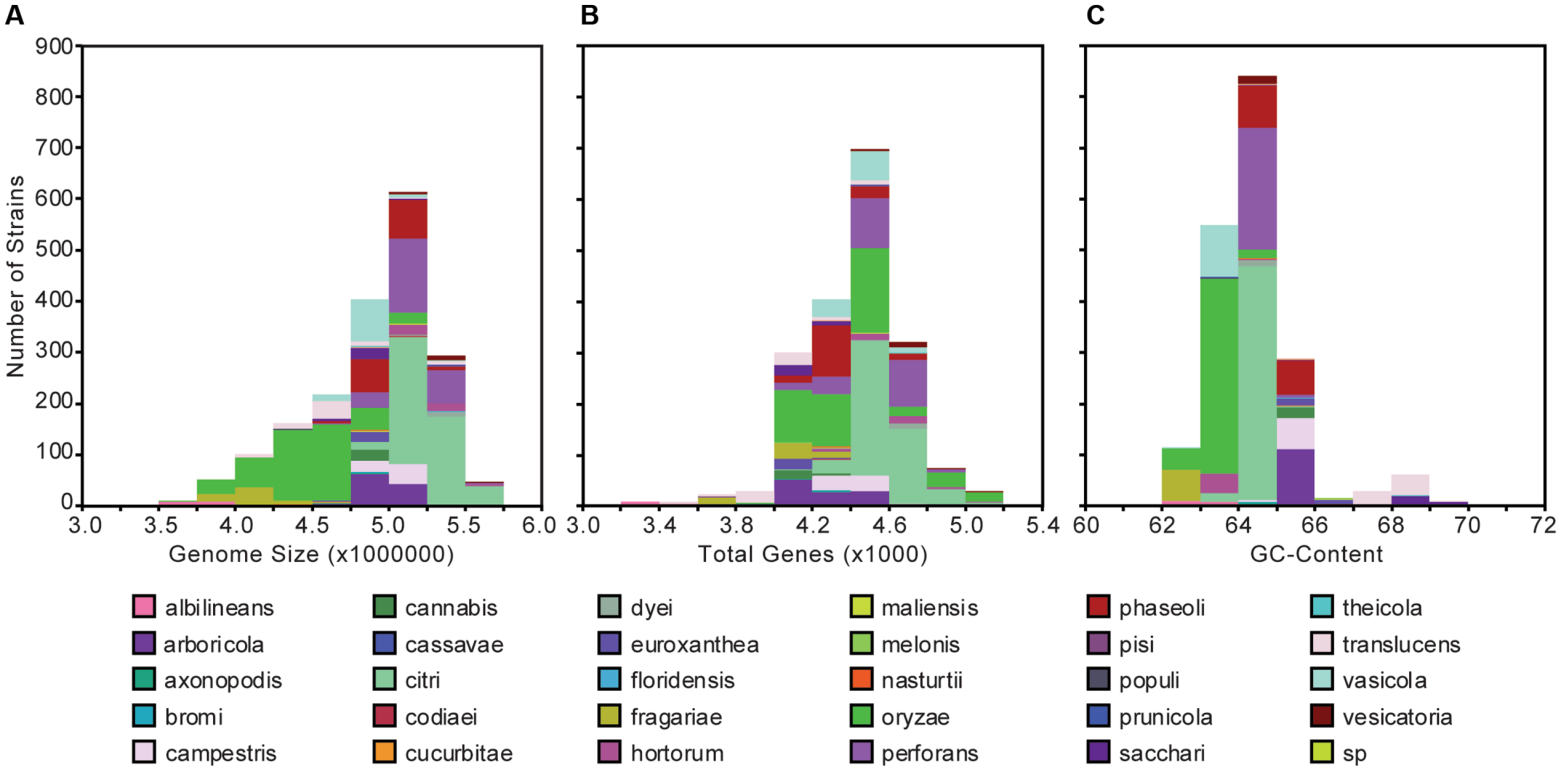
Genome size **(A)**, gene content **(B)**, and GC-content **(C)** distributions for the 1,910 *Xanthomonas* genomes analyzed in this study. Bars are colored by species.

The 1,910 *Xanthomonas* genomes analyzed in this study were derived from at least 184 unique host plants and 211 different geographical locations encompassing 99 countries around the world (Supplementary Dataset S1). The genome size of individual *Xanthomonas* strains varies from 2,731,750 to 5,770,411 base pairs (bps), with an average of 4,903,136 bps, but is mostly conserved within species (Figure 1A; Supplementary Dataset S1). Similarly, the genetic content harbored by different strains of the same species is relatively consistent, but there is much more diversity at the genus level, where individual *Xanthomonas* strains can harbor anywhere from 3,139 and 5,263 genes per strain (Figure 1B; Supplementary Dataset S1). Finally, while all *Xanthomonas* strains analyzed in this study have a high GC-content (>62%), the GC-contents of *X. translucens* strains (67%) and *X. sacchari* strains (68%) from Clade 1 are especially high (Figure 1C; Supplementary Dataset S1).

### 3.2. Pangenome analysis

**Figure 2:**
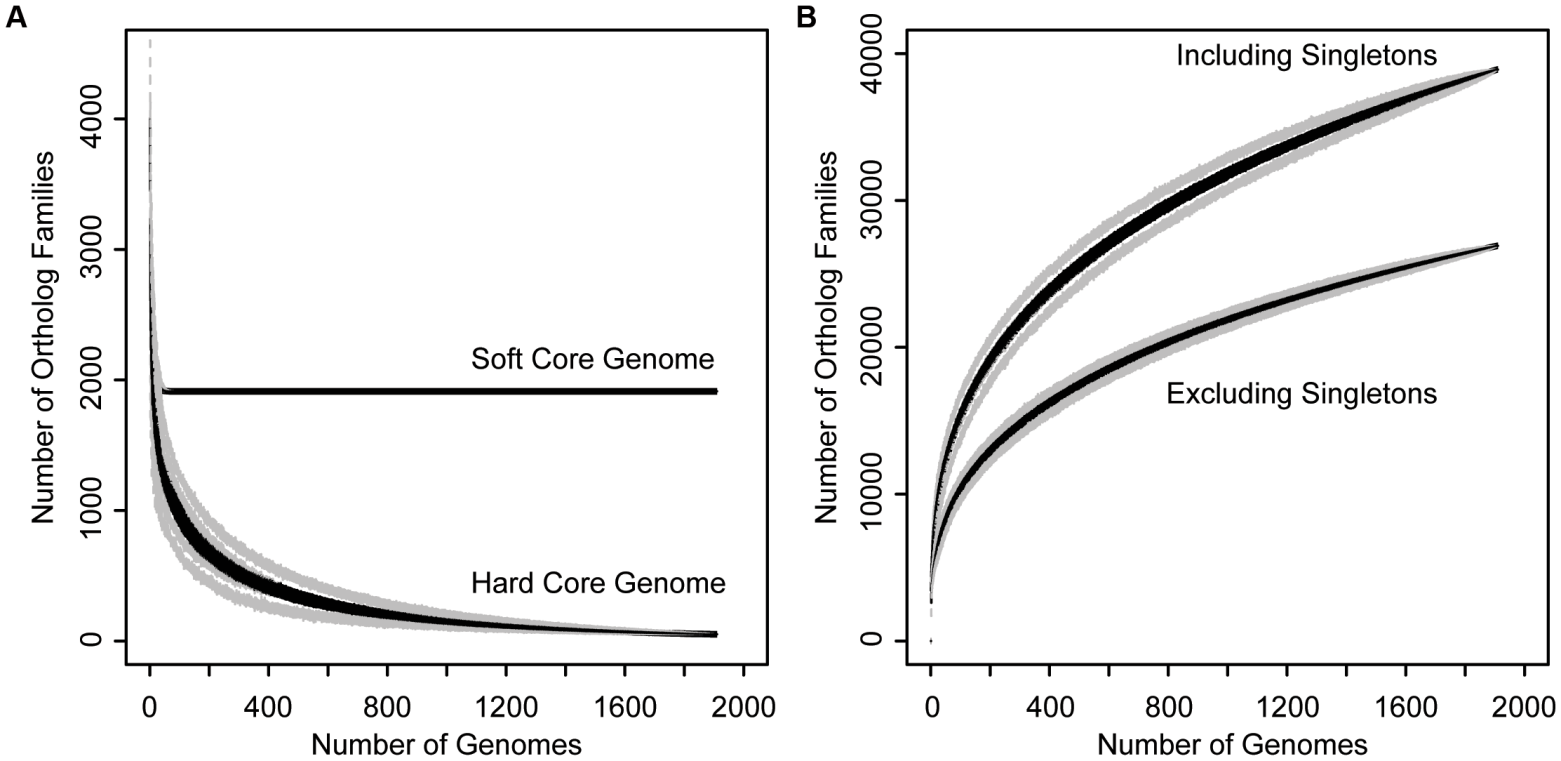
Rarefaction curves for the core **(A)** and accessory **(B)** genome of the *Xanthomonas* genus. **(A)** Families present in 95% (soft core genome) and 100% (hard core genome) of *Xanthomonas* strains decay as more genomes are added to the analysis. **(B)** The size of the *Xanthomonas* pangenome increases indefinitely as more genomes are added to the analysis, especially when singletons are included, suggesting that *Xanthomonas* has an open pangenome.

In order to assess genetic content, divergence, and cohesiveness of the *Xanthomonas* genus, we conducted a genus-wide pangenome analysis and independent pangenome analyses for each classified *Xanthomonas* species that harbored at least two sequenced strains. First, we used PIRATE v1.0.4 to cluster all *Xanthomonas* genes into orthologous families and assess the distribution of these families across strains (Bayliss et al., 2019). We then performed a rarefaction analysis on each pangenome and used the micropan package in R to estimate pangenome openness using Heap’s Law (Snipen and Liland, 2015). The *Xanthomonas* pangenome consists of 38,914 orthologous gene families, 26,910 of which are present in more than one strain. The hard-core genome (genes present in all *Xanthomonas* strains) consists of only 52 genes, though this number can be influenced considerably by the contiguity of genome assemblies in the analysis, with even high-quality draft assemblies missing a small subset of the genetic content in each strain. Therefore, a better estimate of the true core-genome size is achieved by looking at the soft-core genome (genes present in 95% of strains). Here, we find a soft-core genome size of 1,913 genes, which stabilizes after only about 100 genomes have been sampled (Figure 2A). The relative stability of the soft-core genome after this point suggests that this suite of 1,913 soft-core genes would be unlikely to significantly change with further *Xanthomonas* strain sampling. On the other hand, the accessory genome of *Xanthomonas* is highly diverse, with nearly 10 new gene families being identified in each new genome, even after 1,000 strains have been sampled (Figure 2B). Indeed, we estimated an alpha parameter of 0.60 using Heap’s Law for the *Xanthomonas* pangenome. This is considerably less than the threshold value of 1.00, suggesting that there are still many new *Xanthomonas* accessory genes to discover, even after exploring nearly 2,000 genomes. While our pangenome size does decline considerably when we exclude singletons (genes present only one genome), we still have a large pangenome size of 26,910 genes and an open pangenome (α = 0.64) (Figure 2B).

The number of strains available to assess the pangenome content of individual *Xanthomonas* species varies dramatically in both number (total strains) and diversity (number of independent studies/sampling sites) (Table 1; Supplementary Figure S1). Therefore, we need to be careful when comparing *Xanthomonas* species pangenomes since they are heavily influenced by variable sampling. When focusing on species for which we have sampled at least 50 genomes, we do see relatively consistent convergence on a soft-core genome size of between 3,000 and 3,500 genes (Table 1). As was the case with xanthomonads as a whole, the soft-core genome sizes of these species have stabilized and would be unlikely to change significantly with further sampling (Supplementary Figure S1). One interesting exception is *X. oryzae*, which has a considerably smaller soft-core genome size (2,446) but also a smaller pan-genome size (8,779), despite the fact that we included 441 *X. oryzae* strains in our analysis. A small soft-core genome size is indicative of more diversity in conserved genes, while a small pan-genome size is indicative of less diversity in accessory genes. Overall, this suggests that the evolutionary forces operating on core and accessory genes of *X. oryzae* different than they are in other xanthomonads, possibly due to less frequent horizontal transfer in the accessory genome. While estimates of the alpha parameter for the openness of the pangenome vary from 0.53 to 1.67 across *Xanthomonas* species, all species where more than 50 genomes were sampled have an open pangenome (α < 1) (Table 1). Although we emphasize again that pangenome size is heavily influenced by the number of genomes included in the analysis, species with the lowest alpha values, like *X. arboricola* (0.5295) and *X. citri* (0.5350), would be one rich resource for identifying novel *Xanthomonas* gene families.

**Table 1:** Rarefaction analysis results for the core and accessory genomes of each *Xanthomonas* species.

Species	Genomes	Hard-core genome^a^	Soft-core genome^b^	Hard pan-genome^c^	Soft pan-genome^d^	Heap’s law (α)
*Xanthomas* sp.	1,910	52	1,913	38,914	26,910	0.6021
*X. albilineans*	19	2,276	2,296	4,540	3,798	0.8864
*X. arboricola*	115	2,253	3,259	11,345	8,290	0.5295
*X. axonopodis*	6	3,109	3,109	4,918	4,422	1.4030
*X. bromi*	2	4,092	4,092	4,189	4,092	na
*X. campestris*	64	2,751	3,468	8,201	6,471	0.7033
“*X. cannabis*”	21	3,373	3,462	6,174	5,313	0.7311
*X. cassavae*	1	na	na	na	na	na
*X. citri*	478	1,865	3,398	15,080	10,557	0.5350
*X. codiaei*	1	na	na	na	na	na
*X. cucurbitae*	2	3,452	3,452	3,907	3,452	na
*X. dyei*	11	3,513	3,513	5,919	5,018	0.8691
*X. euroxanthea*	22	3,277	3,469	6,657	5,183	0.6120
*X. floridensis*	1	na	na	na	na	na
*X. fragariae*	60	2,507	3,088	5,314	4,219	0.6567
*X. hortorum*	40	2,871	3,262	8,566	6,784	0.7606
*X. maliensis*	2	3,847	3,847	4,625	3,847	na
*X. melonis*	1	na	na	na	na	na
*X. nasturtii*	2	3,725	3,725	4,210	3,725	na
*X. oryzae*	441	1,307	2,446	8,779	7,141	0.7203
*X. perforans*	245	1,179	3,531	11,779	8,652	0.6084
*X. phaseoli*	150	2,419	3,469	8,091	6,883	0.8335
*X. pisi*	2	3,383	3,383	4,404	3,383	na
*X. populi*	1	na	na	na	na	na
*X. prunicola*	3	4,310	4,310	4,386	4,348	1.67339
*X. sacchari*	28	1,989	2,819	7,700	5,883	0.7755
*X. theicola*	1	na	na	na	na	na
*X. translucens*	70	1,718	2,470	10,415	7,703	0.7037
*X. vasicola*	102	2,863	3,556	7,422	5,859	0.7807
*X. vesicatoria*	16	3,478	3,478	6,183	5,112	0.8883

na, not applicable.

a Orthology families present in all strains.

b Orthology families present in ≥95% of strains.

c All orthology families.

d Orthology families found in >1 strain (non-singletons).

### 3.3. Phylogenetic analysis

We generated a comprehensive core-genome phylogenetic tree for the *Xanthomonas* genus using a concatenated amino acid alignment of the 1,908 soft-core genes that were present in at least 95% of the 1,910 *Xanthomonas* strains used in this study. Initial species assignments were based NCBI BioSample database entries, if available (Supplementary Figure S2). We used our core-genome phylogenetic tree to assign all strains a monophyletic species designation based on the consensus identity of the strains in each lineage. Specifically, if a previously designated species was separated into multiple distinct lineages, the lineage with a greater representation of strains maintained the originally designated species name. Strains in other lineages were reassigned to the species that corresponded with their location on the phylogenetic tree. If a single lineage contained multiple species designations that did not form monophyletic groups, we assigned all strains in that lineage to the species designation that was given to more strains in the lineage, unless it had already been used elsewhere on the phylogenetic tree. Species designations were also assigned to strains that had not previously been assigned to a species, as long as they were part of a monophyletic lineage with assigned strains branching prior to the unassigned strains. In all cases, we subsequently verified that all type strains had maintained their original species designation and we retained the original pathotype designation of all strains where one was available. In total, we reclassified 288 strains, with 5 species being completely subsumed by others. Specifically, the only strain classified as *X. alfalfae* was reassigned to *X. perforans*, the only strain classified as *X. gardneri* was reassigned to *X. hortorum,* the only strain classified as *X. hyacinthi* was reassigned to *X. translucens*, all three strains classified as “*X. sontii”* were reassigned to *X. sacchari*, and all 68 strains classified as *X. euvesicatoria* were reassigned to *X. perforans.* All reassignments of strain species designations are summarized in Supplementary Dataset S1 and illustrated on Supplementary Figure S2.

We also verified all phylogenetic species assignments described above with a more traditional ANI-based approach. Specifically, after calculating ANI for all pairs of genomes in our analysis, we grouped strains that shared direct or transitive ANIs of greater than 95% and cross-referenced these groups with our phylogenetic species assignments (Supplementary Figure S2). We found that at this cut-off, ANI groups largely supported our phylogenetic species assignments, with a few exceptions. On one hand, an ANI cut-off of 95% results in the merging of the *X. citri* and *X. perforans* lineages into a single species group (Supplementary Figure S2). On the other hand, *X. sacchari* (13, 7, 4, 2, 2) and *X. translucens* (60, 7, 1, 1, 1) were separated into five groups, while *X. albilineans* (17, 2), *X. arboricola* (113, 2), *X. dyei* (10, 1), *X. euroxanthea* (21, 1), and *X. hortorum* (39, 1) were separated into two groups (Supplementary Figure S2). However, these species subgroups do not represent cases of unassigned strains that were pooled into the same species during our phylogenetic species delimitation process (i.e., they were previously assigned to the corresponding species on NCBI).

Our initial reclassification of all strains designated as *X. alfalfae*, *X. euvesicatoria*, *X. gardneri*, and *X. sontii* into alternative species groups was also well-supported by our pairwise ANI analysis at a threshold of 95% (Supplementary Figure S2). Specifically, all strains initially classified as both *X. alfalfae* and *X. euvesicatoria* formed a single group with *X. perforans*. Strains initially classified as *X. gardneri* and *X. hortorum* also formed a single group at this threshold, with *X. gardneri* str. ATCC19865 sharing as much as 99.87% ANI with *X. hortorum* strains. Finally, although there are multiple *X. sacchari* groups defined at a threshold of 95% ANI, all three *X. sontii* strains are part of the largest group, which includes 13 *X. sacchari* strains. These *X. sontii* strains also share as much as 99.94% ANI with named *X. sacchari* strains. The only reclassified species that was not supported by our ANI analysis was the reclassification of *X. hyacinthi* CFBP1156 to *X. translucens* (maximum ANI of 93.43%). However, the *X. hyacinthi* lineage is nested between two lineages that currently contain strains classified as *X. translucens* from NCBI. This is therefore consistent with the fact that in the *X. translucens* lineage, as defined in our study, there are likely multiple species-level clades. Ultimately, while any species delimitation approach on a dataset of this size is going to be sensitive to shifts in cut-offs, most of our phylogenetic species’ assignments are supported by ANI clustering analysis at 95%. However, our ANI analysis also reveals that multiple species-level clades likely exist within a subset of our classified species, particularly in early-branching clades like *X. sacchari* and *X. translucens*.

**Figure 3:**
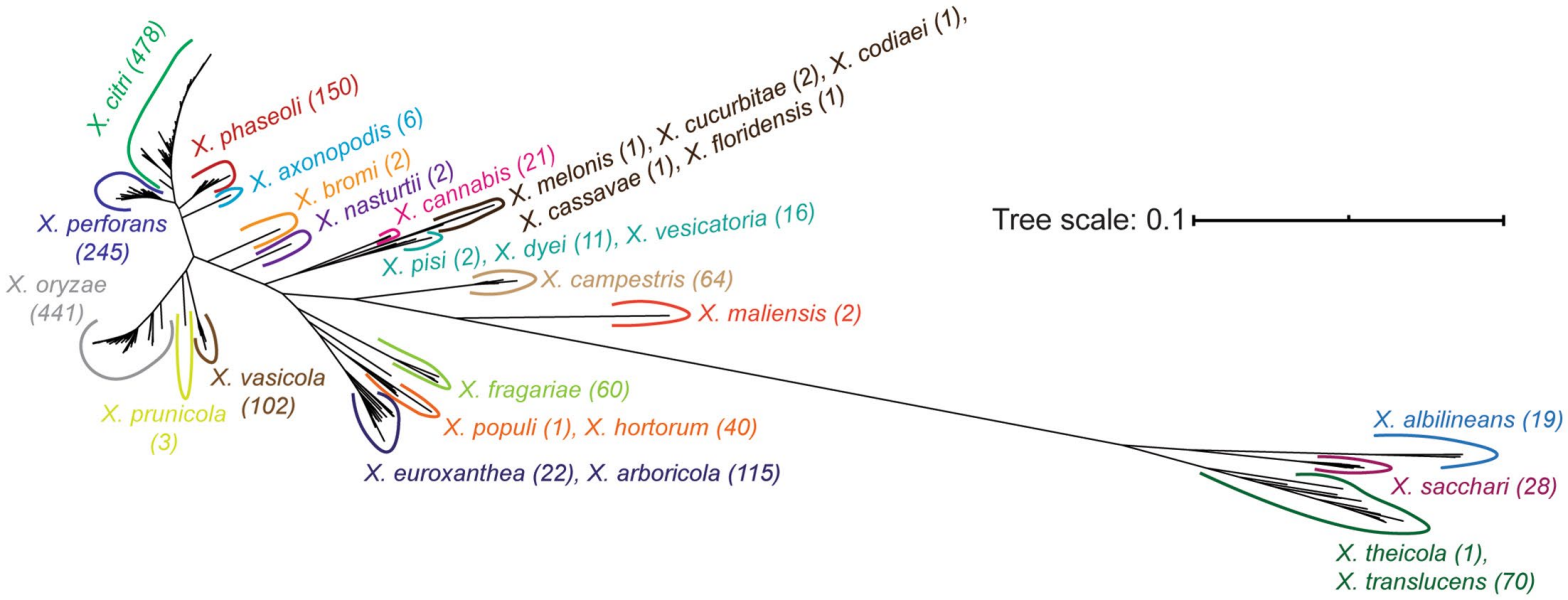
Unrooted core genome phylogenetic tree of the *Xanthomonas* strains analyzed in this study. The core genome tree was generated from an alignment of all soft-core genes present in at least 95% of the strains analyzed. All species listed on the tree form a monophyletic group independent of all other species, following the initial reassignment of species designations. A detailed core-genome phylogenetic tree with terminal leaf labels can be found in Supplementary Figure S2.

Our final core-genome phylogenetic tree first distinguishes early branching Clade 1A (*X. theicola*, *X. translucens*) and Clade 1B (*X. albilineans*, *X. sacchari*) lineages from the more closely related Clade 2 species (Figure 3; Supplementary Figure S2). Both *X. hyacinthi* and “*X. sontii”* would also have been placed in Clade 1A and Clade 1B, respectively, had the corresponding strains not been reassigned because they fell within the middle of the *X. translucens* and *X. sacchari* lineages, respectively. *X. maliensis* and *X. campestris* are the next *Xanthomonas* species to diverge in Clade 2, with subsequent divergence events involving multiple species (Figure 3; Supplementary Figure S2). Species-level phylogenetic relationships are mostly supported by the gene-content tree illustrated in Supplementary Figure S3, with two notable exceptions: *X. sacchari* strains cluster within the *X. translucens* lineage and *X. euroxanthea* strains cluster within the *X. arboricola* lineage. This may suggest that horizontal transfer in the accessory genomes of these species has overwhelmed the phylogenetic signal of vertically inherited genes, or may simply reflect the lower resolution of the accessory gene-content tree (based on presence-absence) relative to the core-genome phylogenetic tree (based on sequence-level variation).

Individual species-level trees highlight that evolutionarily divergent *Xanthomonas* strains have converged to cause disease on the same hosts and that highly similar strains can cause disease on different hosts (Supplementary Figure S4). For example, multiple Clade 2 strains from both *X. vasicola* pv. *vasculorum* and *X. axonopodis* pv. *vasculorum* have been isolated from and cause disease on sugarcane. In fact, because of their shared host ranges, these distinct species were originally grouped as *X. campestris* pv. *vasculorum*, though later taxonomic studies revealed clear genetic distinctions between these species (Studholme et al., 2020). Both *X. vaiscola* and *X. axonopodis* are also quite evolutionarily divergent from Clade 1 sugarcane pathogens *X. albilineans, X. sacchari, X. theicola*, and *X. translucens*, though their convergence on sugarcane may be at least partially tied to shared LPS biosynthesis genes that were acquired via HGT (Wasukira et al., 2014). Similarly, multiple phylogenetically distinct strains from the *X. sacchari*, *X. maliensis*, and *X. oryzae* lineages cause disease on rice, strains from the *X. phaseoli*, “*X. cannabis,” X. arboricola* and *X. citri* lineages cause disease on common bean, strains from the *X. citri*, *X. perforans, X. vesicatoria*, and *X. arboricola* lineages cause disease on pepper, strains from the *X. arboricola, X. euroxanthea, X. hortorum, X. perforans*, and *X. vesicatoria* lineages cause disease on tomato, and strains from the *X. arboricola*, *X. sacchari*, and *X. vasicola* lineages cause disease on banana (Supplementary Figure S4). We do find some examples of strains that cause disease on the same host being monophyletically clustered within a species (e.g., walnut pathogens in *X. arboricola*), but the predominant observations in species-level phylogenetic trees are that closely related strains can cause disease on distinct hosts and that host of isolation is not a monophyletic trait, (Supplementary Figure S4). While not all of these disease symptoms have been independently verified in the lab and strain labeling errors are always a possibility, the fact that multiple distinct lineages in many species have emerged to cause disease on a common host suggests that host specificity is a phenotype that has converged many times during the evolutionary history of the genus.

### 3.4. Distribution of virulence associated genes

**Figure 4:**
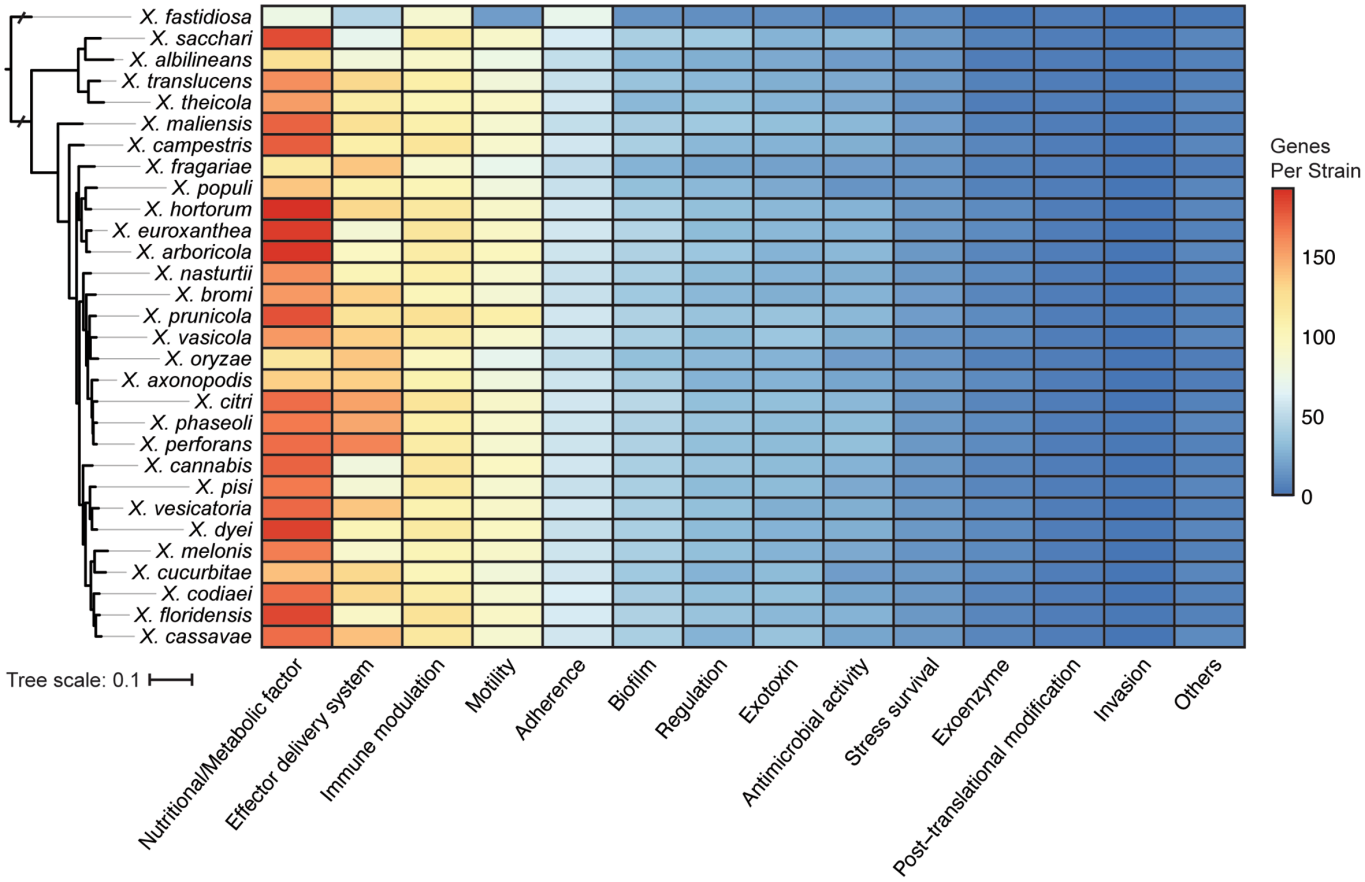
Distribution of virulence associated gene categories across the *Xanthomonas* species analyzed in this study, based on functional categories defined by the Virulence Factor Database (VFDB). The phylogenetic tree was generated using FastTree from a concatenated core-genome amino acid alignment with one representative strain for each species (see Section 2).

One obvious contributor to host specificity in *Xanthomonas* is the distribution of virulence association genes, or virulence factors, across species and strains. We first explored the diversification of broad virulence factor categories across xanthomonads by performing a BLASTP search of all proteins in the bacterial Virulence Factor Database (VFDB) against all proteins in each *Xanthomonas* genome (Altschul et al., 1990; Liu et al., 2022). A broad diversity of potential virulence factors were identified in *Xanthomonas* strains, with an especially high abundance of virulence factors associated with nutrition and metabolism, effector delivery systems, immune modulation, motility, and adherence (Figure 4). Overall, the distribution of these genes across *Xanthomonas* species was reasonably uniform, but the greatest amount of variation occurs in the two most abundant virulence factor categories. Nutritional and metabolic virulence factors are most abundant in species like *X. euroxanthea*, *X. arboricola*, *X. dyei*, *X. hortorum*, and *X. floridensis* (approximately 200 genes per strain), while *X. fragariae* and *X. oryzae* strains tend to have a smaller number of nutritional and metabolic virulence factors (approximately 120 genes per strain) (Figure 4; Supplementary Figure S5). The number of effector delivery system associated genes is also quite variable across species, with strains from *X. perforans*, *X. phaseoli*, and *X. citri* harboring approximately 150 genes each and strains from “*X. cannabis,” X. melonis, X. pisi, X. albilineans*, and *X. sacchari* only harboring approximately 80 genes each. While there is not always a phylogenetic pattern to the distribution of virulence factor categories at the species level, it’s notable that *X. perforans*, *X. phaseoli*, and *X. citri* do form a monophyletic lineage and harbor the most abundant repertoires of effector delivery system virulence factors. Furthermore, *X. fastidiosa* 9a5c has among the lowest average number of virulence factors in all categories, which suggests that most of these categories of virulence factors have expanded in *Xanthomonas* lineages writ large and/or that many have been lost in the *X. fastidiosa* lineage.

**Figure 5:**
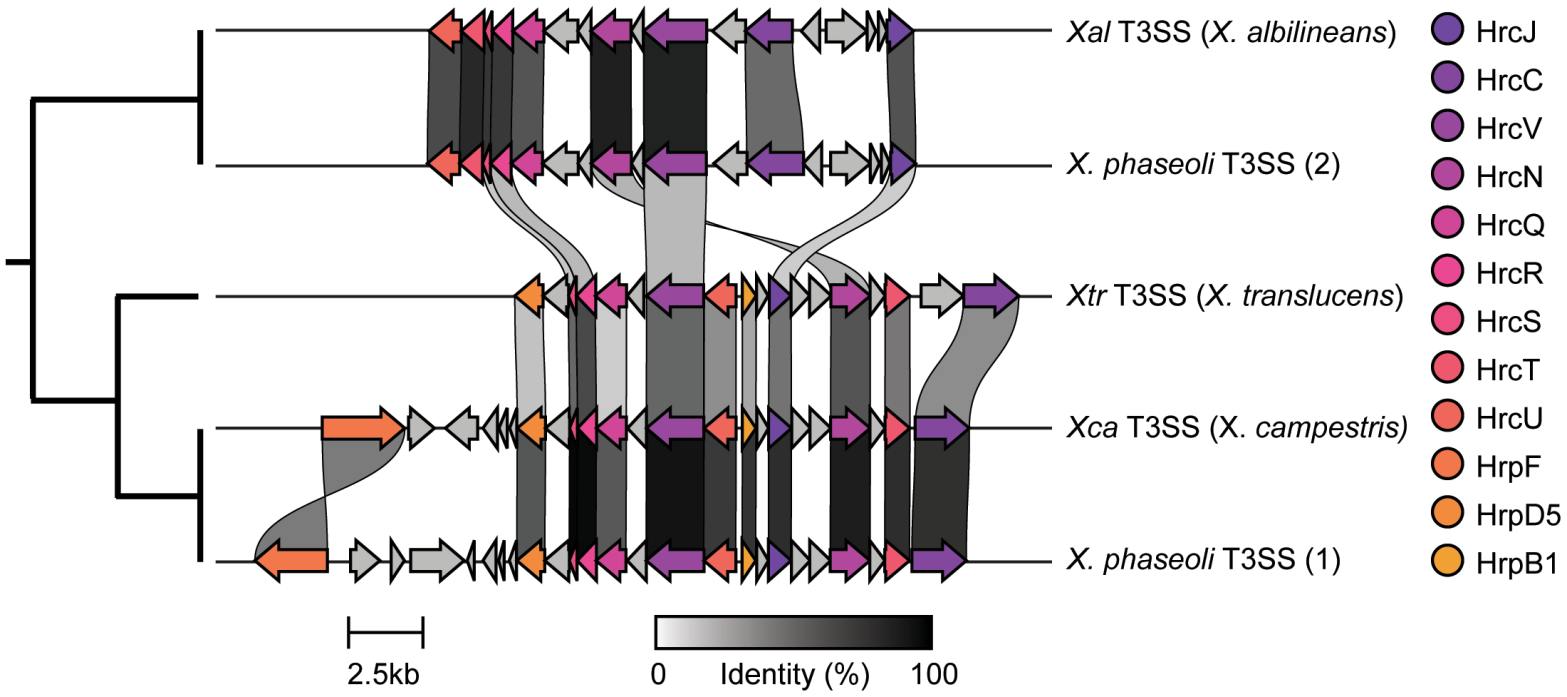
Genetic architecture of the three forms of *Xanthomonas* type III secretion systems (T3SSs) categorized in this study. The architecture for each T3SS is drawn from the following representative genomes: *Xal* T3SS—*X. albilineans* LKA070, *Xtr* T3SS—*X. translucens* XtKm33, and *Xca* T3SS—*X. campestris* ATCC33913. The two distinct T3SSs identified within some single *X. phaseoli* genomes are also displayed using *X. phaseoli* CFBP412 as a representative.

**Figure 6:**
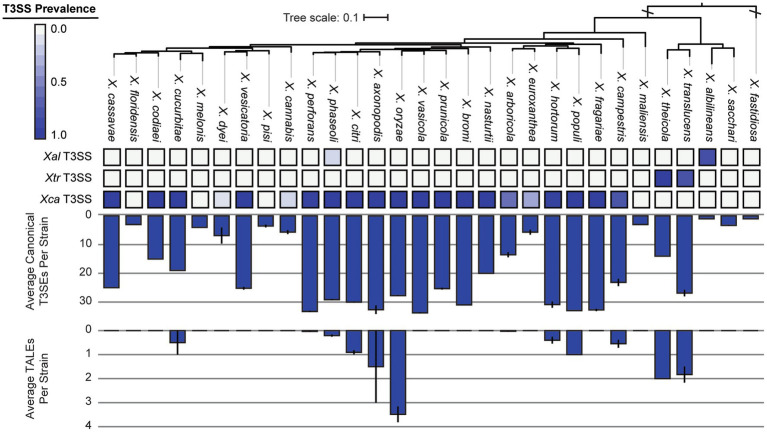
Type III secretion system (T3SS), canonical type III secreted effector (T3SE), and transcription activator-like effector (TALE) repertoires for each of the *Xanthomonas* species analyzed in this study. The phylogenetic tree was generated using FastTree from a concatenated core-genome amino acid alignment with one representative strain for each species (see Section 2). The three T3SSs analyzed include the *Xal* T3SS, which is present primarily in *X. albilineans* lineage, the *Xtr* T3SS, which is present in *X. translucens* and *X. theicola*, and the *Xca* T3SS, which is present in all other lineages. The heatmap represents the proportion of strains from each species that harbor the corresponding T3SS. The average numbers of T3SE and TALE genes per genome in each species were determined via BLASTP analysis of 57 known *Xanthomonas* T3SEs against the proteome of each genome (see Section 2). Error bars represent the SEM for all strains within a species.

We were especially interested in the T3SS and its associated T3SEs given the importance of T3SEs in determining host range as both virulence enhancers and immune elicitors (Jones and Dangl, 2006). First, in order to explore the presence-absence of different T3SSs across the *Xanthomonas* genus, we used a BLASTP analysis to search for twelve core T3SS pathogenicity island proteins in each *Xanthomonas* genome (Supplementary Table S1). We then extracted the complete repertoire of T3SS genes from each island, aligned each of the twelve focal families, and performed a phylogenetic analysis to explore the diversity of T3SSs across the *Xanthomonas* genus (Supplementary Figure S6). We identified three evolutionarily and structurally distinct versions of the *Xanthomonas* T3SS, including the *X. albilineans* T3SS (*Xal* T3SS), which is primarily found in *X. albilineans*, the *X. translucens* T3SS (*Xtr* T3SS), which is only found in *X. translucens* and *X. theicola*, and the *X. campestris* T3SS (*Xca* T3SS), which is distributed across Clade 2 *Xanthomonas* species (Figure 5; Supplementary Figure S6). While most strains that harbor a T3SS only harbor one, we did identify 22 strains from the *X. phaseoli* lineage that harbored both the *Xal* T3SS and the *Xca* T3SS (Figures 5, 6; Supplementary Figure S7). Given the phylogenetic distribution of these T3SSs, it seems likely that the *Xal* T3SS of *X. phaseoli* was acquired horizontally from *X. albilineans*, but its conservation across a number of strains begs the question of what role it plays in a genome that already harbors at T3SS. On the other hand, there are also several *Xanthomonas* strains that lack a T3SS altogether. In particular, all strains classified as *X. sacchari*, *X. maliensis*, *X. pisi*, *X. melonis*, and *X. floridensis*, and a subset of strains classified as *X. campestris*, *X. euroxanthea*, *X. arboricola*, “*X. cannabis,”* and *X. dyei* do not harbor any of the three versions of the *Xanthomonas* T3SS. This distribution of the various types of T3SS, which do not always cluster phylogenetically, demonstrates that there have likely been many gains and losses of the T3SS in the evolutionary history of xanthomonads (Supplementary Figure S7).

**Figure 7:**
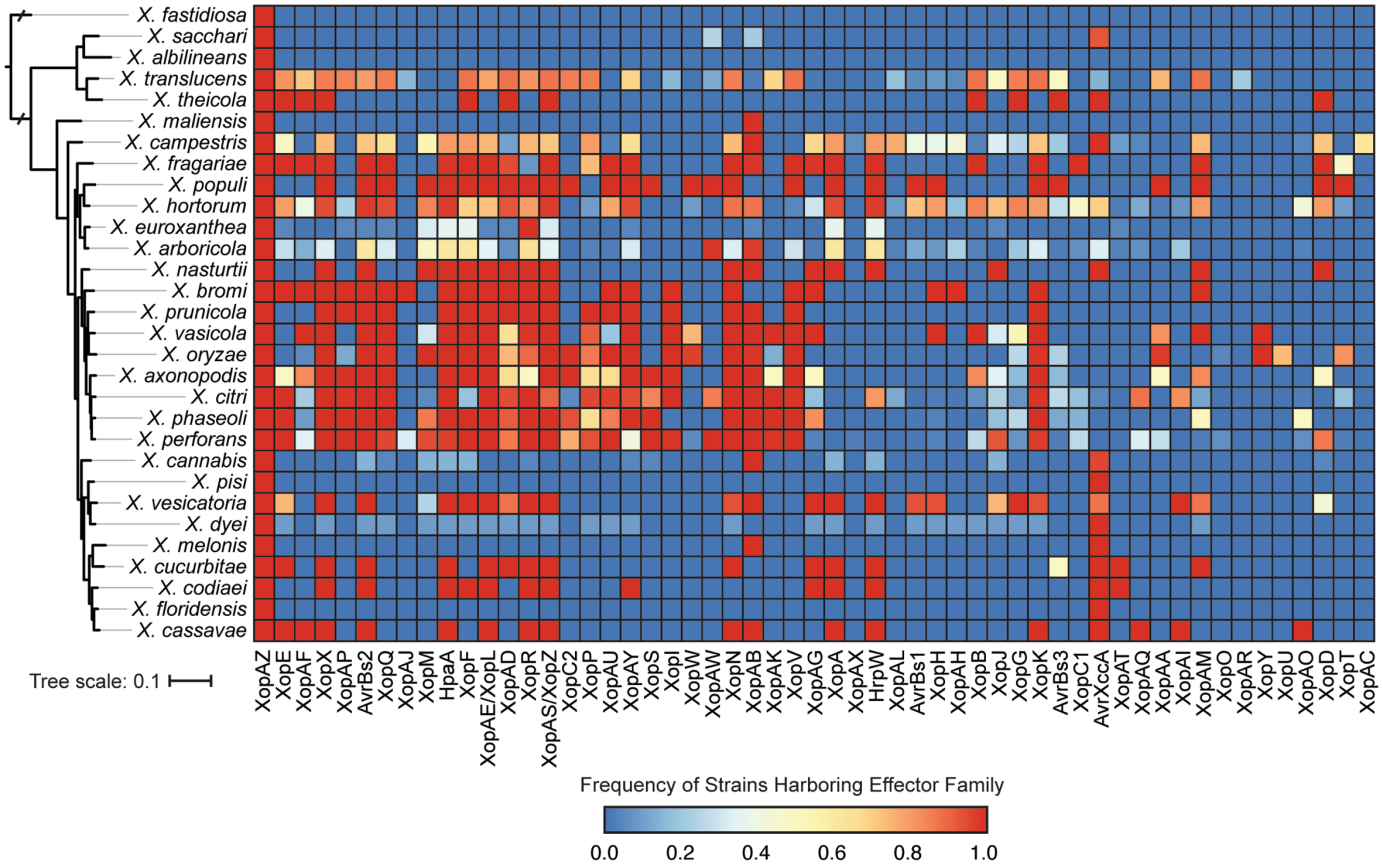
Prevalence of each type III secreted effector (T3SE) family in each of the *Xanthomonas* species analyzed in this study. Color scaling indicates the prevalence of each T3SE family within the respective species. The phylogenetic tree was generated using FastTree from a concatenated core-genome amino acid alignment with one representative strain for each species (see Section 2).

We also explored the distribution of T3SEs across *Xanthomonas* strains using a BLASTP analysis to search for a collection of representative T3SEs from 57 known families in each genome. Only a single T3SE family (XopAZ) would be considered part of the *Xanthomonas* soft-core genome, meaning it is present in more than 95% of strains. However, if we focus in on only strains that harbor a functional T3SS, AvrBs2, HpaA, and XopAB also become part of the soft-core genome. The distribution of most T3SE families is relatively mosaic, with multiple phylogenetically distinct species harboring them and only a subset of the strains in each species harboring them (Figure 7; Supplementary Dataset S2). Furthermore, some families are species specific, with XopAC only present in *X. campestris*, XopU only present in *X. oryzae*, and XopAR only present in *X. translucens*. This mosaic structure is likely the result of the frequent presence of T3SEs on mobile elements, the benefits they provide for virulence when acquired in some circumstances, and the costs they incur by eliciting the immune response in other circumstances.

We further categorize our T3SEs as either canonical T3SEs from a diverse array of families or TALEs from the *avrBs3* family. The total number of canonical T3SEs per strain varies considerably, from as few as 1 canonical T3SE in *X. albilineans* MUS060, which lacks a T3SS, to 43 canonical T3SEs in *X. citri* LMG7504. However, if we only consider Clade 2 strains determined to harbor a *Xca* T3SS, the minimum number of T3SEs per genome rises to 9 in *X. euroxanthea* strain BRIP62409. At the species level, we find that some species harbor an average of more than 30 canonical T3SEs per strain (e.g., *X. axonopodis*), while others harbor few, if any, canonical T3SEs (e.g., *X. albilineans*) (Figure 6). There are also species like *X. cucurbitae* (19.5 effectors per strain) and *X. codiaei* (15 effectors per strain) that harbor an intermediate number of T3SEs per strain. Variation in TALE content across species is also dramatic, though at least some of this variation can be attributed to differences in the quality of the assemblies in these notoriously difficult to resolve regions (Erkes et al., 2023). We only identified TALEs in a subset of *Xanthomonas* species and the average number of TALEs per strain across an entire species is never higher than 4 (Figure 6). However, there are strains of *X. oryzae* that harbor as many as 29 TALEs (*X. oryzae* pv. *oryzicola* L8), and others that harbor none (*X. oryzae* pv. *oryzae* AH1) (Figure 7; Supplementary Dataset S2). In most cases, the canonical T3SE and TALE data are consistent with the presence-absence of T3SSs, where strains that do not harbor a T3SS also harbor few, if any, T3SEs (Figure 6). However, one notable exception is *X. albilineans,* where all strains harbor the *Xal* T3SS and almost no effectors at all. Along with its maintenance as a secondary T3SS in multiple strains of *X. perforans*, this provides further support for the hypothesis that this T3SS has diverged in function and is not involved with the secretion of *Xanthomonas* T3SEs. While some of the T3SEs identified here may ultimately turn out to be pseudogenes, it is clear that there is immense variation in both the numbers and types of T3SEs between species and strains.

### 3.5. Rates of lateral gene transfer

Several analyses conducted in this study suggest that horizontal gene transfer is common in the *Xanthomonas* genus. First, while the gene content presence-absence tree mostly recapitulates the species-level relationships of the core-genome tree, there are some notable differences (e.g., *X. sacchari* clustering within the *X. translucens* lineage) (Supplementary Figure S3). There are also many changes to the branching pattern of strains within species, which is likely to be at least in part due to the horizontal exchange of accessory gene content. Furthermore, a gene family frequency distribution that quantifies the number of genomes harboring each gene family reveals a U-shape distribution, where the majority of gene families are either very rare or very common (Supplementary Figure S8). This is a characteristic that is often observed in species where lateral gene transfer is common, since more genetic content is being acquired by individual strains in the genus that is not already present in other xanthomonads.

**Figure 8:**
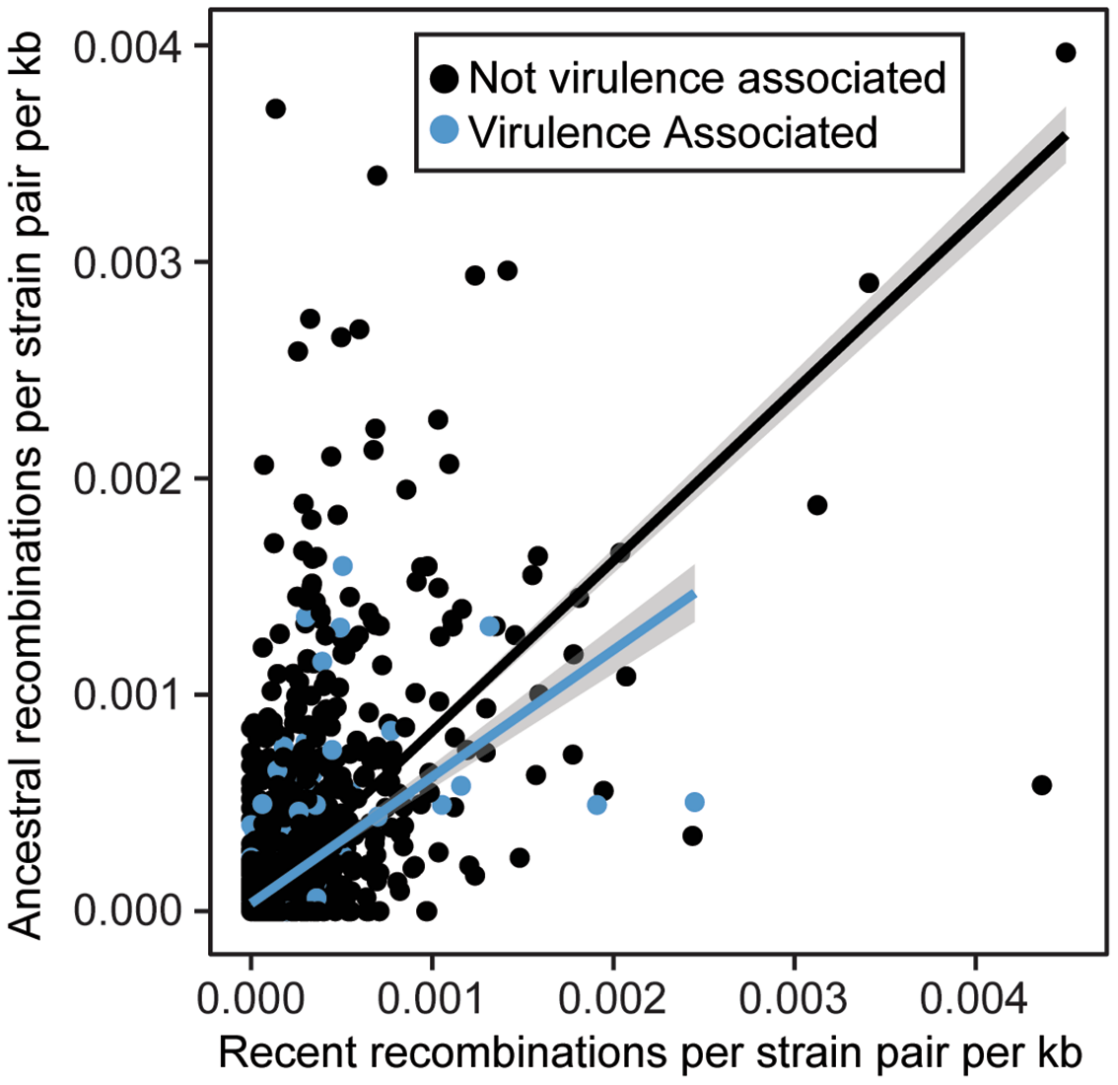
Relationship between normalized ancestral and recent recombination rates for virulence associated and non-virulence associated gene families. All recombination events were detected by FastGear and normalized rates were calculated by dividing the number of events by the size of the family (strains) and the length of the corresponding genes (kb).

We also sought to directly evaluate the frequency of recombination for each gene family using FastGear, an efficient and scalable method to detect ancestral and recent recombination events (Mostowy et al., 2017). Specifically, for the 4,990 gene families that were present in 10% of strains or more, we find that recombination events are common, with 4,671 genes (93.6%) displaying evidence of at least one recent recombination event and 4,518 gene families (90.5%) displaying evidence of at least one ancestral recombination event. There were only 319 genes families (6.4%) in which we did not find any evidence of recombination. We also evaluated the relative rates of recombination in virulence-associated vs. non-virulence-associated genes. Specifically, we normalized the number of recent and ancestral recombination events by the gene length and number of sequences in each family, as we are more likely to detect recombination in longer and more broadly distributed genes. We found that in both virulence-associated and non-virulence-associated gene families, there is a positive correlation between rates of recent and ancestral recombination (*R*^2^ = 0.37 for VFs, 0.39 for non-VFs, *p* < 2.2e-16 for both) (Figure 8). Surprisingly, we also found that virulence genes recombine at a significantly lower recombination rate than non-virulence genes (Wilcoxon signed-rank test, recent events *p* = 3.707^−11^, ancestral events *p* = 1.416^−5^), though the rates for both virulence associated and non-virulence associated genes can be high.

## 4. Discussion

Plant pathogens pose a significant threat to global food security and cause major economic losses worldwide. The recent expansion of whole-genome sequencing data for diverse plant pathogens provides a unique opportunity to study how novel pathogens emerge by conducting deep comparative genomic analyses that study the evolutionary relationships between species and diversity in their respective virulence repertoires. In this study, we performed a pangenome analysis using nearly 2,000 *Xanthomonas* strains to highlight the diversification of gene content in this genus of important plant pathogens. We then used this analysis to generate a genus-wide phylogeny that illustrates the convergent evolution of distantly related strains to the same host and to compare the mosaic virulence repertoires of each strain. Finally, we showed that many gene families in *Xanthomonas* are undergoing frequent recombination that likely contributes to convergent host specificity and enhancing genetic cohesion within the genus.

### 4.1. Genetic architecture and diversity in the *Xanthomonas* genus

Using a uniform gene prediction and annotation pipeline, we first used our expanded whole-genome dataset to explore variation in the architecture of the genomes of diverse xanthomonads that were isolated as far back as 1915 (Figure 1; Supplementary Dataset S1). Specifically, we observed a range in genome size from 3.5 to 6.0 Mb in length, a range in coding content from 3,200 to 5,200 genes, and a range in GC-content from 62 to 70%. These results were largely consistent with a number of prior studies that focused on a smaller number of species and strains. First, strains of broadly sampled species like *X. campestris*, *X. oryzae*, and *X. citri* have been reported to have relatively modest genome sizes in the range of 4.8–5.4 Mb, gene counts between 4,500 and 5,400 genes, and GC-contents of approximately 65% (Bogdanove et al., 2011; Huang et al., 2015). Our broader collection of genomes from these species is consistent with these observations, though we do identify broader ranges in these features within each species, particularly for the lower bounds. Notably, some genomes of *X. oryzae* have genome sizes smaller than 3.5 Mb and fewer than 4,000 genes. There are also genomes of *X. oryzae* that have GC-contents as low as 62% (Figure 1; Supplementary Dataset S1). Significantly reduced genomes were also identified in *X. albilineans*, *X. fragariae*, and *X. translucens*, as has been observed previously, and higher GC-contents were identified in *X. translucens* and *X. sacchari* (Fang et al., 2015; Peng et al., 2016). Reductive evolution in multiple *Xanthomonas* lineages may be indicative of an increased reliance on the host in these lineages, or it may have occurred as a consequence of reduced effective population sizes from repeated bottlenecking events. A more focused evolutionary genetic analysis to differentiate between these possibilities is an exciting future direction for this dataset.

While the *Xanthomonas* genus as a whole has a large and dynamic pangenome, consisting of nearly 40,000 orthologous gene families, it’s notable that given the number of strains that we have sampled, the size of the *Xanthomonas* pangenome is quite modest compared to other well-studied bacterial plant pathogens. Specifically, a pangenome analysis of 314 strains from the *Ralstonia solanacearum* species complex (RSSC) identified a pangenome of 31,020 pangenes (Castillo, 2023), while a pangenome analysis of 391 strains from the *Pseudomonas syringae* species complex (PSSC) identified 77,728 pangenes (Dillon et al., 2019). Both of these pangenome sizes are considerably larger than the *Xanthomonas* pangenome at a similar level of sampling (Figure 2), which is surprising given the diversity of classified *Xanthomonas* species sampled in our collection. While it is certainly the case that there are additional proposed species designations in both the RSSC and the PSSC, it’s clear that the accessory genome of *Xanthomonas* species is not as diverse as we have observed in other important phytopathogens, which will make its exploration more manageable in future studies.

Ultimately, it will be important to consider which *Xanthomonas* species are most worthy of further sampling to identify novel *Xanthomonas* accessory genes, many of which likely contribute to virulence and host specificity. While our rarefaction analyses indicate that the core genome of *Xanthomonas* is unlikely to change significantly with further sampling, the *Xanthomonas* genus as a whole, and most of the species within it, still have open pangenomes. This means that further strain sampling will yield further expansion of the *Xanthomonas* pangenome, with nearly 10 new genes being sampled per strain based on our estimates (Figure 2). Species like *X. arboricola* and *X. citri* are one category of good candidates for novel gene identification as they have alpha parameters of below 0.55, despite more than 100 strains having already been sampled from both species (Table 1). In addition, species groups analyzed in this study that appear to harbor multiple species-level clades, like *X. sacchari* and *X. translucens*, should be prioritized in diversity sampling efforts moving forward to help resolve these lineages.

### 4.2. Convergent evolution of distantly related *Xanthomonas* strains to the same host

As has been the case with many microbial species during the past century, *Xanthomonas* taxonomy has undergone substantial revision as more strains have been sampled and technologies for classifying them have improved. Some examples of recent issues include the reclassification of *X. campestris* pv. *musacearum* strains to *X. vasicola* (Aritua et al., 2008), the finding that *X. axonopodis* does not form a monophyletic taxon (Rodriguez-R et al., 2012), and the merging of *X. gardneri* and *X. cynarae* into a single species (Timilsina et al., 2019a). While our core-genome phylogenetic analysis included several *Xanthomonas* strains currently assigned to species designations that do not reflect their phylogenetic relationships and some strains that had not been assigned species designations at all, we made a concerted effort in this study to preserve as many current species designations as possible, while also ensuring that all species met the minimum requirement of forming a single monophyletic group (Supplementary Figure S2). As pointed out by Rodriguez-R et al., this required considerable renaming of *X. axonopodis* strains, some of which had to be reassigned to various other species names, including *X. citri*, *X. perforans*, and *X. phaseoli*. There were also five species that were completely subsumed by other species designations, including *X. alfalfae*, *X. gardneri*, *X. hyacinthi*, “*X. sontii,”* and *X. euvesicatoria* because they did not form monophyletic lineages. These monophyletic species designations, including the cases where species were completely subsumed by other species names, were remarkably well supported by an ANI analysis at a cutoff of 95% identity. However, our ANI analysis also reveals the existence of multiple potential species-level clades within *X. albilineans*, *X. arboricola*, *X. dyei*, *X. euroxanthea*, *X. hortorum*, *X. sacchari*, and *X. translucens*. These species level clades, particularly in early-branching species, have been well-documented in the literature and recent efforts to sequence more representative strains in these clades is a promising step toward determining the extent to which diversity is continuous or clustered in these early-branching lineages (Parkinson et al., 2009; Peduzzi et al., 2023).

Beyond the classification of species based on their core-genome diversity, one of the more significant outputs of this study is the most comprehensive phylogeny of *Xanthomonas* species sequenced to date (Figure 3; Supplementary Figure S2). While prior studies have constructed phylogenies for individual *Xanthomonas* species (Hauben et al., 1997; Gonçalves and Rosato, 2002; Parkinson et al., 2009; Rodriguez-R et al., 2012; Timilsina et al., 2020), and some have even focused on the *Xanthomonas* genus as a whole (Timilsina et al., 2020), no single study has considered nearly the breadth (species) or the depth (strains) of xanthomonads analyzed here. Two notable differences between a recent comprehensive core-genome phylogeny of representative xanthomonads and our study is that we include a broad diversity of strains from the majority of species (up to 478 in *X. citri*) and a few additional species that were more recently sequenced (“*X. cannabis*,” *X. euroxanthea*, *X. floridensis*, *X. nasturtii*) (Timilsina et al., 2020). Despite this, the structure of our tree is mostly consistent with the tree derived by Timilsina et al., with a few exceptions in the branching patterns of Group 2 *Xanthomonas* species. In combination with the monophyletic species designations described above, this ultimately paves the way for more sophisticated comparative analyses of genetic and phenotypic differences between these species.

The most obvious observation that we can make by integrating host and disease metadata with our comparative genomic analysis is that phylogenetically distinct strains will often infect the same host (Supplementary Figure S4). Indeed, with a few notable exceptions, this is evident at both the species level, where strains from entirely different taxa are isolated from the same host, and at the strain level, where strains from different lineages of the same species have converged to infect the same host. It is also clear that closely related strains are often isolated from entirely different host species, which may reflect either a broad pathogen host range or relatively frequent shifts in host specificity in the evolution of xanthomonads. One recent study has found that evolutionarily distant strains in *X. phaseoli* and *X. citri* converge to infect common bean through the horizontal transfer of a number of focal genes (Chen et al., 2018). Another found that evolutionary convergence on sugarcane may be tied to shared LPS biosynthesis genes that were exchanged via HGT (Wasukira et al., 2014). Our analysis provides the framework for characterizing the genetic mechanisms that underly a number of other host specificities, yielding further insight into the relative role of different categories of virulence factors in determining host range.

### 4.3. Abundant and rapidly evolving *Xanthomonas* virulence repertoires

The specific virulence factors involved in host specificity across diverse xanthomonads have enormous potential to be leveraged as targets for agricultural engineering of resistant crop cultivars. We first explored the distribution of different categories of virulence factors across our collection of *Xanthomonas* strains using the VFDB, finding that virulence factors involved in nutrition and metabolism, effector delivery systems, immune modulation, motility, and adherence are most abundant (Figure 4). Many of these categories have also been highlighted in prior studies of *Xanthomonas* virulence factors, which found that successful infection and multiplication of xanthomonads within hosts depend on a number of extracellular polysaccharides, degradative enzymes, adhesins, secretion systems, and effectors (Büttner and Bonas, 2010). While there is some variation in the abundance of these broad virulence factor categories in distinct *Xanthomonas* lineages, the quantity of these virulence associated genes across all *Xanthomonas* strains relative to *X. fastidiosa* is especially notable. In particular, nearly all *Xanthomonas* species have large repertoires of virulence factors associated with nutrition and metabolism, effector delivery systems, and motility. These abundant virulence factor repertoires are likely a major reason why xanthomonads are such effective independent pathogens across a broad range of hosts, though the double-edged nature of effectors that modulate immunity will inevitably contribute to reducing the host range of individual strains. (Laflamme et al., 2020)

The critical role of T3SSs and their associated T3SEs in determining host specificity across a range of gram-negative bacterial phytopathogens motivated us to explore the distribution of these critical virulence factors in more detail. When exploring the T3SS content across *Xanthomonas* strains, we identified three evolutionarily and structurally distinct T3SS pathogenicity islands (Figures 5, 6), which were likely the result of three independent acquisition events (Merda et al., 2017). The most broadly distributed T3SS in *Xanthomonas* is the *Xca* T3SS, which is present in the vast majority of Clade 2 strains, and nearly all strains that harbor this T3SS also have substantial T3SE repertoires. The presence of the *Xtr* T3SS in Clade 1A species (*X. translucens*, *X. theicola*) is also correlated with the presence of a high abundance of both canonical T3SEs and TALEs. However, the third *Xanthomonas* T3SS, dubbed in this study as the *Xal* T3SS, is present in a high frequency of *X. albilineans* strains that harbor very few, if any, T3SEs. While the *Xal* T3SS is present in a small number of *X. phaseoli* strains that harbor abundant T3SE repertoires, these strains also harbor a second canonical *Xca* T3SS. Prior evidence has indicated that this T3SS is similar to the *Salmonella* pathogenicity island 1 (SPI-1) family found in animal pathogens, illustrating that it may have been horizontally transferred to *Xanthomonas* from *Salmonella* or another source (Alavi et al., 2008; Marguerettaz et al., 2011). In any event, the fact that so few *Xanthomonas* T3SEs are observed in the strains that harbor it suggests that the role of the *Xal* T3SS does not involve the secretion of characterized *Xanthomonas* T3SEs. Several *Xanthomonas* species also lack the T3SS entirely, including *X. floridensis*, *X. melonis*, *X. pisi*, *X. maliensis*, and *X. sacchari* (Merda et al., 2017; Vicente et al., 2017). In addition to these species, we also find that a significant number of *X. dyei*, “*X. cannabis*,” *X. arboricola*, and *X. euroxanthea* strains have also lost the T3SSs (Jacobs et al., 2015). The loss of these critical virulence factors may indicate a transition to a non-pathogenic lifestyle or reliance on alternative virulence factors to infect and cause disease in plant hosts.

Consistent with the loss of T3SSs in a subset of *Xanthomonas* lineages, T3SE repertoires are also substantially reduced in a similar collection of *Xanthomonas* strains (Figures 6, 7; Supplementary Dataset S2). For example, *X. arboricola* strains with a canonical *Xca* T3SS have an average 19 T3SEs per strain, but those without a T3SS have only 4 T3SEs per strain. In sum, we identify only four soft-core T3SE families among strains that harbor a T3SS (XopAZ, AvrBs2, HpaA and XopAB). However, among these, only the AvrBs2 family has been consistently observed to be part of the core-genome of individual *Xanthomonas* species, like *X. campestris* and *X. axonopodis* (Hajri et al., 2009; Jalan et al., 2011; Potnis et al., 2011). This could be driven by stricter core-genome criteria in other studies, or the fact that a species-specific approach highlights a small number of strains in a given species that may be lacking a given T3SE family. In any event, these core T3SEs have the greatest potential for resistant crop breeding against *Xanthomonas* species broadly, as long as we can identify host resistance genes capable of detecting them (e.g., Bs2 for AvrBs2) (Swords et al., 1996).

The distribution of the remainder of T3SEs is relatively mosaic, suggesting substantial gain and loss events across the *Xanthomonas* genus. This is especially true for TALEs, which have undergone dramatic expansion in some strains and are completely absent from others (Figures 6, 7; Supplementary Dataset S2). However, it’s important to recognize that at least some of the variation we see in TALE content is going to be driven by the challenges associated with assembling these repetitive regions using short-read sequencing data (Erkes et al., 2023). In any event, both T3SEs and TALEs are capable of enhancing virulence by interfering with host resistance pathways and diminishing virulence through the elicitation of effector triggered immunity, which has a significant evolutionary impact on the strains that harbor them. Furthermore, some families of T3SEs and TALEs are especially prone to HGT because of their association with mobile elements (Jones and Dangl, 2006; Deb et al., 2022). These are likely the two primary evolutionary forces driving the dramatic variation in T3SE and TALE repertoires. Indeed, we observe an especially fragmented distribution in the XopG T3SE family (Figure 7; Supplementary Dataset S2), which has been shown to be readily exchanged between distantly related *Xanthomonas* species (Hajri et al., 2012; Deb et al., 2022). While we lack the ability to explore the diversity in TALE binding domains in this study due to our heavy reliance on short-read sequencing data, exploring TALE diversity and E-gene recognition with long-read sequencing data is an exciting avenue for future research.

### 4.4. Prevalent recombination and the maintenance of genetic cohesion

Several lines of evidence in our pangenome analysis of xanthomonads suggest that recombination is common in the genus. This includes both recombination within the *Xanthomonas* genus and recombination between xanthomonads and other bacterial species. First, we identified structural differences between our core-genome and our accessory gene content phylogenetic trees (Supplementary Figures S2, S3), suggesting that at least in some cases, sufficient accessory gene content has been exchanged to yield an alternative phylogenetic picture. One clear example of this is the merging of Clade 1B strains of *X. sacchari* and Clade 1A strains of *X. translucens*, which formed distinct lineages in our core-genome analysis. This exchange of accessory genetic content may also contribute to the existence of multiple species-level clades within these lineages based on ANI, since any individual strain might harbor partly *X. sacchari* and partly *X. translucens* accessory genes. Second, our gene family frequency distribution revealed that the majority of gene families in *Xanthomonas* were either extremely common (present in all strains) or very rare (present in only a few strains) (Supplementary Figure S8). This pattern is associated with high rates of horizontal acquisition of genes from outside the genus (Dillon et al., 2019). Third, we observe high numbers of both ancestral and recent recombination events in gene families that were present in at least 10% of *Xanthomonas* strains (Figure 8). These results are consistent with prior studies in *Xanthomonas* that have implicated HGT in the exchange of virulence-associated genes between strains (Lima et al., 2008; Merda et al., 2017; Jibrin et al., 2018), in the convergent evolution of phylogenetically distant strains to the same host (Chen et al., 2018), and in shifts in strain tissue-specificity (Gluck-Thaler et al., 2020). However, while some studies have identified lineages of *Xanthomonas* that display evidence of rampant HGT, other lineages appear to be largely clonal (Timilsina et al., 2015, 2019b), and prior estimates of the number of gene families displaying evidence of HGT in *X. citri* and *X. campestris* have been as low as 10% (Huang et al., 2015). Our analysis on an expanded collection of genomes across the *Xanthomonas* genus suggests that most gene families have been horizontally transferred in at least a subset of strains, though some families are clearly recombining more frequently than others (Figure 8).

Several studies have also emphasized that HGT of virulence associated genes can play a major role in their dissemination and ultimately produce new combinations of virulence factors that enable shifts in host specificity (Lima et al., 2008; Merda et al., 2017; Jibrin et al., 2018; Timilsina et al., 2019b). While we find that recombination is common in all gene families, we do not find that it is more common in virulence associated genes than in the non-virulence associated genes (Figure 8). This was surprising given that prior studies in *X. euvesicaotria* (*X. perforans* here) and *P. syringae* have found elevated rates of recombination in virulence associated genes (Dillon et al., 2019; Newberry et al., 2019). While it’s likely that some virulene associated genes do indeed have elevated rates of recombination due to their presence on plasmids and other mobile genetic elements (Kim et al., 1998; Deb et al., 2022), this phenomenon appears to be restricted to a subset of virulence factor families.

### 4.5. Summary

In this study, we explored the pangenome content and evolution of nearly 2,000 *Xanthomonas* strains isolated from 211 locations across the globe that are capable of causing disease on at least 184 plant hosts. We found that *Xanthomonas* has a diverse pangenome, and while our analysis indicates that there is still considerable room for pangenome growth through increased sampling, the relative size of the *Xanthomonas* pangenome is smaller than those of other phytopathogenic taxa. We also used an updated phylogenetic framework based on the core-genome content of our strain collection to establish robust evolutionary relationships between *Xanthomonas* species and illustrate that evolutionarily distinct *Xanthomonas* lineages have frequently converged to cause disease on the same hosts. Finally, we showed an expansion of several classes of virulence factors in the *Xanthomonas* lineage and highlight the mosaic distribution of the majority of T3SE families, which is likely driven by both selection and recombination of these critical virulence factors across strains. Identifying the focal genes that drive adaptative convergence of evolutionary distinct *Xanthomonas* strains to the same hosts and the role of T3SE turnover in *Xanthomonas* host specificity will be critical priorities moving forward, so that we can better protect our vulnerable crops from these diverse and highly virulent pathogens.

## Data availability statement

The datasets presented in this study can be found in online repositories. The names of the repository/repositories and accession number(s) can be found in the article/Supplementary material.

## Author contributions

VA, RS, and MD designed the research. VA, RS, ZN, and MD analyzed and interpreted the data. VA and MD wrote the manuscript. All authors contributed to the article and approved the submitted version.

## Funding

This work was funded by a Natural Sciences and Engineering Research Council of Canada Discovery Award (RGPIN-2021-02701), a Canada Foundation for Innovation John R. Evans Leaders Award (41262), an Ontario Research Fund Award (41262), and a Connaught New Researcher Award (210032) to MD.

## Conflict of interest

The authors declare that the research was conducted in the absence of any commercial or financial relationships that could be construed as a potential conflict of interest.

## Publisher’s note

All claims expressed in this article are solely those of the authors and do not necessarily represent those of their affiliated organizations, or those of the publisher, the editors and the reviewers. Any product that may be evaluated in this article, or claim that may be made by its manufacturer, is not guaranteed or endorsed by the publisher.

## Footnotes

^1^ http://www.biopred.net/xanthomonas
