# Supplementary material for: Pangenome insights into the diversification and disease specificity of worldwide Xanthomonas outbreaks
Source: Front Microbiol. 2023 Jul 5;14:1213261. doi: 10.3389/fmicb.2023.1213261 (PMC10356107; doi:10.3389/fmicb.2023.1213261)
Supplement: Supplementary file 1 [file Data_Sheet_1.PDF]

frontiers

Supplementary Material

Pangenome insights into the diversification and disease specificity of worldwide *Xanthomonas* outbreaks

Viplav Agarwal[1,2], Rachel Stubits[1], Zain Nassrullah[1], and Marcus M. Dillon[1,2*]

[1]Department of Biology, University of Toronto Mississauga, Mississauga, Ontario, Canada

[2]Department of Ecology and Evolutionary Biology, University of Toronto, Toronto, Ontario, Canada

*** Correspondence:**
Marcus M. Dillon
marcus.dillon@utoronto.ca

1 Supplemental Tables

Supplemental Table S1: Representative *hrp* and *hrc* genes used for the identification of type III secretion system (T3SS) pathogenicity islands in this study.

Primary Name	Alternate Name(s)	Annotation	Protein Sequence
HrcC	YscC	Outer membrane ring protein	MAYACPPVHRHRRAPLAAALLLGLLPLLPPHANAASVPWHSRSFKYVADRKDLKEVLRDL<br>SASQSITTWISPEVTGTLSGKFEATPQKFLDDLSGTFGFVWYYDGSVLRIWGANETKNATLS<br>LGAASTSALRDALARMRLDDPRFPVRYDETAHLAVVSGPPGYVDTVAAIAKQVEQVARQ<br>RDATEVQVFQLHYAQAADHTTRIGGQDIQVPGMASLLRNIYGVRGAPTAALPGPGANFGR<br>VQPIGGGSSNTFGNSGQRQSGGSGILGLPASWFGAGSPSERVPVSPPLPGSGNSANAPASVW<br>PEMSQARRDAPLAVDAGSGGELASDAPVIEADPRTNGILIRDRPERMAAYGTLIQQLDNRP<br>KLLQIDATIIEIRDGALQDLGVDWRFHSRRVDVQTGDGRGGQLGYDGSLSGAAAAGAAAP<br>LGGTLTAVLGDAGRYLMTRVSALEQTNKAKIVSTPQVATLDNVEAVMDHKQQAFVRVSG<br>YASADLYNLSAGVSLRVLPSVVPGSPNGQMRLDVRIEDGQLGANTVDGIPVITSSEITTQAF<br>VNEGQSLLIAGYASDTDQTDLNNVPGLSRIPLVGNLFKHRQQSGSRLQRLFLLTPHIVSP
HrcJ	-	Outer membrane ring protein	MRTLRYLVVLLLALLLSGCDQQLYSGLTENDANDMAVLLTAGVDAEKLTPDDGKTWAV<br>NAPHDQVAYALNVLRTHGMPHERHANLGEMFKKDGLISTPTEERVRFIYGVSQQLSQTLS<br>NIDGVIAADVQIVLPNNDPLSASVKPSSAAVFIKFRVGSDLTSLVPSIKTLVMHSVEGLTYEN<br>VSVTLVPGGAESDAQFAASAPPRAWAWPWLVGCALALCVAVAAAALYWWPSANARRW<br>GGWQRLRALSRKHAG
HrcN	-	ATPase	MLAEMPLLQTTLERELAALAFGRRYGKVVEVIGTMLKVAGVQVSLGEVCELRQRDGTLL<br>QRAEVVGFSRTLALLAPFGELVGLSRQTRVIGLGRPLAVPVGSALLGRVLDGLGEPADGQG<br>PLAGDDWVQIQAQAPDPMRRRLIEQPLPTGVRIVDGLMTLGEGQRMGIFAAAGVGKSTLIG<br>MFARGTQCDVNVIVLIGERGREVREFIEMILGPDGLARSVVVCATSDRSSIERAKAAYVGT<br>AIAEYFRDRGMRVLLMMDSLTRFARAQREIGLAAGEPPTRRGFPPSVFAELPRLLERAGMG<br>ETGSITAFYTVLAEDDTGSDPIAEEVRGILDGHLILSREIAARNQYPAIDVLGSLSRVMSQIVS<br>AEQRQYAGQLRRLLAKHNEVETLLQVGEYRHGSDAVADEAIARIDAIRDFLSQPTDQLSDY<br>DTILEQLAGVIDDA
HrcQ	-	-	MLSTEPSLTSPARDLSQALTHVPAVRAQLGRVLCDPRAAQRCGYTAQRRGIRAADAARLQ<br>LQFDSGSLELRIAARDGLALLLNEADDALRVAIAGVLLSDDLRALEPLGLGAAEVVAFERC<br>ADAVDRLDIGITLGGIDAIAETASPLLLAALQTASAALAQPSPLPAWLSALRVTTRLRIGQRT<br>ATAALLQSLRPGDVLLHALATAPVRSGELLWGIPGGAVLRAPVRLTLQQMILETAPTMQH<br>DMPASDSSSSATDVAALELPVQLEVDQLALSLSVLSGLQPGQILELSVPVDQADIRLVVYG<br>QTIGIGRLLAVGEHLGVQILSMSETAHADA
HrcR	-	Export apparatus subunit	MQMPDVGSLLLVVIMLGLLPFAAMVVTSYTKIVVVLGLLRNAIGVQQVPPNMVLNGVALL<br>VSCFVMAPVGMEAFKAAQNYSPGADNSRVVVLLDACREPFRQFLLKHTREREKAFFIRSA<br>QQIWPKDKADTLKPDDLLVLAPAFTLSELTEAFRIGFLLYLVFIVIDLVVANALMAMGLSQ<br>VTPTNVAIPFKLLLFVALDGWSMLIHGLVLSYR
HrcS	-	-	MDHDDLVRFTSEALLLCLKVSLPVVGVAAVAGLLIAFIQAVMSLQDASISFALKLVVVVAA<br>IAVTAPWGASAIMQFGQALMQAAFP

HrcT	HrpB8	Export apparatus subunit	MSDTATALLALSSQGVSLLTLLALCGVRVFVLFFVLPATAQDSLPGMTRNGVIYVLSSFIAYGQPADALARIEAAGLVGLVFKEAFIGLLIGFAASTVFWVAESVGLLIDDVSGYNNVQMINPLSGEQSTPVSTVLMQLAIVSFYALGGMLMLLGALFESFRWWPLSQLMPDMGAIGESFVIQQTDGMMAAIVKLSAPVMLVLVLVDLAIGFVARAADKLDPSNLSQPIRGVLALLLLALLTSVFIAQSGDALGFLHFQQQLHDAANASAKGGASH
HrcU	-	Export apparatus subunit	MSDEKTEKPTEKKLQDARRDGEVPISPDVTAAAVLLAALLVMKLAGSYFVEHLRALMSIGFDFTTNTRDATALHRALGRIGIQGVLLTLPFVTACLAAGLIGTFVQTGLNASLKPVTPKFDSLNPVNGVKKLFSLRSLINLLKLGIKAAVIGVVLWYGLRALMPTIIGLAYQPPADIAQIGWRALGILCALAVLVFVLVGAADWSVQHWLFIRDKRMSKDEQKREHKESEGDPEVKGKRKEFAKELVFGDPRERVAKAKVMVVNPTHYAVALAYEPDGFGLPQVVAKGVDEGALELRAYAHNQGIPIVANPPLARALHEVELGEAVPESLFETVAVVLRWVDELGRDNDEGSGPLPC
HrcV	FHIPEP	-	MRVTRYFAYTGEVAIAALVVAVIGLMILPLPTPLIDTLLGINITLSVVLLMVTMYVPDSISLSSFPSLLLFTTLLRLSLNIASTKSILLHAEAGHIIESFGELVVGGNLVVGLVVFLIITTVQFIVIAKGSERVAEVGARFTLDAMPGKQMSIDADLRGGNLTADEARRKRARLAIESQLHGGMDGAMKFVKGDAIAGLVITMVNILAGIVVGVTYHGMSAGEAANRFAILSVGDAMVSQIASLLISVAAGVMITRVANENETKISSLGLDIGRQLTSNARALMAASVLLACFAFVPGFPALLFLLLAAAVGAGGYTIWRKQRDTSGSDQPALPSTSRKGAKGDAPHIRKSAPDFASPLSMRLSPQLAARLDPALLDQAIESERRQLVELLGLPFPGIAIWQSESLQGLQYEVLIHDVPETRSALSDTADMQKALAQQAIAPLHARAHLFVGIQETQWMLEQVGADYPGLVAEVNKAMPAQRIADVLRRLLEERIPVRNIKSILESLVVWGPKEKDLLMLTEYVRCDLGRYLAHTATAGTGQLPAVMLDHAVEQLIRQSIRATPAGNFLALPPEQANQLVEQVERIVEDQARHPLAVVASMDVRRYVRRMIEARLNWLEVYSFQELGAEVQLQPIGRVVA
HrpB1	-	-	MEKIECPGSVVSGLIELITVGLTHEKIEEAAAVLAAVRVLRPELKALDTFDAWIAIKRGSYLEGARLLRELEADAGSEPLCKALYACCLFAVGDPSWHGIAEGLIEEDADADAVGLVKALSGRYTPAPAPLEAAAESAAPMDVPNAQYLRA
HrpD5	HPr kinase, HrpQ	Kinase	MTMQLRVLTGIHAGARLDLQPGSYTLGADPQAEIRIEDWPDCSLIIEVDADGQVCYRSEALPTTAFVALHPVRFGPLVLCMGDAAADWPDDVALLEQLLSPAATPAAPSPRRSRRTALRAVVGAMLALAAAALLPSLLPAFLSDAAPPRSQDNQLNQVRFVLKRLGLREARVEQVGSRVRVEGLVTSSADAARLRAQLHRDQHAVTDVVVVDEVLATLRDTLADRDLSVRYDGQGVFSIAGSSDNAERATRRIADLRSDLGPEIRTLHVEITQQDPSVKPPANYDAALLADGLHYVETPDGTKHLTSLPQQAAP
HrpF	-	Serine kinase	MSLNTLSTGSTAGLFLPLTDDASSPGLLGSDSAMNDSDLLLAMDNLFLQQIYRLIAATYGNTSLNGPGSGIPGLDTPSADDLQASQPIEKRTSWPTLSAPFNVKDIKGSRLPPAVDGSSVTWEGGTLTPSELQIVSTLNQHKDKTPLEFAKLDDKINDPSTPPDLKSALQGLQKDPRLFFAIGSQGDGKCGGKIKAGDLWDFADHHQQVTALGGKNAEFNPKNIKGATPPPAAEGSSVTWDGGTLTQSQLEIVSTLNQHRDMMPIEFAKLDEKINDPATPPDLKKALQGLQQDPGLFFAMASQGHGKHHHDDQGKCNGKLIADNLYDFADRHPQVTAQGGKNATYNPEKMKGRDLPPPVDGSSVTWDGGTLTQNELEIVATLNRHKDKCPVKWTDLDAKSKDPAIPPDLQKAFADLQQDPALFHAIGAQGSKGSCDGKFTEKDLTRFSVPEKHAQIAQYAEQQAKGYTQNYVASDSPDKTEPTVMTESDAMRELYRYSDYLPKDLNQDAFKQLVEGDSTTKKSPPQVIAAAQYFREHPDQWKALAGDKESMSTADFLQKSTSEMHLTAPELKTLDTINSHQEAFFGDGKEVTRDKLDTIVKDDKADPAVRDAAKQLLGDPLLFGLLNNAITGYKKPHSFFGGGHVVDSGKISNKDFQQFYEHMTAVNKTLDTPPTHAATSPEQKKAVADMLMGKADQPEIKRKKHDVGTFSKGLHEFLKWDSKILDGISVALSAMNGIPLIGEVADAAALAFESEAQAAQVIDTALQGGNLSLAWKLAGINMAGAVVGAVGGPTARLAAKGAAKGVAEGAAKGATQGTTKGAAKGGGKGVAEREKPLDIAKGYIIGTSINRPTEMLKTPVLAGLHYEEVRLDKEKKKGEIRKNLEAAGGVPLGKQFIPKAIADNFEADTKENLRHVRGRRK

2 Supplemental Figures

See attached PDF (FigureS1.pdf).

Supplemental Figure S1: Rarefaction curves for the core and accessory genome of each individual *Xanthomonas* species characterized in this study. For the core genome curves, families present in 95% of strains are considered part of the soft-core genome (upper curve) and families present in 100% of strains are considered part of the hard-core genome (lower curve). For the soft-core genome curves, the analysis was performed both with (upper curve) and without (lower curve) gene families that were only present in a single strain.

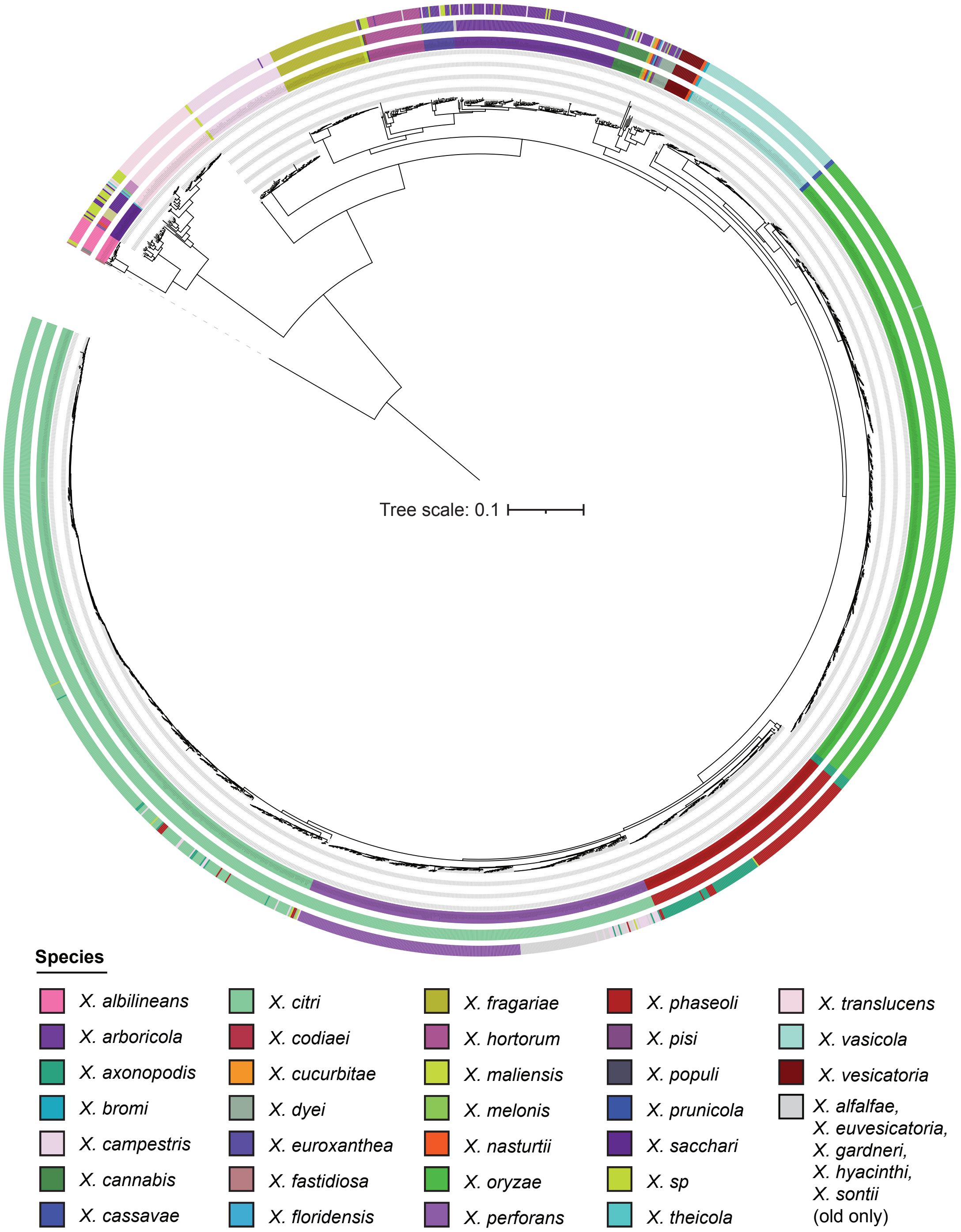

Supplemental Figure S2: Core genome phylogenetic tree of the 1,910 *Xanthomonas* strains analyzed in this study, illustrating updated species designations based on the phylogenetic relationships of the strains. The phylogenetic tree was generated from a concatenated core-genome amino acid alignment of the 1,910 *Xanthomonas* strains and *Xyella fastidiosa* 9a5c using FastTree. The inner colour ring illustrates monophyletic species designations of strains as they are defined in this study, the middle colour ring illustrates species clusters as they are defined by ANI at a threshold of 95%, and the outer colour ring illustrates species designations as they were initially defined on NCBI. Species that did not form monophyletic groups based on the core genome alignment were reassigned as described in the Methods.

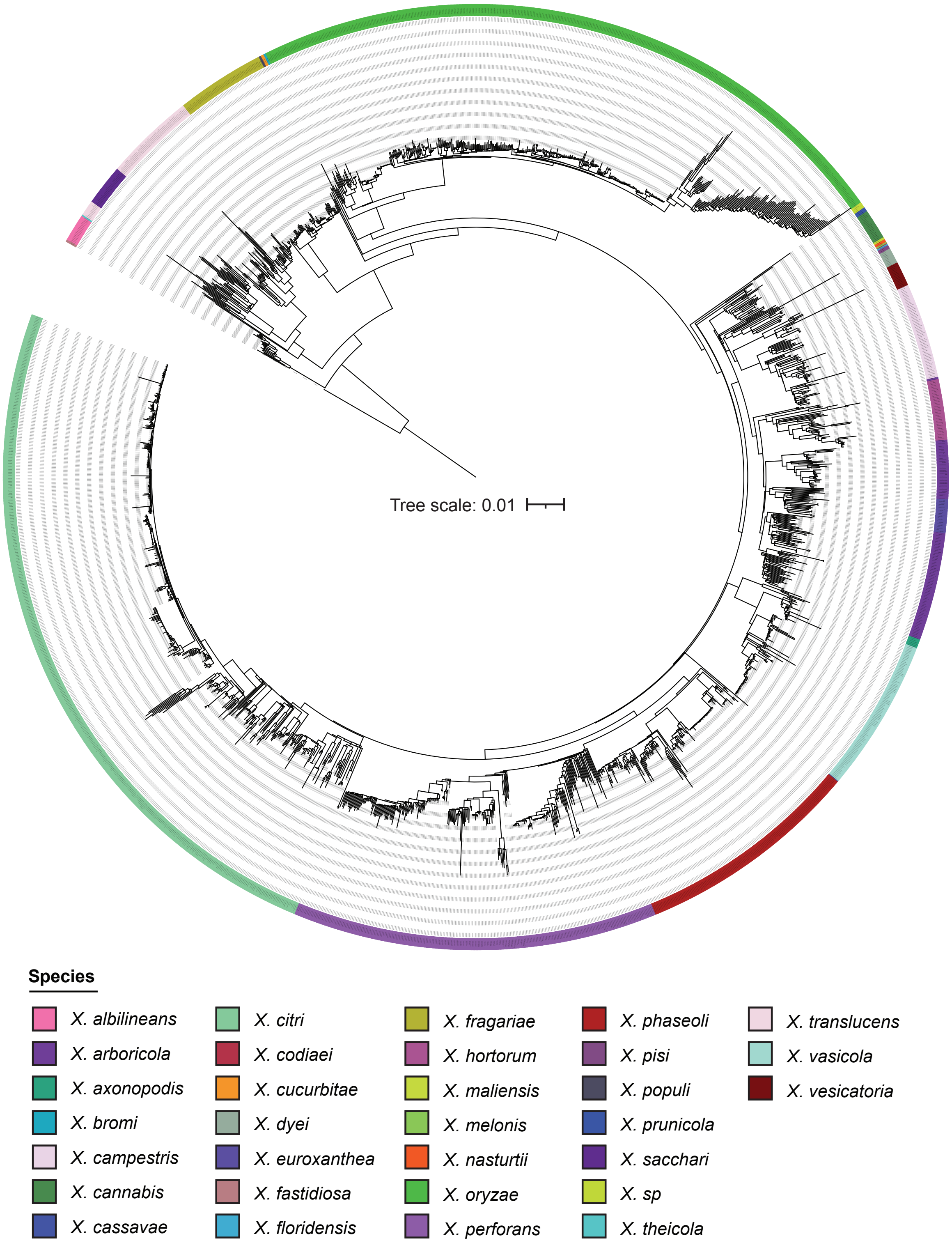

Supplemental Figure S3: Evolutionary relationships between the 1,910 *Xanthomonas* strains analyzed in this study based on their pan-genome content. The phylogenetic tree was generated using the presence-absence matrix of all gene families in our collection as input for FastTree. The tree is rooted on *X. fastidiosa* 9a5c and the surrounding colour ring illustrates species designations of the strains based on the core-genome phylogenetic tree.

See attached PDF (FigureS4.pdf).

Supplemental Figure S4: Core genome phylogenetic trees for each of the *Xanthomonas* species analyzed in this study that contained more than one strain. Phylogenetic trees were generated from a concatenated core-genome amino acid alignment of all strains from each species using FastTree and were rooted at the midpoint. Colour bands illustrate the host of isolation for each strain, if available.

See attached PDF (FigureS5.pdf).

Supplemental Figure S5: Distribution of virulence associated gene categories from the VFDB in each of the *Xanthomonas* species analyzed in this study. Bars represent the average number of virulence associated genes in the corresponding category across all strains from each species. Error bars represent the standard error of the mean across strains from each species.

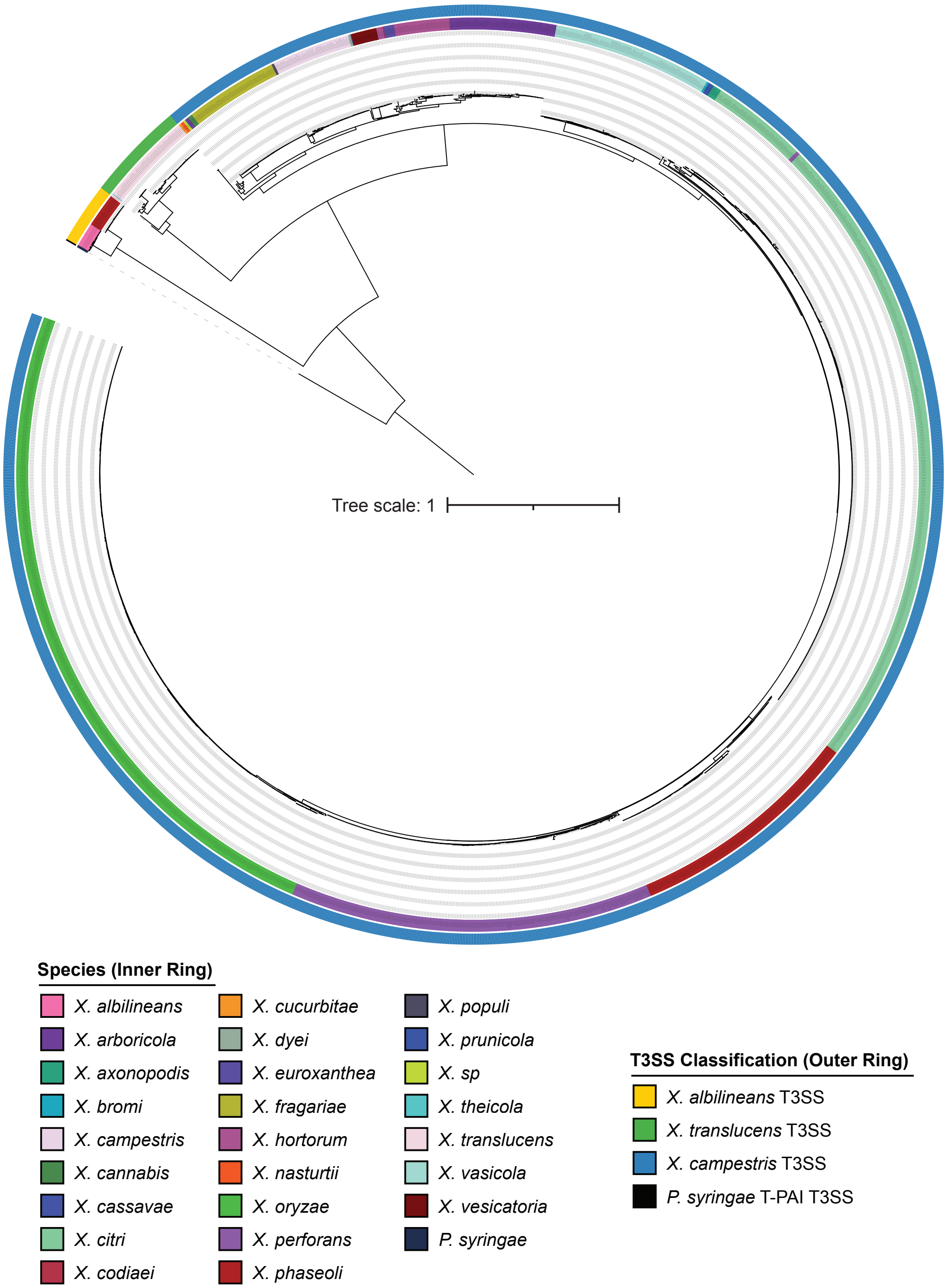

Supplemental Figure S6: Evolutionary relationships between the three categories of *Xanthomonas* type III secretions systems (T3SSs) classified in this study. The phylogenetic tree was generated with FastTree using a concatenated alignment of twelve core genes from each T3SS and was rooted on the T-PAI T3SS from *Pseudomonas syringae* PtoDC3000. The inner colour ring illustrates species designations of the corresponding strains, while the outer ring illustrates the assigned T3SS based on their phylogenetic relationships. Strains of *X. phaseoli* that have two T3SSs appear twice on the tree.

See attached PDF (FigureS7.pdf).

Supplemental Figure S7: Phylogenetic distribution of the three categories of *Xanthomonas* type III secretion systems (T3SSs) classified in this study across the core-genome phylogeny of 1,910 *Xanthomonas* strains. The phylogenetic tree was generated from a concatenated core-genome amino acid alignment of the 1,910 *Xanthomonas* strains and *Xyella fastidiosa* 9a5c using FastTree. Filled circles indicate that a particular T3SS is present in the strain, while empty circles indicate that the T3SS is absent.

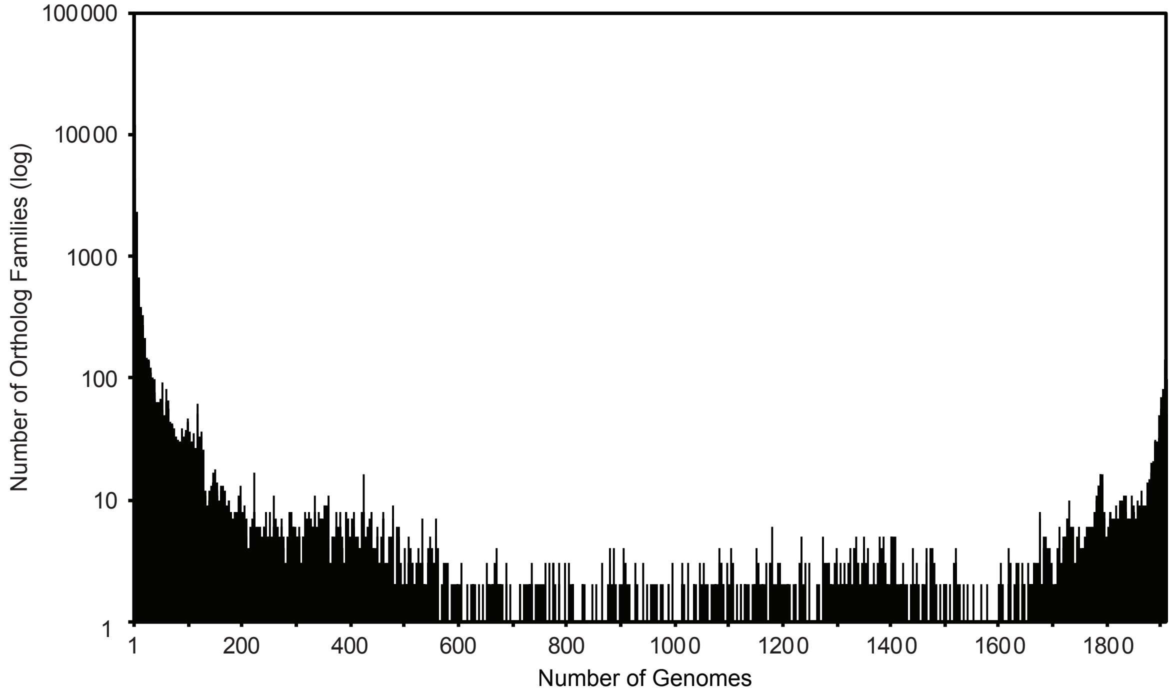

Supplemental Figure S8: Gene family frequency distribution for each *Xanthomonas* ortholog family classified in this study. The tendency for ortholog families to be identified in either few or most genomes suggests that horizonal gene transfer (HGT) is common in the *Xanthomonas* genus.

3 Supplemental Datasets

Supplemental Dataset S1: Complete metadata and assembly information for all 1,910 *Xanthomonas* genomes used in this study.

See attached XLSX (DatasetS1.xlsx).

Supplemental Dataset S2: Summary of T3SE repertoires for all 1,911 *Xanthomonas* genomes analyzed in this study. Data is presented as the total copies of each T3SE family in each strain (tab 1) and as a presence-absence matrix for at least one copy of each T3SE family in each strain (tab 2).

See attached XLSX (DatasetS2.xlsx).

4 Supplemental Scripts

Supplemental Script S1: R script used to generate genus-wide rarefaction curve across all *Xanthomonas* species.

See attached file (ScriptS1.r)

Supplemental Script S2: R script used to generate within species rarefaction curves.

See attached file (ScriptS2.r)

Supplemental Script S3: Python script used to translate core-genome alignment obtained from PIRATE.

See attached file (ScriptS3.py)
