# Supplementary material for: Pangenome insights into the diversification and disease specificity of worldwide Xanthomonas outbreaks
Source: Front Microbiol. 2023 Jul 5;14:1213261. doi: 10.3389/fmicb.2023.1213261 (PMC10356107; doi:10.3389/fmicb.2023.1213261)
Supplement: Supplementary file 3 [file Image_1.PDF]

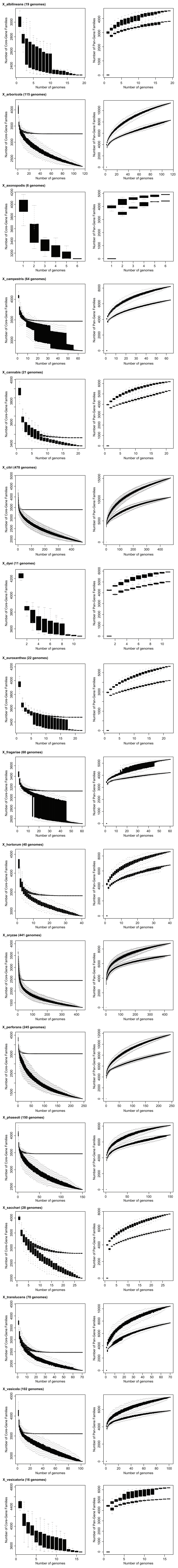
X_albilineans (19 genomes)
X_arboricola (115 genomes)
X_axonopodis (6 genomes)
X_campestris (64 genomes)
X_cannabis (21 genomes)
X_citri (478 genomes)
X_dyei (11 genomes)
X_euroxanthea (22 genomes)
X_fragariae (60 genomes)
X_hortorum (40 genomes)
X_oryzae (441 genomes)
X_perforans (245 genomes)
X_phaseoli (150 genomes)
X_sacchari (28 genomes)
X_translucens (70 genomes)
X_vasicola (102 genomes)
X_vesicatoria (16 genomes)
Number of Core-Gene Families
Number of Pan-Gene Families
Number of genomes
