# Supplementary material for: Pangenome insights into the diversification and disease specificity of worldwide Xanthomonas outbreaks
Source: Front Microbiol. 2023 Jul 5;14:1213261. doi: 10.3389/fmicb.2023.1213261 (PMC10356107; doi:10.3389/fmicb.2023.1213261)
Supplement: Supplementary file 5 [file Image_3.PDF]

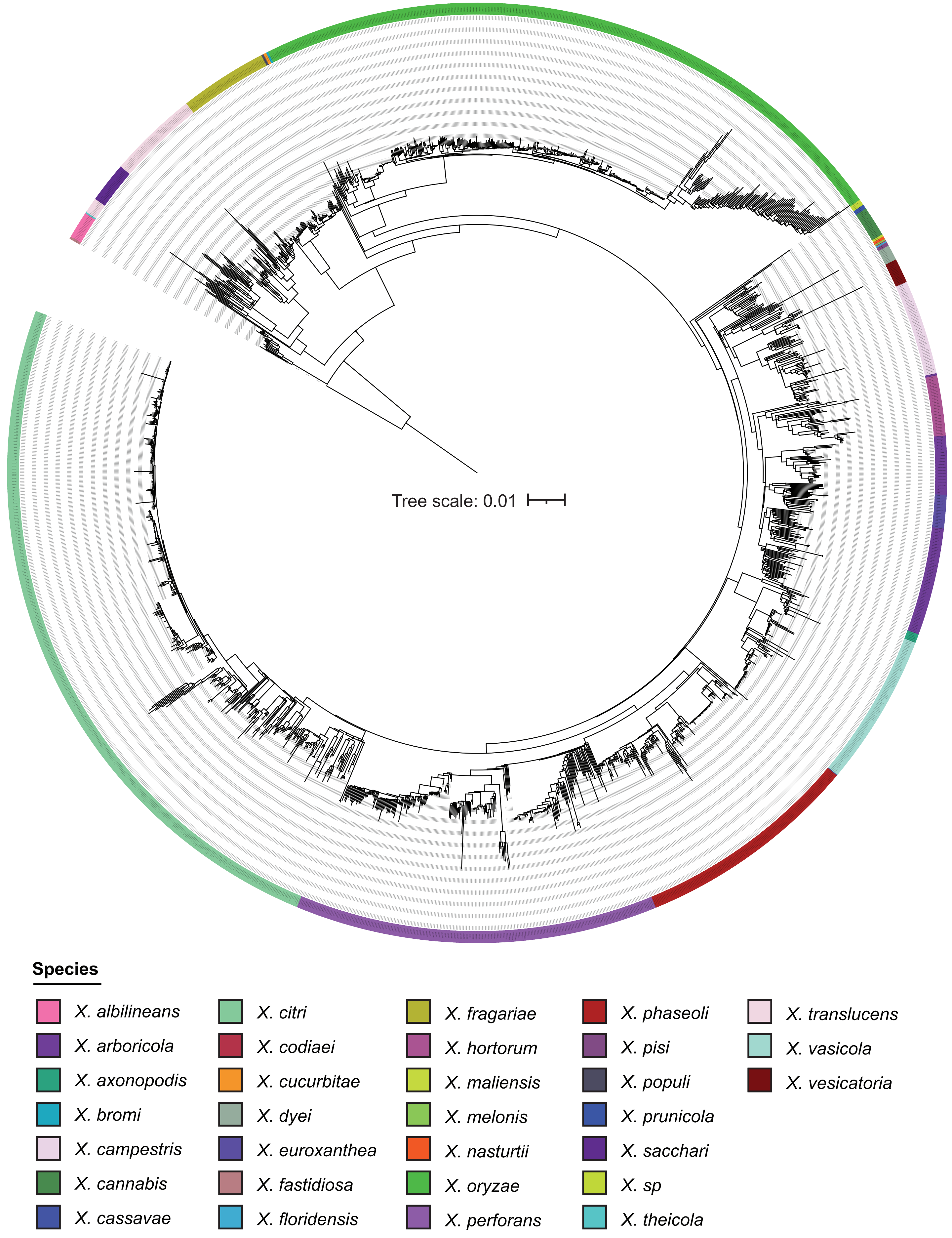

Tree scale: 0.01
Species
X. albilineans
X. arboricola
X. axonopodis
X. bromi
X. campestris
X. cannabis
X. cassavae
X. citri
X. codiaei
X. cucurbitae
X. dyei
X. euroxanthea
X. fastidiosa
X. floridensis
X. fragariae
X. hortorum
X. maliensis
X. melonis
X. nasturtii
X. oryzae
X. perforans
X. phaseoli
X. pisi
X. populi
X. prunicola
X. sacchari
X. sp
X. theicola
X. translucens
X. vasicola
X. vesicatoria
