# Supplementary material for: Pangenome insights into the diversification and disease specificity of worldwide Xanthomonas outbreaks
Source: Front Microbiol. 2023 Jul 5;14:1213261. doi: 10.3389/fmicb.2023.1213261 (PMC10356107; doi:10.3389/fmicb.2023.1213261)
Supplement: Supplementary file 6 [file Image_4.PDF]

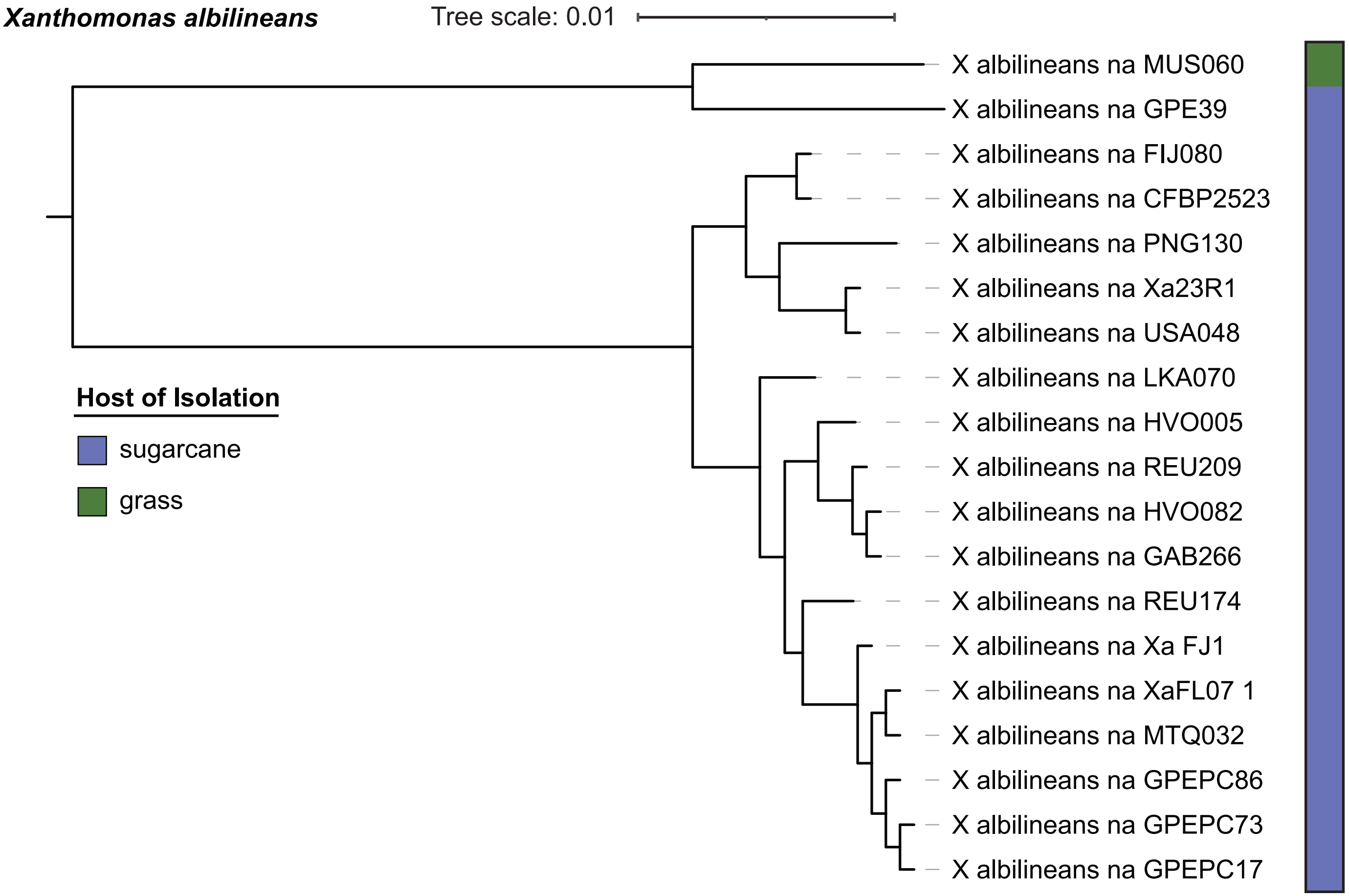

Xanthomonas albilineans
Tree scale: 0.01
X albilineans na MUS060
X albilineans na GPE39
X albilineans na FIJ080
X albilineans na CFBP2523
X albilineans na PNG130
X albilineans na Xa23R1
X albilineans na USA048
X albilineans na LKA070
X albilineans na HVO005
X albilineans na REU209
X albilineans na HVO082
X albilineans na GAB266
X albilineans na REU174
X albilineans na Xa FJ1
X albilineans na XaFL07 1
X albilineans na MTQ032
X albilineans na GPEPC86
X albilineans na GPEPC73
X albilineans na GPEPC17
Host of Isolation
sugarcane
grass

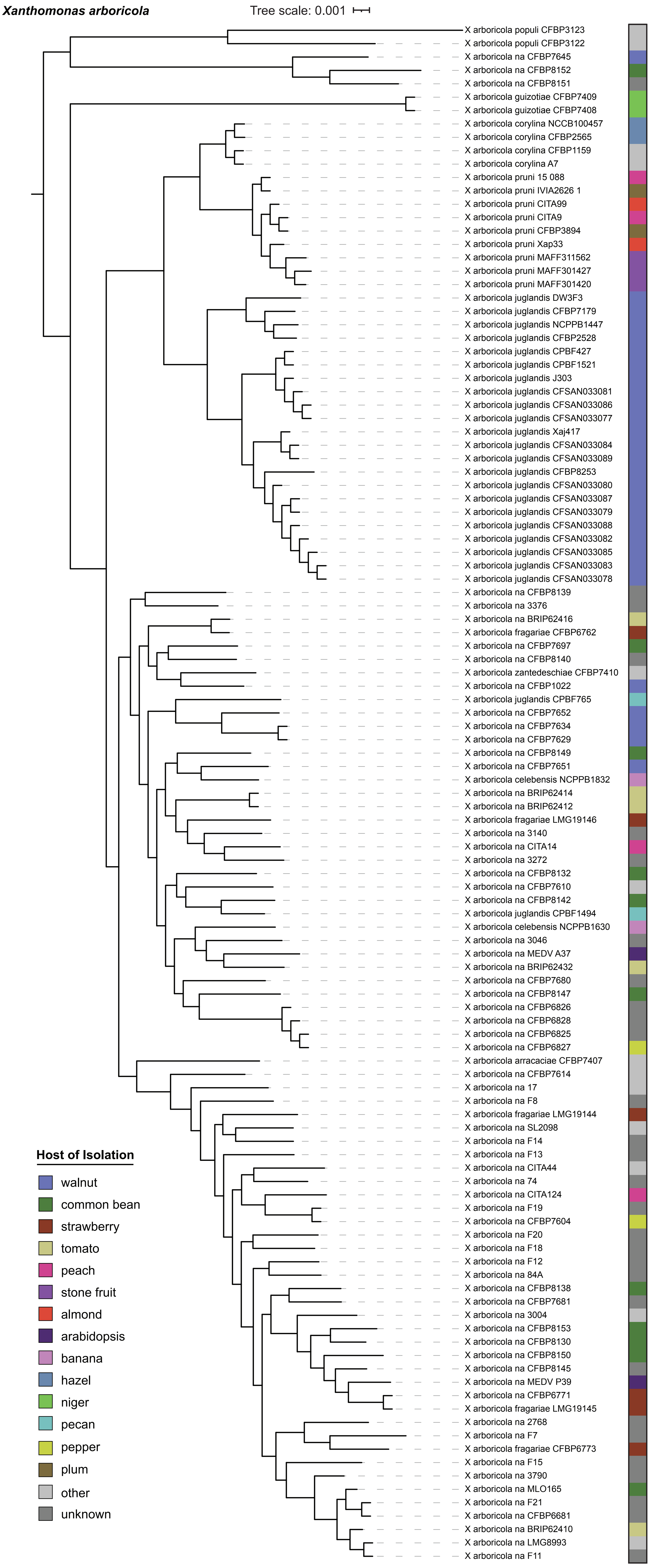

Xanthomonas arboricola
Tree scale: 0.001
X arboricola populi CFBP3123
X arboricola populi CFBP3122
X arboricola na CFBP7645
X arboricola na CFBP8152
X arboricola na CFBP8151
X arboricola guizotiae CFBP7409
X arboricola guizotiae CFBP7408
X arboricola corylina NCCB100457
X arboricola corylina CFBP2565
X arboricola corylina CFBP1159
X arboricola corylina A7
X arboricola pruni 15 088
X arboricola pruni IVIA2626 1
X arboricola pruni CITA99
X arboricola pruni CITA9
X arboricola pruni CFBP3894
X arboricola pruni Xap33
X arboricola pruni MAFF311562
X arboricola pruni MAFF301427
X arboricola pruni MAFF301420
X arboricola juglandis DW3F3
X arboricola juglandis CFBP7179
X arboricola juglandis NCPPB1447
X arboricola juglandis CFBP2528
X arboricola juglandis CPBF427
X arboricola juglandis CPBF1521
X arboricola juglandis J303
X arboricola juglandis CFSAN033081
X arboricola juglandis CFSAN033086
X arboricola juglandis CFSAN033077
X arboricola juglandis Xaj417
X arboricola juglandis CFSAN033084
X arboricola juglandis CFSAN033089
X arboricola juglandis CFBP8253
X arboricola juglandis CFSAN033080
X arboricola juglandis CFSAN033087
X arboricola juglandis CFSAN033079
X arboricola juglandis CFSAN033088
X arboricola juglandis CFSAN033082
X arboricola juglandis CFSAN033085
X arboricola juglandis CFSAN033083
X arboricola juglandis CFSAN033078
X arboricola na CFBP8139
X arboricola na 3376
X arboricola na BRIP62416
X arboricola fragariae CFBP6762
X arboricola na CFBP7697
X arboricola na CFBP8140
X arboricola zantedeschiae CFBP7410
X arboricola na CFBP1022
X arboricola juglandis CPBF765
X arboricola na CFBP7652
X arboricola na CFBP7634
X arboricola na CFBP7629
X arboricola na CFBP8149
X arboricola na CFBP7651
X arboricola celebensis NCPPB1832
X arboricola na BRIP62414
X arboricola na BRIP62412
X arboricola fragariae LMG19146
X arboricola na 3140
X arboricola na CITA14
X arboricola na 3272
X arboricola na CFBP8132
X arboricola na CFBP7610
X arboricola na CFBP8142
X arboricola juglandis CPBF1494
X arboricola celebensis NCPPB1630
X arboricola na 3046
X arboricola na MEDV A37
X arboricola na BRIP62432
X arboricola na CFBP7680
X arboricola na CFBP8147
X arboricola na CFBP6826
X arboricola na CFBP6828
X arboricola na CFBP6825
X arboricola na CFBP6827
X arboricola arracaciae CFBP7407
X arboricola na CFBP7614
X arboricola na 17
X arboricola na F8
X arboricola fragariae LMG19144
X arboricola na SL2098
X arboricola na F14
X arboricola na F13
X arboricola na CITA44
X arboricola na 74
X arboricola na CITA124
X arboricola na F19
X arboricola na CFBP7604
X arboricola na F20
X arboricola na F18
X arboricola na F12
X arboricola na 84A
X arboricola na CFBP8138
X arboricola na CFBP7681
X arboricola na 3004
X arboricola na CFBP8153
X arboricola na CFBP8130
X arboricola na CFBP8150
X arboricola na CFBP8145
X arboricola na MEDV P39
X arboricola na CFBP6771
X arboricola fragariae LMG19145
X arboricola na 2768
X arboricola na F7
X arboricola fragariae CFBP6773
X arboricola na F15
X arboricola na 3790
X arboricola na MLO165
X arboricola na F21
X arboricola na CFBP6681
X arboricola na BRIP62410
X arboricola na LMG8993
X arboricola na F11
Host of Isolation
walnut
common bean
strawberry
tomato
peach
stone fruit
almond
arabidopsis
banana
hazel
niger
pecan
pepper
plum
other
unknown

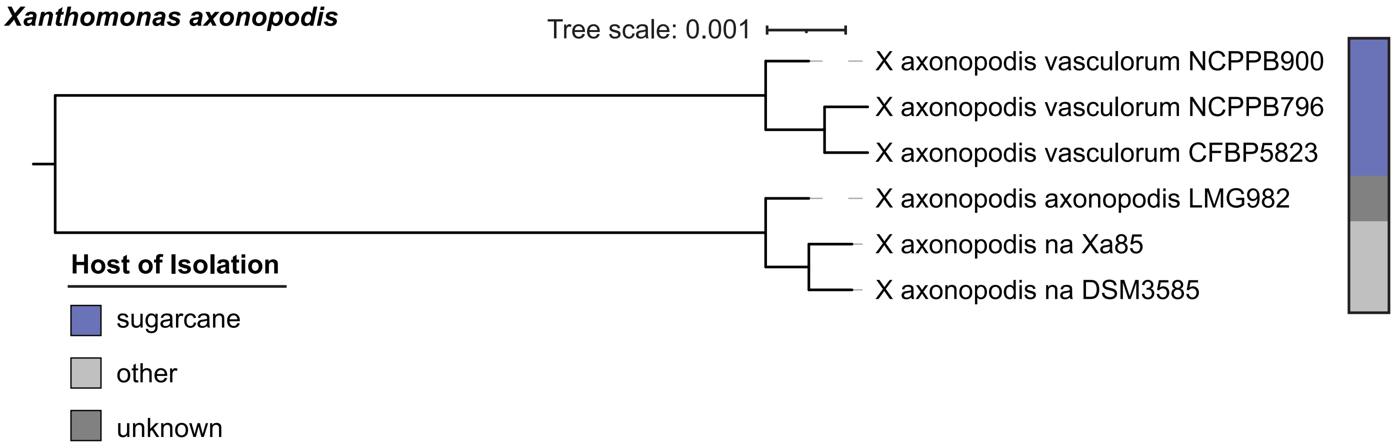
Xanthomonas axonopodis
Tree scale: 0.001
X axonopodis vasculorum NCPPB900
X axonopodis vasculorum NCPPB796
X axonopodis vasculorum CFBP5823
X axonopodis axonopodis LMG982
X axonopodis na Xa85
X axonopodis na DSM3585
Host of Isolation
sugarcane
other
unknown

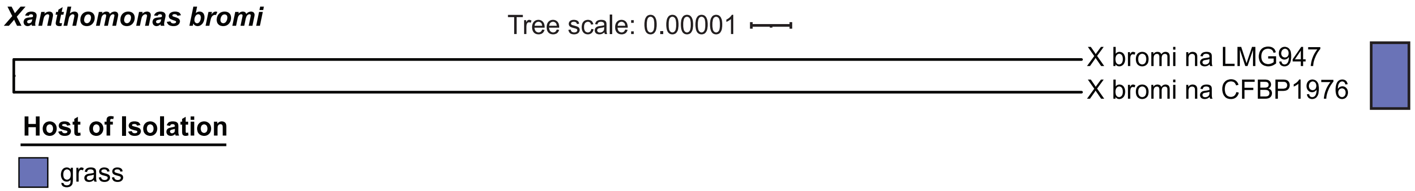

Xanthomonas bromi
Tree scale: 0.00001
X bromi na LMG947
X bromi na CFBP1976
Host of Isolation
grass

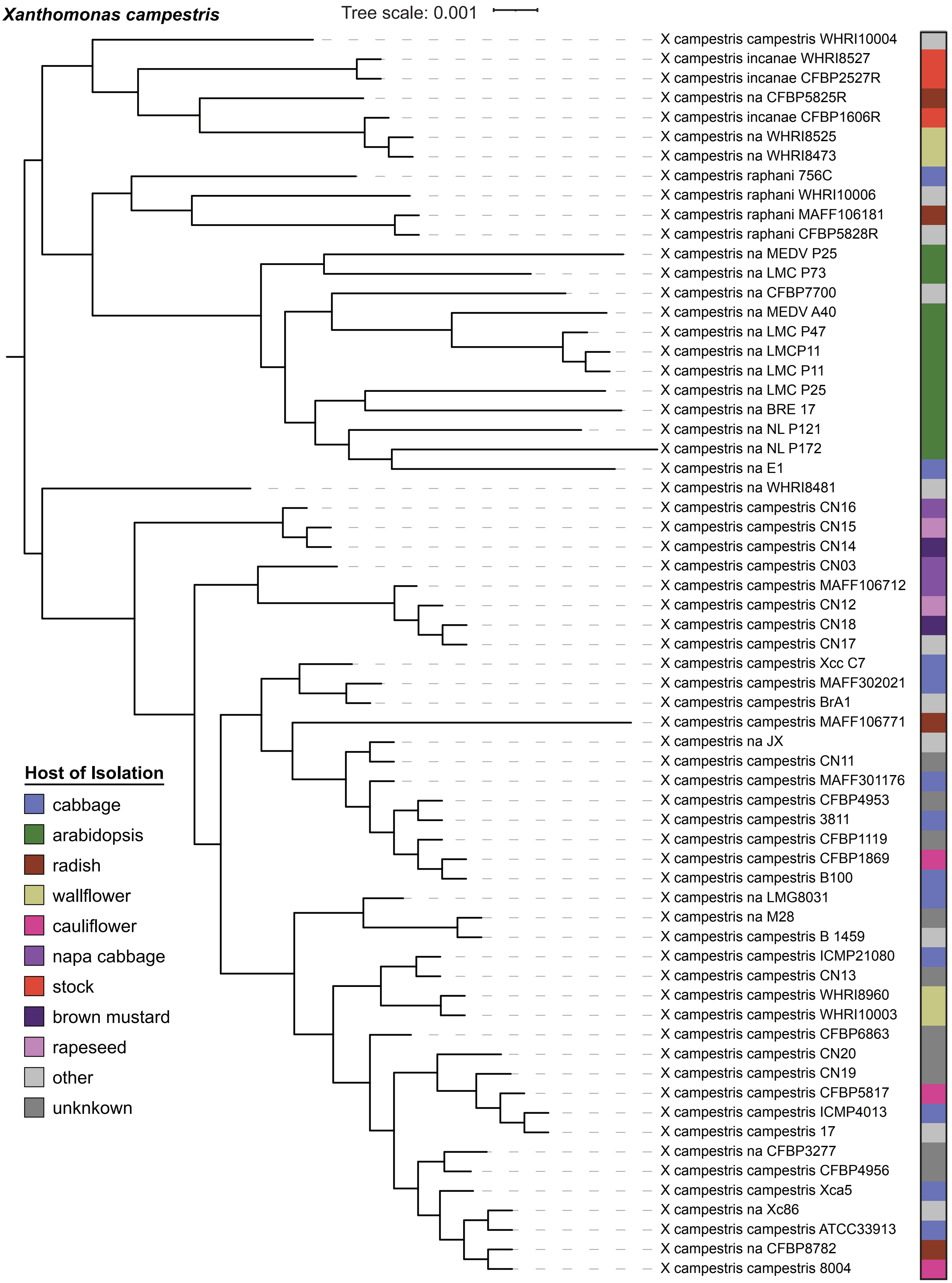
Xanthomonas campestris
Tree scale: 0.001
X campestris campestris WHRI10004
X campestris incanae WHRI8527
X campestris incanae CFBP2527R
X campestris na CFBP5825R
X campestris incanae CFBP1606R
X campestris na WHRI8525
X campestris na WHRI8473
X campestris raphani 756C
X campestris raphani WHRI10006
X campestris raphani MAFF106181
X campestris raphani CFBP5828R
X campestris na MEDV P25
X campestris na LMC P73
X campestris na CFBP7700
X campestris na MEDV A40
X campestris na LMC P47
X campestris na LMCP11
X campestris na LMC P11
X campestris na LMC P25
X campestris na BRE 17
X campestris na NL P121
X campestris na NL P172
X campestris na E1
X campestris na WHRI8481
X campestris campestris CN16
X campestris campestris CN15
X campestris campestris CN14
X campestris campestris CN03
X campestris campestris MAFF106712
X campestris campestris CN12
X campestris campestris CN18
X campestris campestris CN17
X campestris campestris Xcc C7
X campestris campestris MAFF302021
X campestris campestris BrA1
X campestris campestris MAFF106771
X campestris na JX
X campestris campestris CN11
X campestris campestris MAFF301176
X campestris campestris CFBP4953
X campestris campestris 3811
X campestris campestris CFBP1119
X campestris campestris CFBP1869
X campestris campestris B100
X campestris na LMG8031
X campestris na M28
X campestris campestris B 1459
X campestris campestris ICMP21080
X campestris campestris CN13
X campestris campestris WHRI8960
X campestris campestris WHRI10003
X campestris campestris CFBP6863
X campestris campestris CN20
X campestris campestris CN19
X campestris campestris CFBP5817
X campestris campestris ICMP4013
X campestris campestris 17
X campestris na CFBP3277
X campestris campestris CFBP4956
X campestris campestris Xca5
X campestris na Xc86
X campestris campestris ATCC33913
X campestris na CFBP8782
X campestris campestris 8004
Host of Isolation
cabbage
arabidopsis
radish
wallflower
cauliflower
napa cabbage
stock
brown mustard
rapeseed
other
unknkown

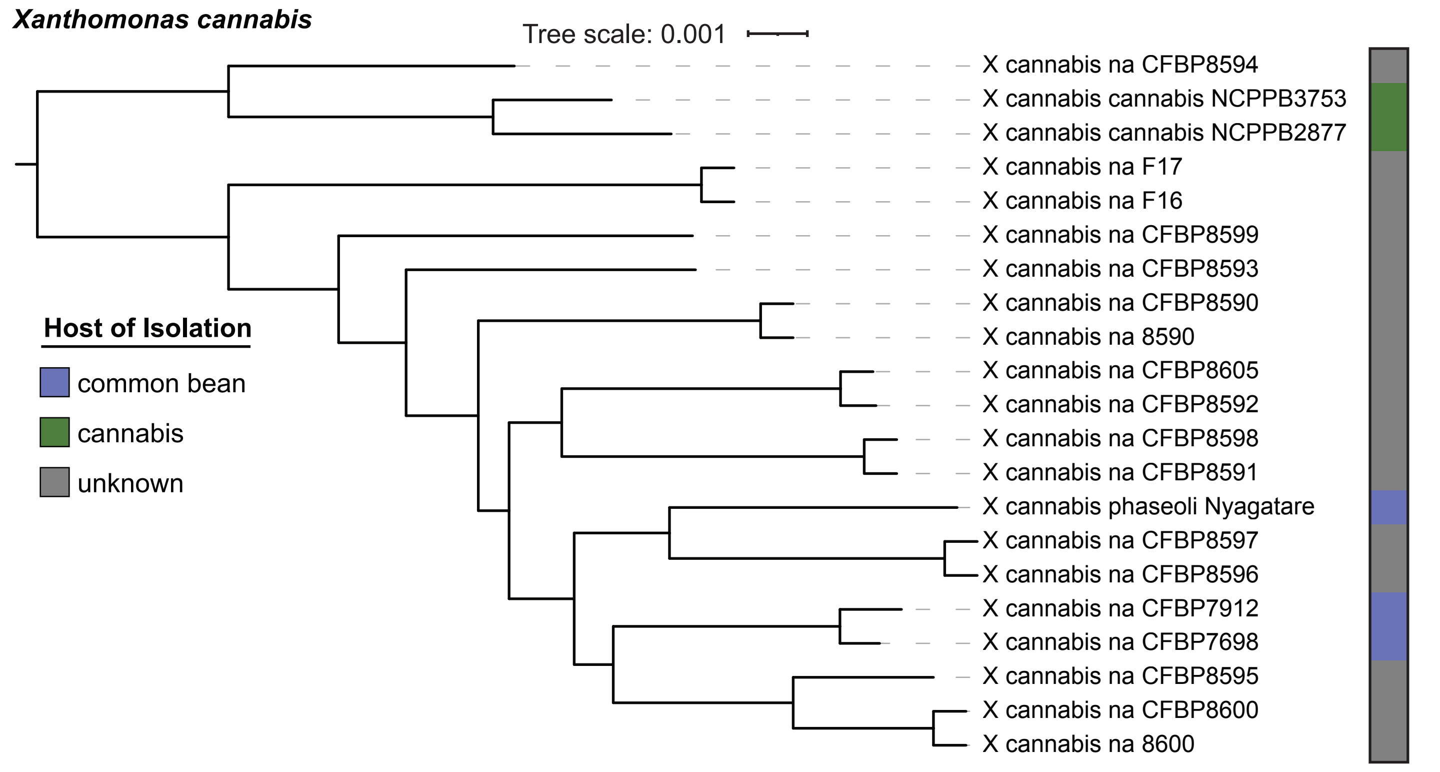
Xanthomonas cannabis
Tree scale: 0.001
X cannabis na CFBP8594
X cannabis cannabis NCPPB3753
X cannabis cannabis NCPPB2877
X cannabis na F17
X cannabis na F16
X cannabis na CFBP8599
X cannabis na CFBP8593
X cannabis na CFBP8590
X cannabis na 8590
X cannabis na CFBP8605
X cannabis na CFBP8592
X cannabis na CFBP8598
X cannabis na CFBP8591
X cannabis phaseoli Nyagatare
X cannabis na CFBP8597
X cannabis na CFBP8596
X cannabis na CFBP7912
X cannabis na CFBP7698
X cannabis na CFBP8595
X cannabis na CFBP8600
X cannabis na 8600
Host of Isolation
common bean
cannabis
unknown

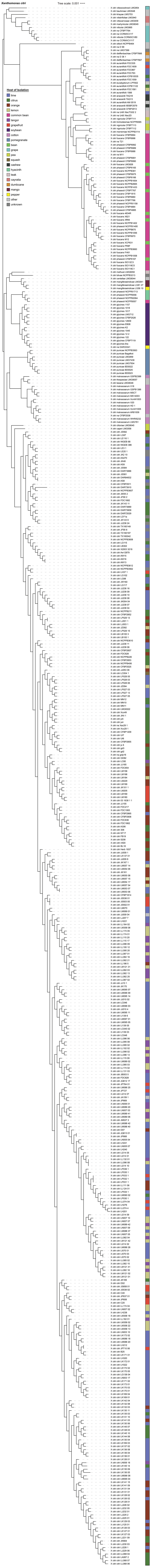

Xanthomonas citri
Tree scale: 0.001
Host of Isolation
lime
citrus
orange
lemon
common bean
tangor
grapefruit
soybean
cotton
pomegranate
bean
grape
pea
squash
cashew
hyacinth
teak
cayratia
dumbcane
mango
pepper
other
unknown

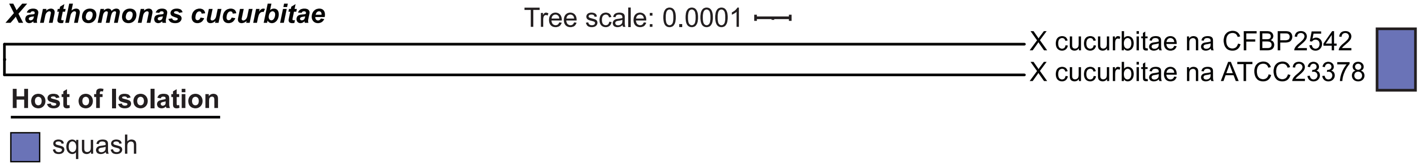
Xanthomonas cucurbitae
Tree scale: 0.0001
X cucurbitae na CFBP2542
X cucurbitae na ATCC23378
Host of Isolation
squash

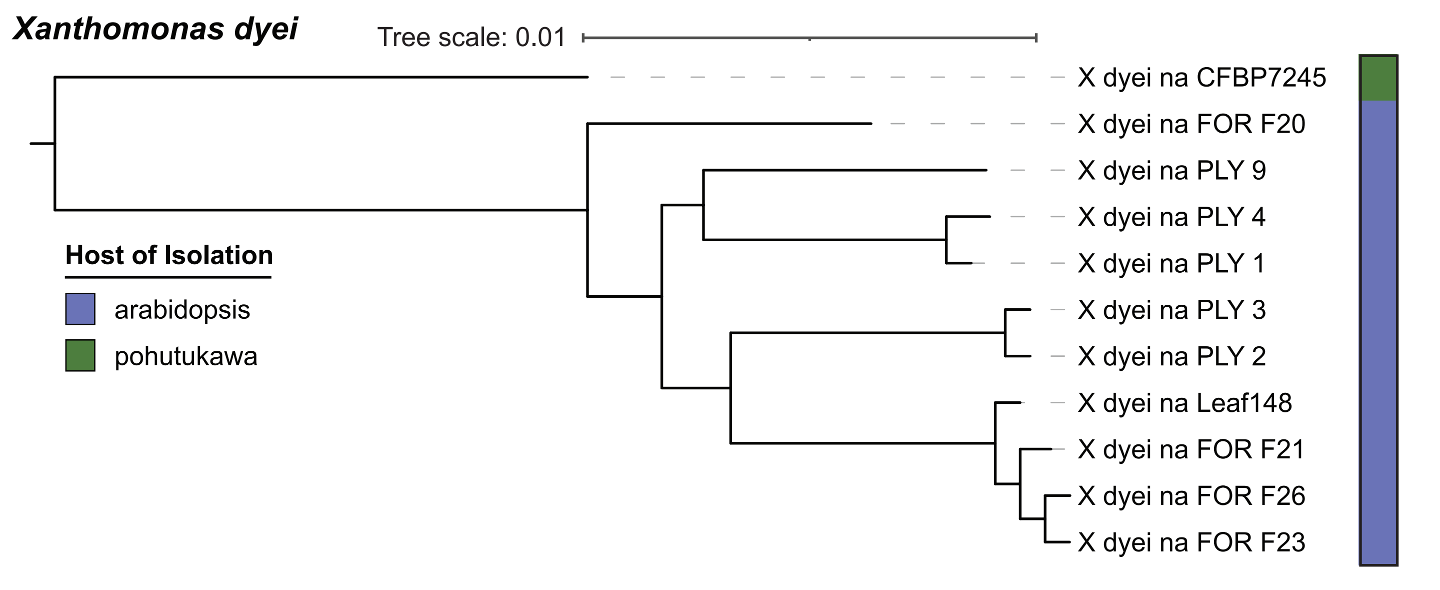

Xanthomonas dyei
Tree scale: 0.01
X dyei na CFBP7245
X dyei na FOR F20
X dyei na PLY 9
X dyei na PLY 4
X dyei na PLY 1
X dyei na PLY 3
X dyei na PLY 2
X dyei na Leaf148
X dyei na FOR F21
X dyei na FOR F26
X dyei na FOR F23
Host of Isolation
arabidopsis
pohutukawa

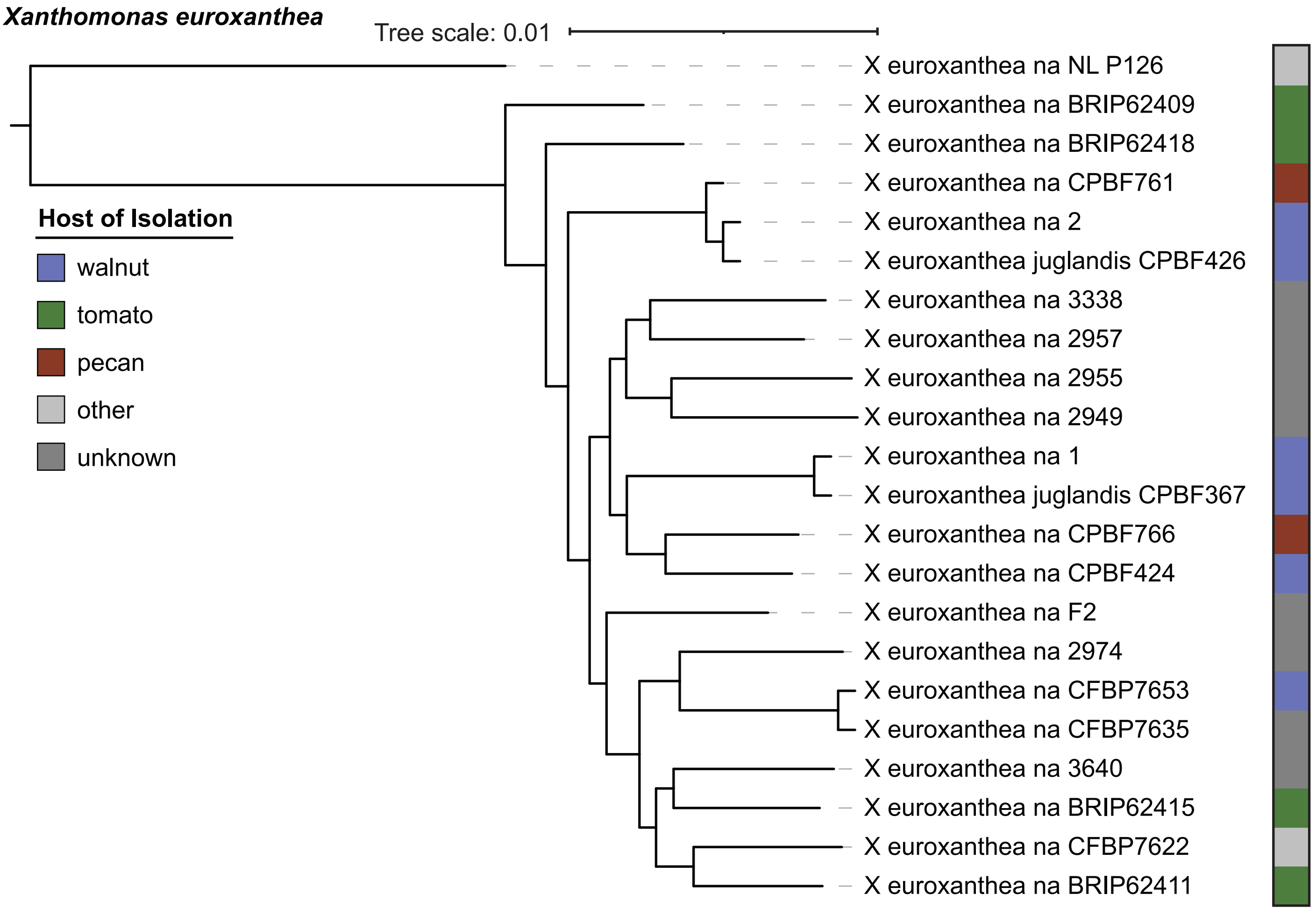

Xanthomonas euroxanthea
Tree scale: 0.01
Host of Isolation
walnut
tomato
pecan
other
unknown
X euroxanthea na NL P126
X euroxanthea na BRIP62409
X euroxanthea na BRIP62418
X euroxanthea na CPBF761
X euroxanthea na 2
X euroxanthea juglandis CPBF426
X euroxanthea na 3338
X euroxanthea na 2957
X euroxanthea na 2955
X euroxanthea na 2949
X euroxanthea na 1
X euroxanthea juglandis CPBF367
X euroxanthea na CPBF766
X euroxanthea na CPBF424
X euroxanthea na F2
X euroxanthea na 2974
X euroxanthea na CFBP7653
X euroxanthea na CFBP7635
X euroxanthea na 3640
X euroxanthea na BRIP62415
X euroxanthea na CFBP7622
X euroxanthea na BRIP62411

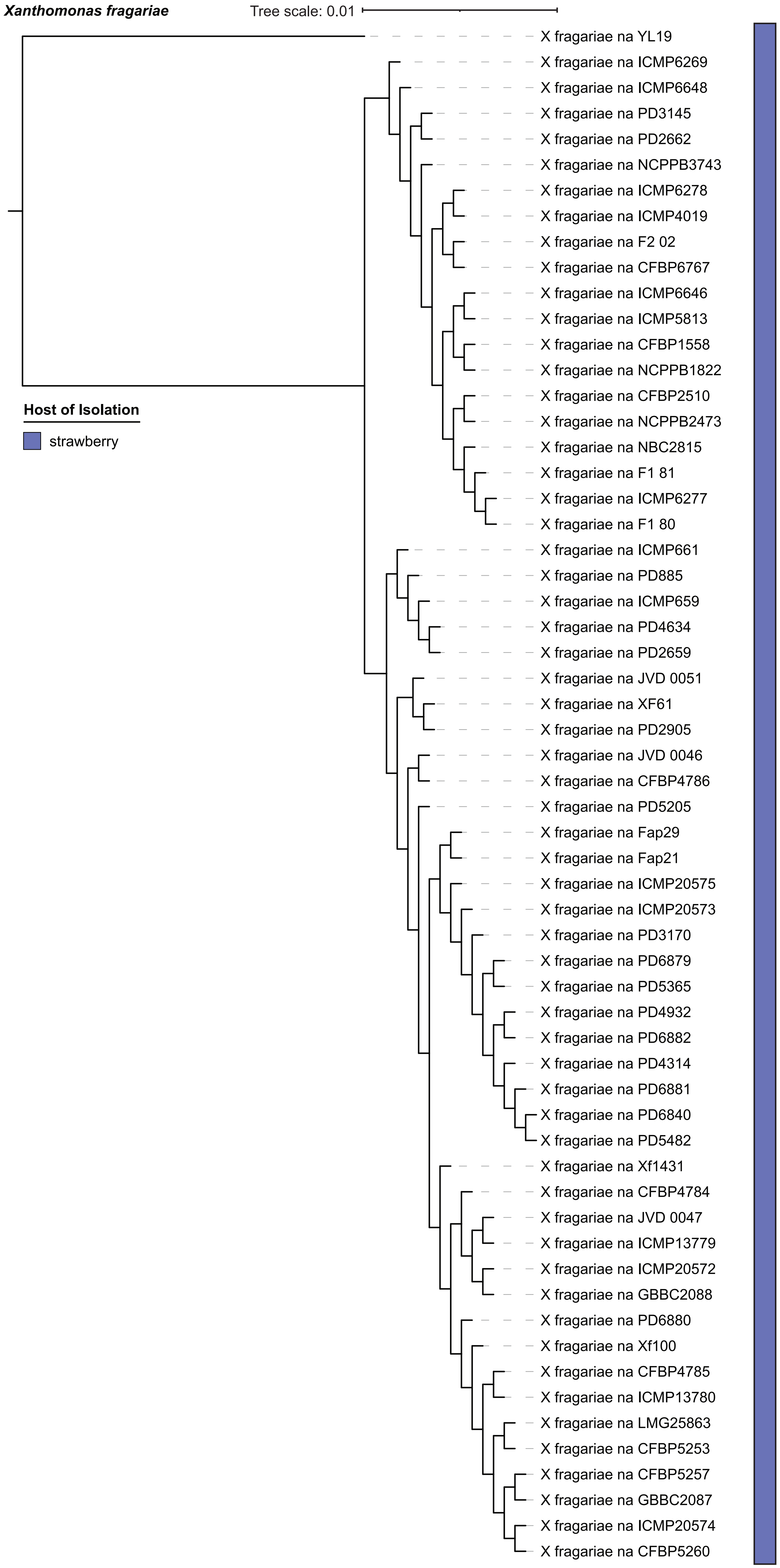
Xanthomonas fragariae
Tree scale: 0.01
X fragariae na YL19
X fragariae na ICMP6269
X fragariae na ICMP6648
X fragariae na PD3145
X fragariae na PD2662
X fragariae na NCPPB3743
X fragariae na ICMP6278
X fragariae na ICMP4019
X fragariae na F2 02
X fragariae na CFBP6767
X fragariae na ICMP6646
X fragariae na ICMP5813
X fragariae na CFBP1558
X fragariae na NCPPB1822
X fragariae na CFBP2510
X fragariae na NCPPB2473
X fragariae na NBC2815
X fragariae na F1 81
X fragariae na ICMP6277
X fragariae na F1 80
X fragariae na ICMP661
X fragariae na PD885
X fragariae na ICMP659
X fragariae na PD4634
X fragariae na PD2659
X fragariae na JVD 0051
X fragariae na XF61
X fragariae na PD2905
X fragariae na JVD 0046
X fragariae na CFBP4786
X fragariae na PD5205
X fragariae na Fap29
X fragariae na Fap21
X fragariae na ICMP20575
X fragariae na ICMP20573
X fragariae na PD3170
X fragariae na PD6879
X fragariae na PD5365
X fragariae na PD4932
X fragariae na PD6882
X fragariae na PD4314
X fragariae na PD6881
X fragariae na PD6840
X fragariae na PD5482
X fragariae na Xf1431
X fragariae na CFBP4784
X fragariae na JVD 0047
X fragariae na ICMP13779
X fragariae na ICMP20572
X fragariae na GBBC2088
X fragariae na PD6880
X fragariae na Xf100
X fragariae na CFBP4785
X fragariae na ICMP13780
X fragariae na LMG25863
X fragariae na CFBP5253
X fragariae na CFBP5257
X fragariae na GBBC2087
X fragariae na ICMP20574
X fragariae na CFBP5260
Host of Isolation
strawberry

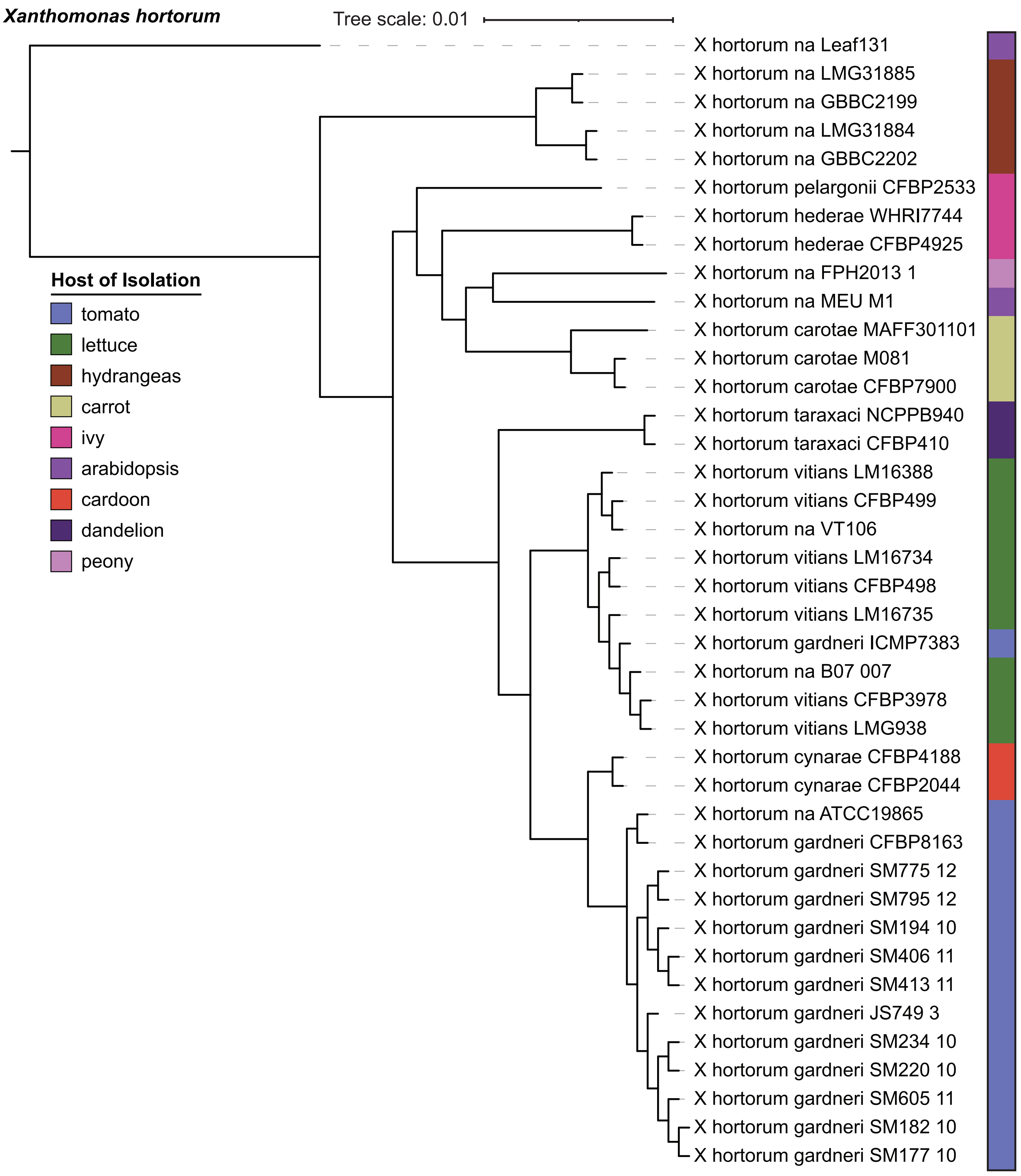

Xanthomonas hortorum
Tree scale: 0.01
X hortorum na Leaf131
X hortorum na LMG31885
X hortorum na GBBC2199
X hortorum na LMG31884
X hortorum na GBBC2202
X hortorum pelargonii CFBP2533
X hortorum hederae WHRI7744
X hortorum hederae CFBP4925
X hortorum na FPH2013 1
X hortorum na MEU M1
X hortorum carotae MAFF301101
X hortorum carotae M081
X hortorum carotae CFBP7900
X hortorum taraxaci NCPPB940
X hortorum taraxaci CFBP410
X hortorum vitians LM16388
X hortorum vitians CFBP499
X hortorum na VT106
X hortorum vitians LM16734
X hortorum vitians CFBP498
X hortorum vitians LM16735
X hortorum gardneri ICMP7383
X hortorum na B07 007
X hortorum vitians CFBP3978
X hortorum vitians LMG938
X hortorum cynarae CFBP4188
X hortorum cynarae CFBP2044
X hortorum na ATCC19865
X hortorum gardneri CFBP8163
X hortorum gardneri SM775 12
X hortorum gardneri SM795 12
X hortorum gardneri SM194 10
X hortorum gardneri SM406 11
X hortorum gardneri SM413 11
X hortorum gardneri JS749 3
X hortorum gardneri SM234 10
X hortorum gardneri SM220 10
X hortorum gardneri SM605 11
X hortorum gardneri SM182 10
X hortorum gardneri SM177 10
Host of Isolation
tomato
lettuce
hydrangeas
carrot
ivy
arabidopsis
cardoon
dandelion
peony

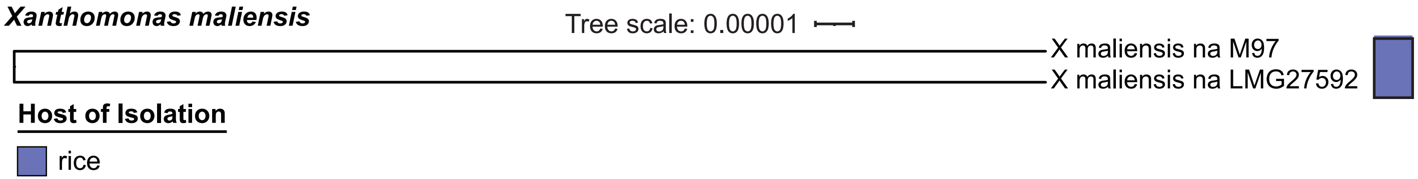
Xanthomonas maliensis
Tree scale: 0.00001
X maliensis na M97
X maliensis na LMG27592
Host of Isolation
rice

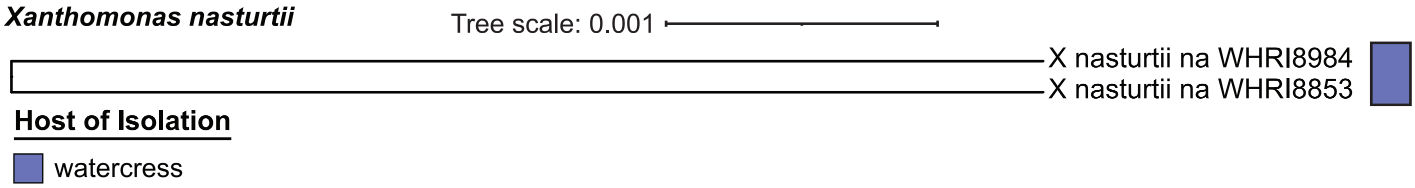

Xanthomonas nasturtii
Tree scale: 0.001
X nasturtii na WHRI8984
X nasturtii na WHRI8853
Host of Isolation
watercress

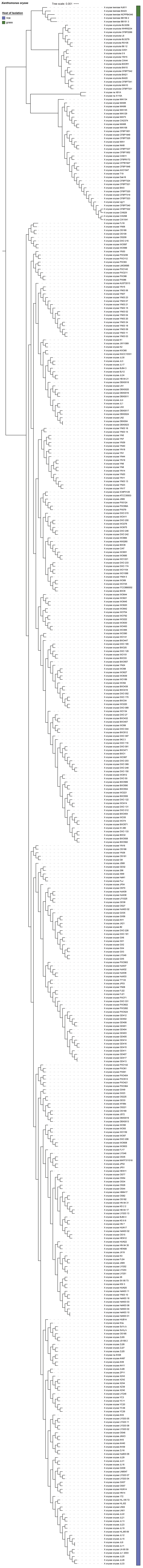
Xanthomonas oryzae
Tree scale: 0.001
Host of Isolation
rice
grass
X oryzae leersiae NJ611
X oryzae leersiae BAI23
X oryzae leersiae NCPPB4346
X oryzae leersiae BB156 2
X oryzae leersiae BB151 3
X oryzae oryzicola BLS256
X oryzae oryzicola WHRI5234
X oryzae oryzicola CFBP2286
X oryzae oryzicola L8
X oryzae oryzicola BLS279
X oryzae oryzicola RS105
X oryzae oryzicola B8 12
X oryzae oryzicola GX01
X oryzae oryzicola 0 9
X oryzae oryzicola YM15
X oryzae oryzicola CIX44
X oryzae oryzicola BXOR1
X oryzae oryzicola BAI15
X oryzae oryzicola CFBP7342
X oryzae oryzicola BAI21
X oryzae oryzicola BAI20
X oryzae oryzicola CFBP7341
X oryzae oryzicola MAI10
X oryzae oryzicola CFBP7331
X oryzae na X81A
X oryzae na X115A
X oryzae oryzae MAI134
X oryzae oryzae MAI95
X oryzae oryzae MAI99
X oryzae oryzae MAI145
X oryzae oryzae MAI129
X oryzae oryzae MAI73
X oryzae oryzae CIX2374
X oryzae oryzae MAI68

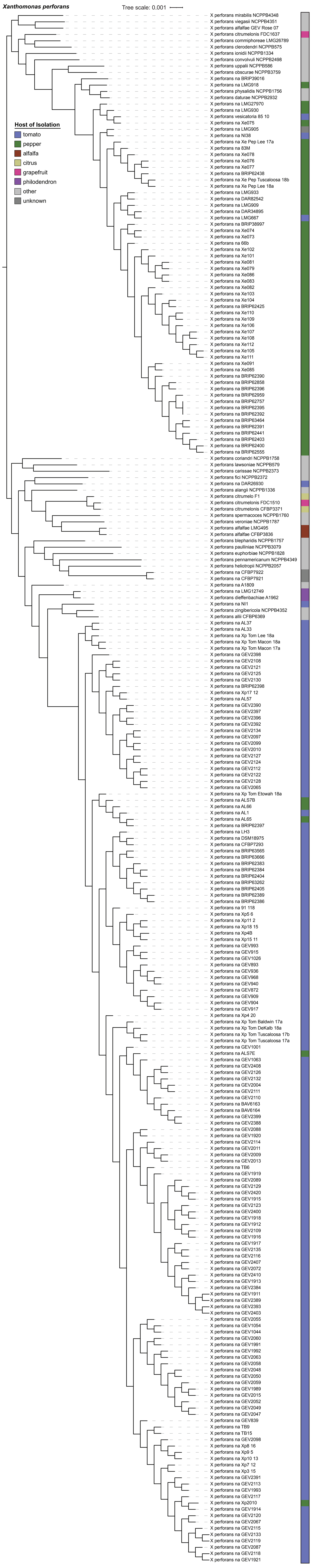

Xanthomonas perforans
Tree scale: 0.001
Host of Isolation
tomato
pepper
alfalfa
citrus
grapefruit
philodendron
other
unknown
X perforans mirabilis NCPPB4348
X perforans viegasii NCPPB4351
X perforans alfalfae GEV Rose 07
X perforans citrumelonis FDC1637
X perforans commiphoreae LMG26789
X perforans clerodendri NCPPB575
X perforans ionidii NCPPB1334
X perforans convolvuli NCPPB2498
X perforans uppalii NCPPB586
X perforans obscurae NCPPB3759
X perforans na BRIP39016
X perforans na LMG918
X perforans physalidis NCPPB1756
X perforans daturae NCPPB2932
X perforans na LMG27970
X perforans na LMG930
X perforans vesicatoria 85 10
X perforans na Xe075
X perforans na LMG905
X perforans na NI38
X perforans na Xe Pep Lee 17a
X perforans na 83M
X perforans na Xe078
X perforans na Xe076
X perforans na Xe077
X perforans na BRIP62438
X perforans na Xe Pep Tuscaloosa 18b
X perforans na Xe Pep Lee 18a
X perforans na LMG933
X perforans na DAR82542
X perforans na LMG909
X perforans na DAR34895
X perforans na LMG667
X perforans na BRIP38997
X perforans na Xe074
X perforans na Xe073
X perforans na 66b
X perforans na Xe102
X perforans na Xe101
X perforans na Xe081
X perforans na Xe079
X perforans na Xe086
X perforans na Xe083
X perforans na Xe082
X perforans na Xe103
X perforans na Xe104
X perforans na BRIP62425
X perforans na Xe110
X perforans na Xe109
X perforans na Xe106
X perforans na Xe107
X perforans na Xe108
X perforans na Xe112
X perforans na Xe105
X perforans na Xe111
X perforans na Xe091
X perforans na Xe085
X perforans na BRIP62390
X perforans na BRIP62858
X perforans na BRIP62396
X perforans na BRIP62959
X perforans na BRIP62757
X perforans na BRIP62395
X perforans na BRIP62392
X perforans na BRIP63464
X perforans na BRIP62391
X perforans na BRIP62441
X perforans na BRIP62403
X perforans na BRIP62400
X perforans na BRIP62555
X perforans coriandri NCPPB1758
X perforans lawsoniae NCPPB579
X perforans carissae NCPPB2373
X perforans fici NCPPB2372
X perforans na DAR26930
X perforans alangii NCPPB1336
X perforans citrumelo F1
X perforans citrumelonis FDC1510
X perforans citrumelonis CFBP3371
X perforans spermacoces NCPPB1760
X perforans veroniae NCPPB1787
X perforans alfalfae LMG495
X perforans alfalfae CFBP3836
X perforans blepharidis NCPPB1757
X perforans paulliniae NCPPB3079
X perforans euphorbiae NCPPB1828
X perforans pennamericanum NCPPB4349
X perforans heliotropii NCPPB2057
X perforans na CFBP7922
X perforans na CFBP7921
X perforans na A1809
X perforans na LMG12749
X perforans dieffenbachiae A1962
X perforans na NI1
X perforans zingibericola NCPPB4352
X perforans allii CFBP6369
X perforans na AL37
X perforans na AL33
X perforans na Xp Tom Lee 18a
X perforans na Xp Tom Macon 18a
X perforans na Xp Tom Macon 17a
X perforans na GEV2398
X perforans na GEV2108
X perforans na GEV2121
X perforans na GEV2125
X perforans na GEV2130
X perforans na BRIP62398
X perforans na Xp17 12
X perforans na AL57
X perforans na GEV2390
X perforans na GEV2397
X perforans na GEV2396
X perforans na GEV2392
X perforans na GEV2134
X perforans na GEV2097
X perforans na GEV2099
X perforans na GEV2010
X perforans na GEV2127
X perforans na GEV2124
X perforans na GEV2112
X perforans na GEV2122
X perforans na GEV2128
X perforans na GEV2065
X perforans na Xp Tom Etowah 18a
X perforans na ALS7B
X perforans na AL66
X perforans na AL1
X perforans na AL65
X perforans na BRIP62397
X perforans na LH3
X perforans na DSM18975
X perforans na CFBP7293
X perforans na BRIP63565
X perforans na BRIP63666
X perforans na BRIP62383
X perforans na BRIP62384
X perforans na BRIP62404
X perforans na BRIP63262
X perforans na BRIP62405
X perforans na BRIP62389
X perforans na BRIP62386
X perforans na 91 118
X perforans na Xp5 6
X perforans na Xp11 2
X perforans na Xp18 15
X perforans na Xp4B
X perforans na Xp15 11
X perforans na GEV993
X perforans na GEV915
X perforans na GEV1026
X perforans na GEV893
X perforans na GEV936
X perforans na GEV968
X perforans na GEV940
X perforans na GEV872
X perforans na GEV909
X perforans na GEV904
X perforans na GEV917
X perforans na Xp4 20
X perforans na Xp Tom Baldwin 17a
X perforans na Xp Tom DeKalb 18a
X perforans na Xp Tom Tuscaloosa 17b
X perforans na Xp Tom Tuscaloosa 17a
X perforans na GEV1001
X perforans na ALS7E
X perforans na GEV1063
X perforans na GEV2408
X perforans na GEV2126
X perforans na GEV2132
X perforans na GEV2004
X perforans na GEV2111
X perforans na GEV2110
X perforans na BAV6163
X perforans na BAV6164
X perforans na GEV2399
X perforans na GEV2388
X perforans na GEV2088
X perforans na GEV1920
X perforans na GEV2114
X perforans na GEV2011
X perforans na GEV2009
X perforans na GEV2013
X perforans na TB6
X perforans na GEV1919
X perforans na GEV2089
X perforans na GEV2129
X perforans na GEV2420
X perforans na GEV1915
X perforans na GEV2123
X perforans na GEV2400
X perforans na GEV1918
X perforans na GEV1912
X perforans na GEV2109
X perforans na GEV1916
X perforans na GEV1917
X perforans na GEV2135
X perforans na GEV2116
X perforans na GEV2407
X perforans na GEV2072
X perforans na GEV2410
X perforans na GEV1913
X perforans na GEV2384
X perforans na GEV1911
X perforans na GEV2389
X perforans na GEV2393
X perforans na GEV2403
X perforans na GEV2055
X perforans na GEV1054
X perforans na GEV1044
X perforans na GEV2060
X perforans na GEV1991
X perforans na GEV1992
X perforans na GEV2063
X perforans na GEV2058
X perforans na GEV2048
X perforans na GEV2050
X perforans na GEV2059
X perforans na GEV1989
X perforans na GEV2015
X perforans na GEV2052
X perforans na GEV2049
X perforans na GEV2047
X perforans na GEV839
X perforans na TB9
X perforans na TB15
X perforans na GEV2098
X perforans na Xp8 16
X perforans na Xp9 5
X perforans na Xp10 13
X perforans na Xp7 12
X perforans na Xp3 15
X perforans na GEV2391
X perforans na GEV2113
X perforans na GEV1993
X perforans na GEV2117
X perforans na Xp2010
X perforans na GEV1914
X perforans na GEV2120
X perforans na GEV2067
X perforans na GEV2115
X perforans na GEV2133
X perforans na GEV2119
X perforans na GEV2087
X perforans na GEV2118
X perforans na GEV1921

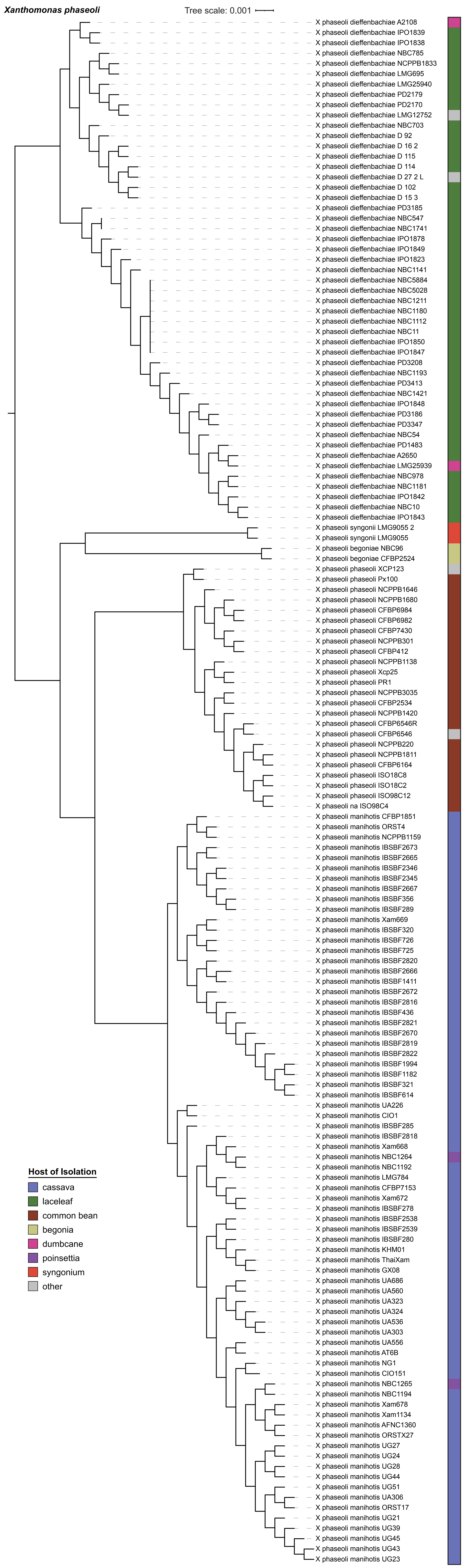
Xanthomonas phaseoli
Tree scale: 0.001
X phaseoli dieffenbachiae A2108
X phaseoli dieffenbachiae IPO1839
X phaseoli dieffenbachiae IPO1838
X phaseoli dieffenbachiae NBC785
X phaseoli dieffenbachiae NCPPB1833
X phaseoli dieffenbachiae LMG695
X phaseoli dieffenbachiae LMG25940
X phaseoli dieffenbachiae PD2179
X phaseoli dieffenbachiae PD2170
X phaseoli dieffenbachiae LMG12752
X phaseoli dieffenbachiae NBC703
X phaseoli dieffenbachiae D 92
X phaseoli dieffenbachiae D 16 2
X phaseoli dieffenbachiae D 115
X phaseoli dieffenbachiae D 114
X phaseoli dieffenbachiae D 27 2 L
X phaseoli dieffenbachiae D 102
X phaseoli dieffenbachiae D 15 3
X phaseoli dieffenbachiae PD3185
X phaseoli dieffenbachiae NBC547
X phaseoli dieffenbachiae NBC1741
X phaseoli dieffenbachiae IPO1878
X phaseoli dieffenbachiae IPO1849
X phaseoli dieffenbachiae IPO1823
X phaseoli dieffenbachiae NBC1141
X phaseoli dieffenbachiae NBC5884
X phaseoli dieffenbachiae NBC5028
X phaseoli dieffenbachiae NBC1211
X phaseoli dieffenbachiae NBC1180
X phaseoli dieffenbachiae NBC1112
X phaseoli dieffenbachiae NBC11
X phaseoli dieffenbachiae IPO1850
X phaseoli dieffenbachiae IPO1847
X phaseoli dieffenbachiae PD3208
X phaseoli dieffenbachiae NBC1193
X phaseoli dieffenbachiae PD3413
X phaseoli dieffenbachiae NBC1421
X phaseoli dieffenbachiae IPO1848
X phaseoli dieffenbachiae PD3186
X phaseoli dieffenbachiae PD3347
X phaseoli dieffenbachiae NBC54
X phaseoli dieffenbachiae PD1483
X phaseoli dieffenbachiae A2650
X phaseoli dieffenbachiae LMG25939
X phaseoli dieffenbachiae NBC978
X phaseoli dieffenbachiae NBC1181
X phaseoli dieffenbachiae IPO1842
X phaseoli dieffenbachiae NBC10
X phaseoli dieffenbachiae IPO1843
X phaseoli syngonii LMG9055 2
X phaseoli syngonii LMG9055
X phaseoli begoniae NBC96
X phaseoli begoniae CFBP2524
X phaseoli phaseoli XCP123
X phaseoli phaseoli Px100
X phaseoli phaseoli NCPPB1646
X phaseoli phaseoli NCPPB1680
X phaseoli phaseoli CFBP6984
X phaseoli phaseoli CFBP6982
X phaseoli phaseoli CFBP7430
X phaseoli phaseoli NCPPB301
X phaseoli phaseoli CFBP412
X phaseoli phaseoli NCPPB1138
X phaseoli phaseoli Xcp25
X phaseoli phaseoli PR1
X phaseoli phaseoli NCPPB3035
X phaseoli phaseoli CFBP2534
X phaseoli phaseoli NCPPB1420
X phaseoli phaseoli CFBP6546R
X phaseoli phaseoli CFBP6546
X phaseoli phaseoli NCPPB220
X phaseoli phaseoli NCPPB1811
X phaseoli phaseoli CFBP6164
X phaseoli phaseoli ISO18C8
X phaseoli phaseoli ISO18C2
X phaseoli phaseoli ISO98C12
X phaseoli na ISO98C4
X phaseoli manihotis CFBP1851
X phaseoli manihotis ORST4
X phaseoli manihotis NCPPB1159
X phaseoli manihotis IBSBF2673
X phaseoli manihotis IBSBF2665
X phaseoli manihotis IBSBF2346
X phaseoli manihotis IBSBF2345
X phaseoli manihotis IBSBF2667
X phaseoli manihotis IBSBF356
X phaseoli manihotis IBSBF289
X phaseoli manihotis Xam669
X phaseoli manihotis IBSBF320
X phaseoli manihotis IBSBF726
X phaseoli manihotis IBSBF725
X phaseoli manihotis IBSBF2820
X phaseoli manihotis IBSBF2666
X phaseoli manihotis IBSBF1411
X phaseoli manihotis IBSBF2672
X phaseoli manihotis IBSBF2816
X phaseoli manihotis IBSBF436
X phaseoli manihotis IBSBF2821
X phaseoli manihotis IBSBF2670
X phaseoli manihotis IBSBF2819
X phaseoli manihotis IBSBF2822
X phaseoli manihotis IBSBF1994
X phaseoli manihotis IBSBF1182
X phaseoli manihotis IBSBF321
X phaseoli manihotis IBSBF614
X phaseoli manihotis UA226
X phaseoli manihotis CIO1
X phaseoli manihotis IBSBF285
X phaseoli manihotis IBSBF2818
X phaseoli manihotis Xam668
X phaseoli manihotis NBC1264
X phaseoli manihotis NBC1192
X phaseoli manihotis LMG784
X phaseoli manihotis CFBP7153
X phaseoli manihotis Xam672
X phaseoli manihotis IBSBF278
X phaseoli manihotis IBSBF2538
X phaseoli manihotis IBSBF2539
X phaseoli manihotis IBSBF280
X phaseoli manihotis KHM01
X phaseoli manihotis ThaiXam
X phaseoli manihotis GX08
X phaseoli manihotis UA686
X phaseoli manihotis UA560
X phaseoli manihotis UA323
X phaseoli manihotis UA324
X phaseoli manihotis UA536
X phaseoli manihotis UA303
X phaseoli manihotis UA556
X phaseoli manihotis AT6B
X phaseoli manihotis NG1
X phaseoli manihotis CIO151
X phaseoli manihotis NBC1265
X phaseoli manihotis NBC1194
X phaseoli manihotis Xam678
X phaseoli manihotis Xam1134
X phaseoli manihotis AFNC1360
X phaseoli manihotis ORSTX27
X phaseoli manihotis UG27
X phaseoli manihotis UG24
X phaseoli manihotis UG28
X phaseoli manihotis UG44
X phaseoli manihotis UG51
X phaseoli manihotis UA306
X phaseoli manihotis ORST17
X phaseoli manihotis UG21
X phaseoli manihotis UG39
X phaseoli manihotis UG45
X phaseoli manihotis UG43
X phaseoli manihotis UG23
Host of Isolation
cassava
laceleaf
common bean
begonia
dumbcane
poinsettia
syngonium
other

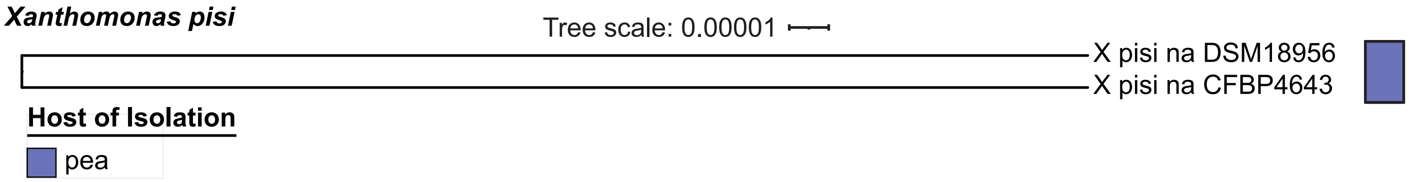
Xanthomonas pisi
Tree scale: 0.00001
X pisi na DSM18956
X pisi na CFBP4643
Host of Isolation
pea

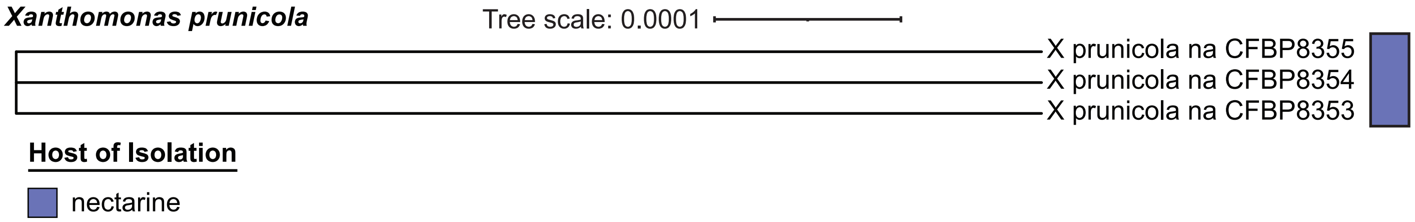
Xanthomonas prunicola
Tree scale: 0.0001
X prunicola na CFBP8355
X prunicola na CFBP8354
X prunicola na CFBP8353
Host of Isolation
nectarine

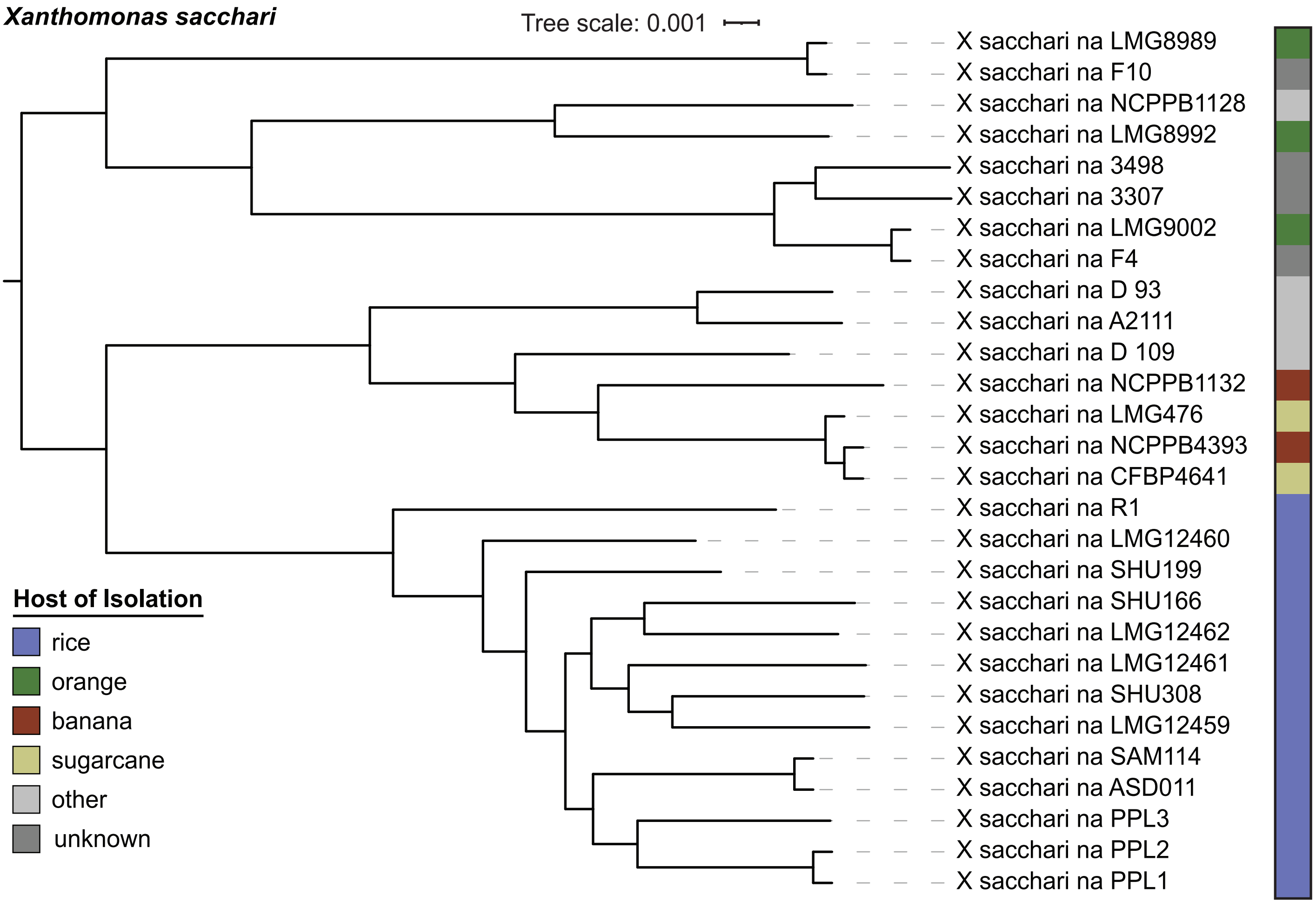
Xanthomonas sacchari
Tree scale: 0.001
X sacchari na LMG8989
X sacchari na F10
X sacchari na NCPPB1128
X sacchari na LMG8992
X sacchari na 3498
X sacchari na 3307
X sacchari na LMG9002
X sacchari na F4
X sacchari na D 93
X sacchari na A2111
X sacchari na D 109
X sacchari na NCPPB1132
X sacchari na LMG476
X sacchari na NCPPB4393
X sacchari na CFBP4641
X sacchari na R1
X sacchari na LMG12460
X sacchari na SHU199
X sacchari na SHU166
X sacchari na LMG12462
X sacchari na LMG12461
X sacchari na SHU308
X sacchari na LMG12459
X sacchari na SAM114
X sacchari na ASD011
X sacchari na PPL3
X sacchari na PPL2
X sacchari na PPL1
Host of Isolation
rice
orange
banana
sugarcane
other
unknown

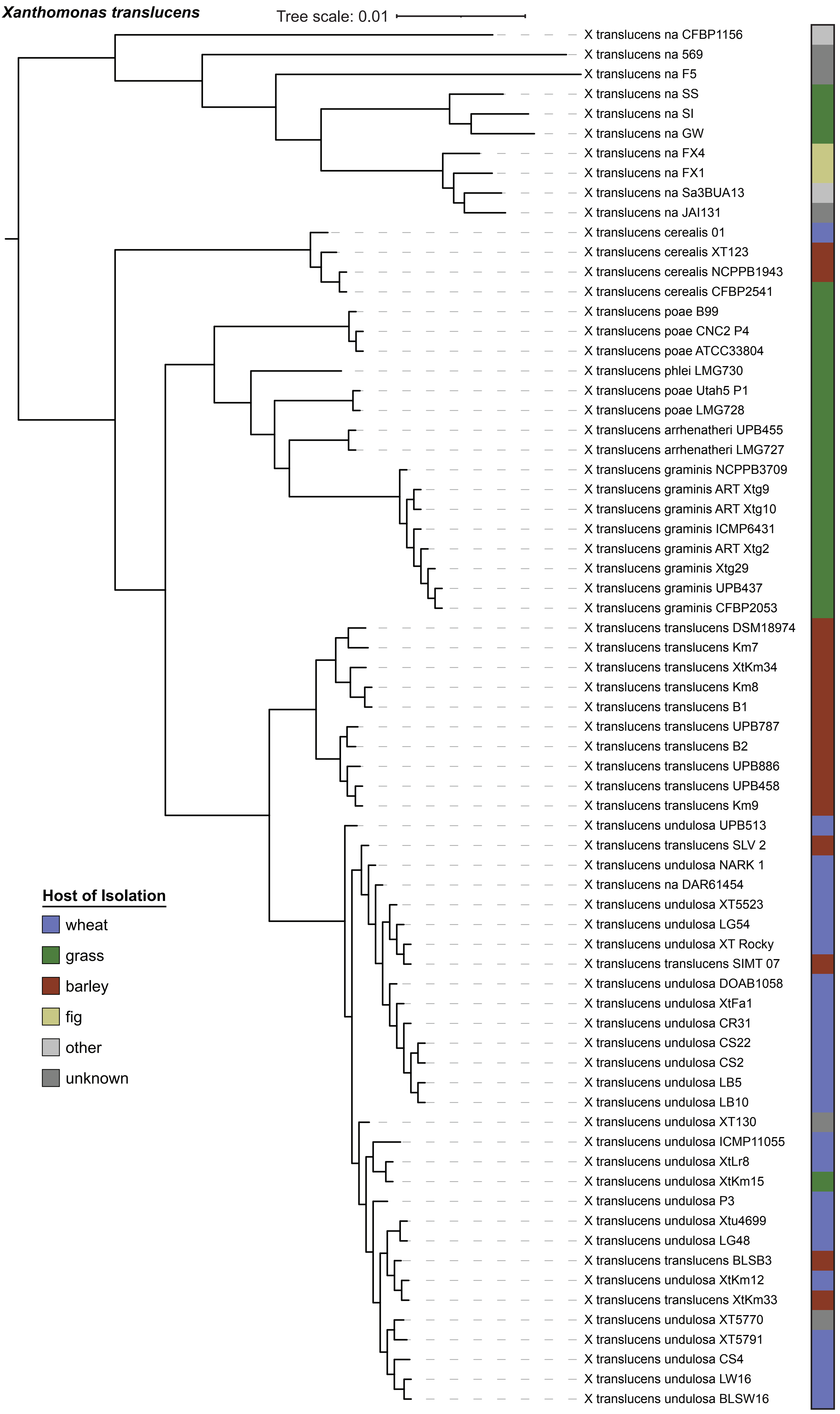

Xanthomonas translucens
Tree scale: 0.01
X translucens na CFBP1156
X translucens na 569
X translucens na F5
X translucens na SS
X translucens na SI
X translucens na GW
X translucens na FX4
X translucens na FX1
X translucens na Sa3BUA13
X translucens na JAI131
X translucens cerealis 01
X translucens cerealis XT123
X translucens cerealis NCPPB1943
X translucens cerealis CFBP2541
X translucens poae B99
X translucens poae CNC2 P4
X translucens poae ATCC33804
X translucens phlei LMG730
X translucens poae Utah5 P1
X translucens poae LMG728
X translucens arrhenatheri UPB455
X translucens arrhenatheri LMG727
X translucens graminis NCPPB3709
X translucens graminis ART Xtg9
X translucens graminis ART Xtg10
X translucens graminis ICMP6431
X translucens graminis ART Xtg2
X translucens graminis Xtg29
X translucens graminis UPB437
X translucens graminis CFBP2053
X translucens translucens DSM18974
X translucens translucens Km7
X translucens translucens XtKm34
X translucens translucens Km8
X translucens translucens B1
X translucens translucens UPB787
X translucens translucens B2
X translucens translucens UPB886
X translucens translucens UPB458
X translucens translucens Km9
X translucens undulosa UPB513
X translucens translucens SLV 2
X translucens undulosa NARK 1
X translucens na DAR61454
X translucens undulosa XT5523
X translucens undulosa LG54
X translucens undulosa XT Rocky
X translucens translucens SIMT 07
X translucens undulosa DOAB1058
X translucens undulosa XtFa1
X translucens undulosa CR31
X translucens undulosa CS22
X translucens undulosa CS2
X translucens undulosa LB5
X translucens undulosa LB10
X translucens undulosa XT130
X translucens undulosa ICMP11055
X translucens undulosa XtLr8
X translucens undulosa XtKm15
X translucens undulosa P3
X translucens undulosa Xtu4699
X translucens undulosa LG48
X translucens translucens BLSB3
X translucens undulosa XtKm12
X translucens translucens XtKm33
X translucens undulosa XT5770
X translucens undulosa XT5791
X translucens undulosa CS4
X translucens undulosa LW16
X translucens undulosa BLSW16
Host of Isolation
wheat
grass
barley
fig
other
unknown

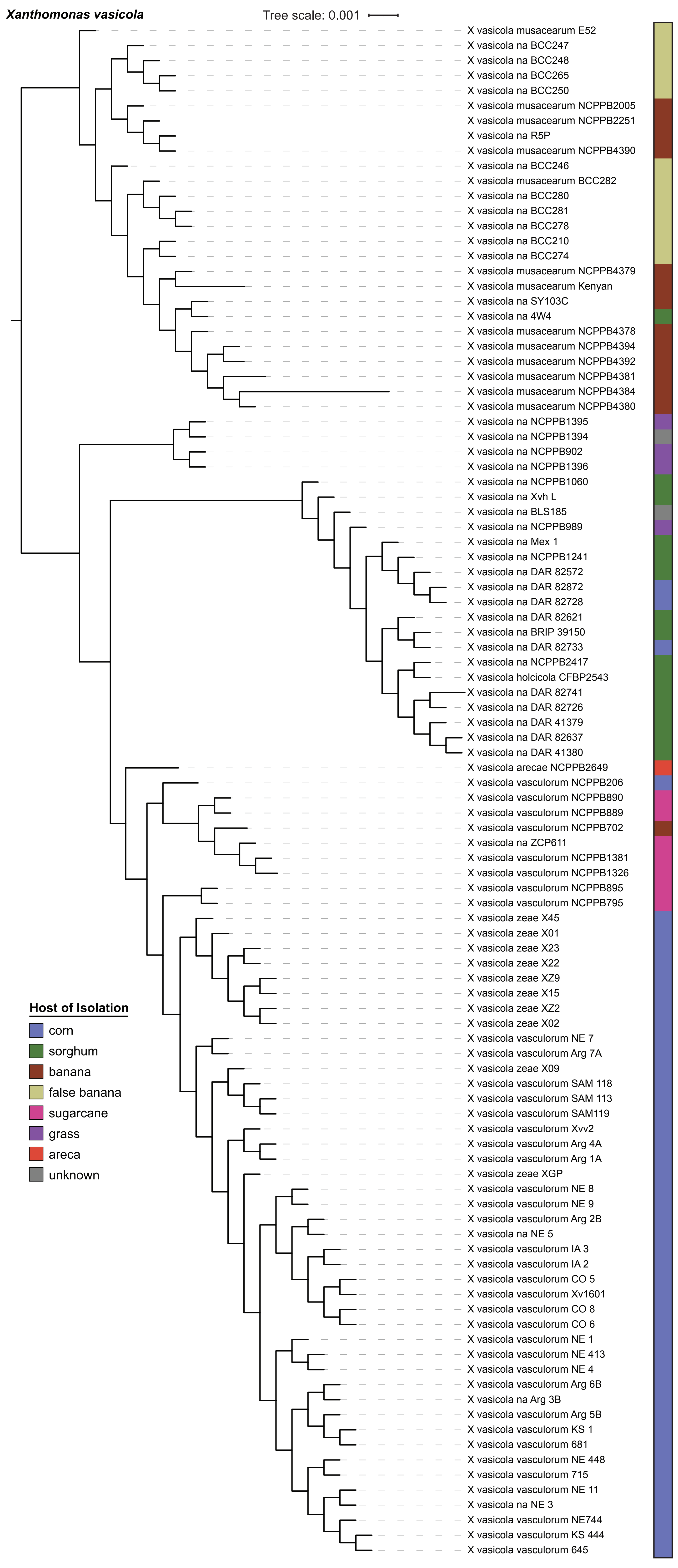

Xanthomonas vasicola
Tree scale: 0.001
X vasicola musacearum E52
X vasicola na BCC247
X vasicola na BCC248
X vasicola na BCC265
X vasicola na BCC250
X vasicola musacearum NCPPB2005
X vasicola musacearum NCPPB2251
X vasicola na R5P
X vasicola musacearum NCPPB4390
X vasicola na BCC246
X vasicola musacearum BCC282
X vasicola na BCC280
X vasicola na BCC281
X vasicola na BCC278
X vasicola na BCC210
X vasicola na BCC274
X vasicola musacearum NCPPB4379
X vasicola musacearum Kenyan
X vasicola na SY103C
X vasicola na 4W4
X vasicola musacearum NCPPB4378
X vasicola musacearum NCPPB4394
X vasicola musacearum NCPPB4392
X vasicola musacearum NCPPB4381
X vasicola musacearum NCPPB4384
X vasicola musacearum NCPPB4380
X vasicola na NCPPB1395
X vasicola na NCPPB1394
X vasicola na NCPPB902
X vasicola na NCPPB1396
X vasicola na NCPPB1060
X vasicola na Xvh L
X vasicola na BLS185
X vasicola na NCPPB989
X vasicola na Mex 1
X vasicola na NCPPB1241
X vasicola na DAR 82572
X vasicola na DAR 82872
X vasicola na DAR 82728
X vasicola na DAR 82621
X vasicola na BRIP 39150
X vasicola na DAR 82733
X vasicola na NCPPB2417
X vasicola holcicola CFBP2543
X vasicola na DAR 82741
X vasicola na DAR 82726
X vasicola na DAR 41379
X vasicola na DAR 82637
X vasicola na DAR 41380
X vasicola arecae NCPPB2649
X vasicola vasculorum NCPPB206
X vasicola vasculorum NCPPB890
X vasicola vasculorum NCPPB889
X vasicola vasculorum NCPPB702
X vasicola na ZCP611
X vasicola vasculorum NCPPB1381
X vasicola vasculorum NCPPB1326
X vasicola vasculorum NCPPB895
X vasicola vasculorum NCPPB795
X vasicola zeae X45
X vasicola zeae X01
X vasicola zeae X23
X vasicola zeae X22
X vasicola zeae XZ9
X vasicola zeae X15
X vasicola zeae XZ2
X vasicola zeae X02
X vasicola vasculorum NE 7
X vasicola vasculorum Arg 7A
X vasicola zeae X09
X vasicola vasculorum SAM 118
X vasicola vasculorum SAM 113
X vasicola vasculorum SAM119
X vasicola vasculorum Xvv2
X vasicola vasculorum Arg 4A
X vasicola vasculorum Arg 1A
X vasicola zeae XGP
X vasicola vasculorum NE 8
X vasicola vasculorum NE 9
X vasicola vasculorum Arg 2B
X vasicola na NE 5
X vasicola vasculorum IA 3
X vasicola vasculorum IA 2
X vasicola vasculorum CO 5
X vasicola vasculorum Xv1601
X vasicola vasculorum CO 8
X vasicola vasculorum CO 6
X vasicola vasculorum NE 1
X vasicola vasculorum NE 413
X vasicola vasculorum NE 4
X vasicola vasculorum Arg 6B
X vasicola na Arg 3B
X vasicola vasculorum Arg 5B
X vasicola vasculorum KS 1
X vasicola vasculorum 681
X vasicola vasculorum NE 448
X vasicola vasculorum 715
X vasicola vasculorum NE 11
X vasicola na NE 3
X vasicola vasculorum NE744
X vasicola vasculorum KS 444
X vasicola vasculorum 645
Host of Isolation
corn
sorghum
banana
false banana
sugarcane
grass
areca
unknown

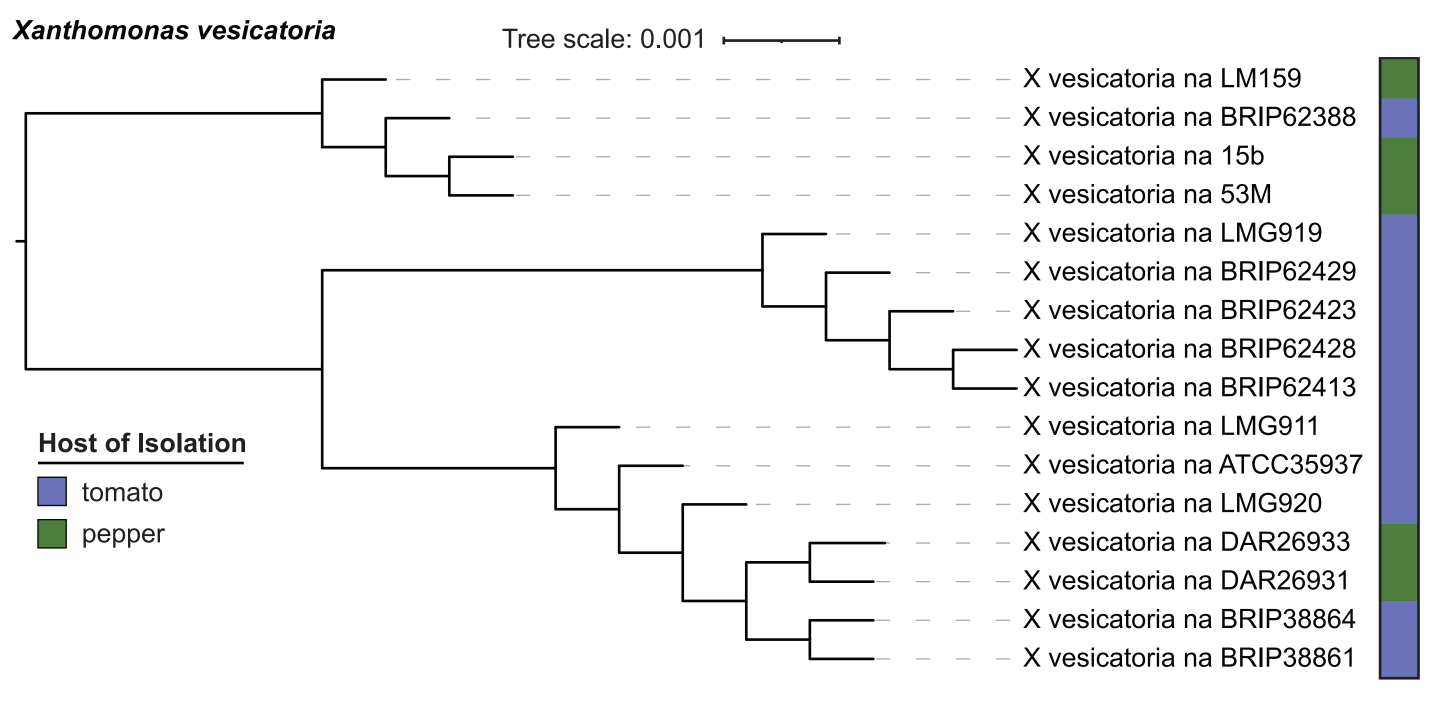
Xanthomonas vesicatoria
Tree scale: 0.001
X vesicatoria na LM159
X vesicatoria na BRIP62388
X vesicatoria na 15b
X vesicatoria na 53M
X vesicatoria na LMG919
X vesicatoria na BRIP62429
X vesicatoria na BRIP62423
X vesicatoria na BRIP62428
X vesicatoria na BRIP62413
X vesicatoria na LMG911
X vesicatoria na ATCC35937
X vesicatoria na LMG920
X vesicatoria na DAR26933
X vesicatoria na DAR26931
X vesicatoria na BRIP38864
X vesicatoria na BRIP38861
Host of Isolation
tomato
pepper
