# Supplementary figures and images for: Pangenome insights into the diversification and disease specificity of worldwide Xanthomonas outbreaks
Source: Front Microbiol. 2023 Jul 5;14:1213261. doi: 10.3389/fmicb.2023.1213261 (PMC10356107; doi:10.3389/fmicb.2023.1213261)

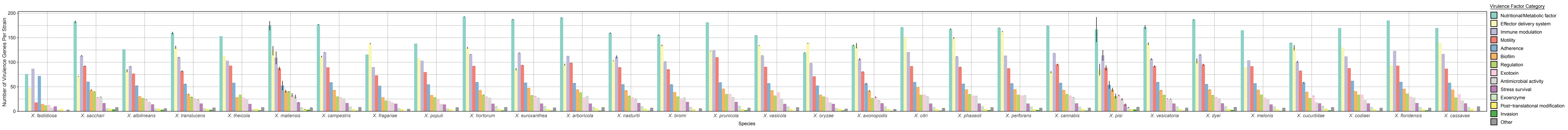

Supplement: Supplementary file 7 [file Image_5.PDF]

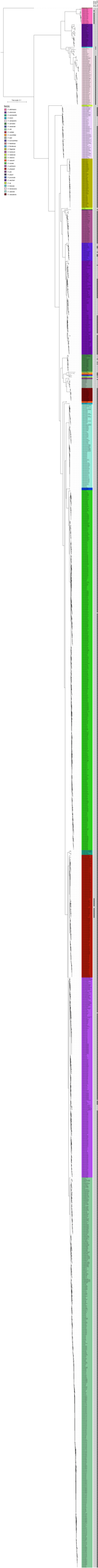

Supplement: Supplementary file 9 [file Image_7.PDF]

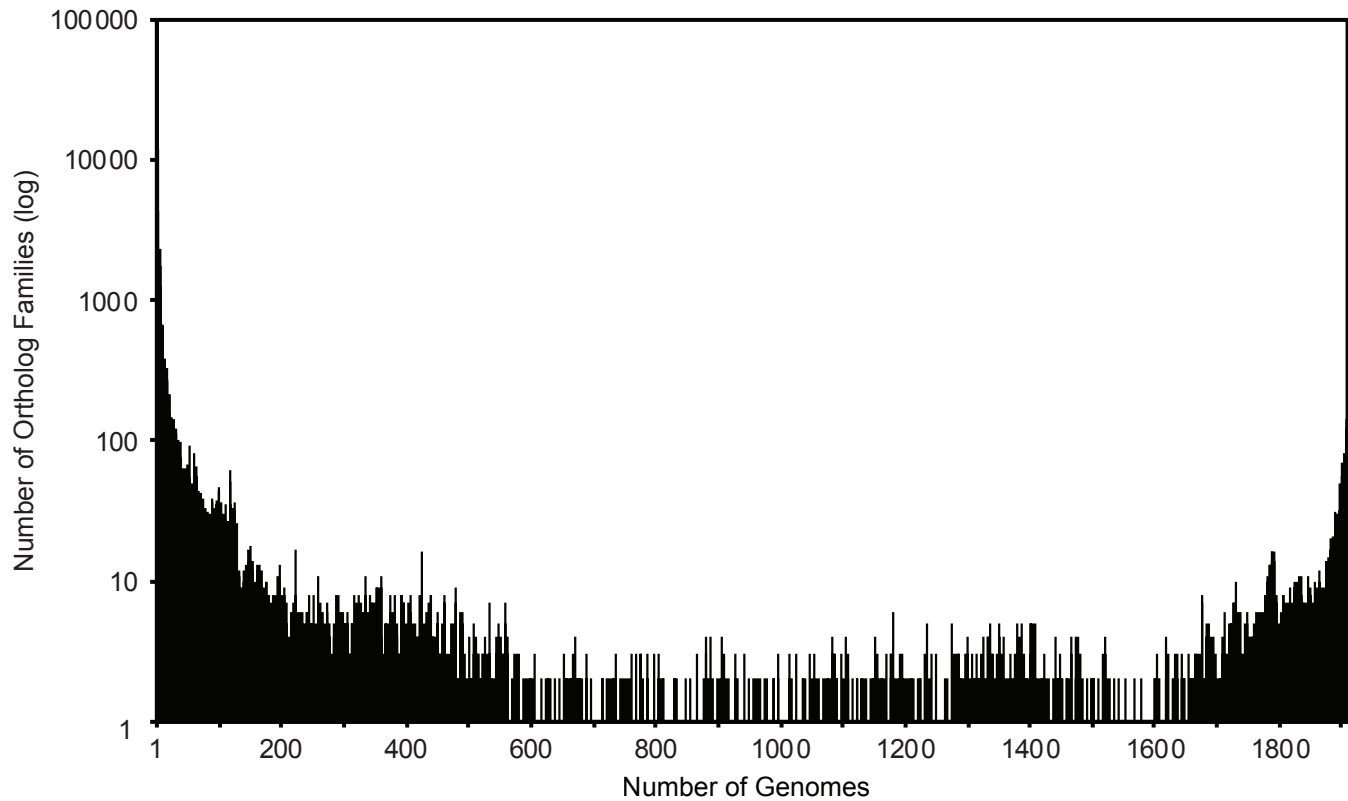

Supplement: Supplementary file 10 [file Image_8.PDF]
