# Supplementary material for: Pangenome insights into the diversification and disease specificity of worldwide Xanthomonas outbreaks
Source: Front Microbiol. 2023 Jul 5;14:1213261. doi: 10.3389/fmicb.2023.1213261 (PMC10356107; doi:10.3389/fmicb.2023.1213261)
Supplement: Supplementary file 8 [file Image_6.PDF]

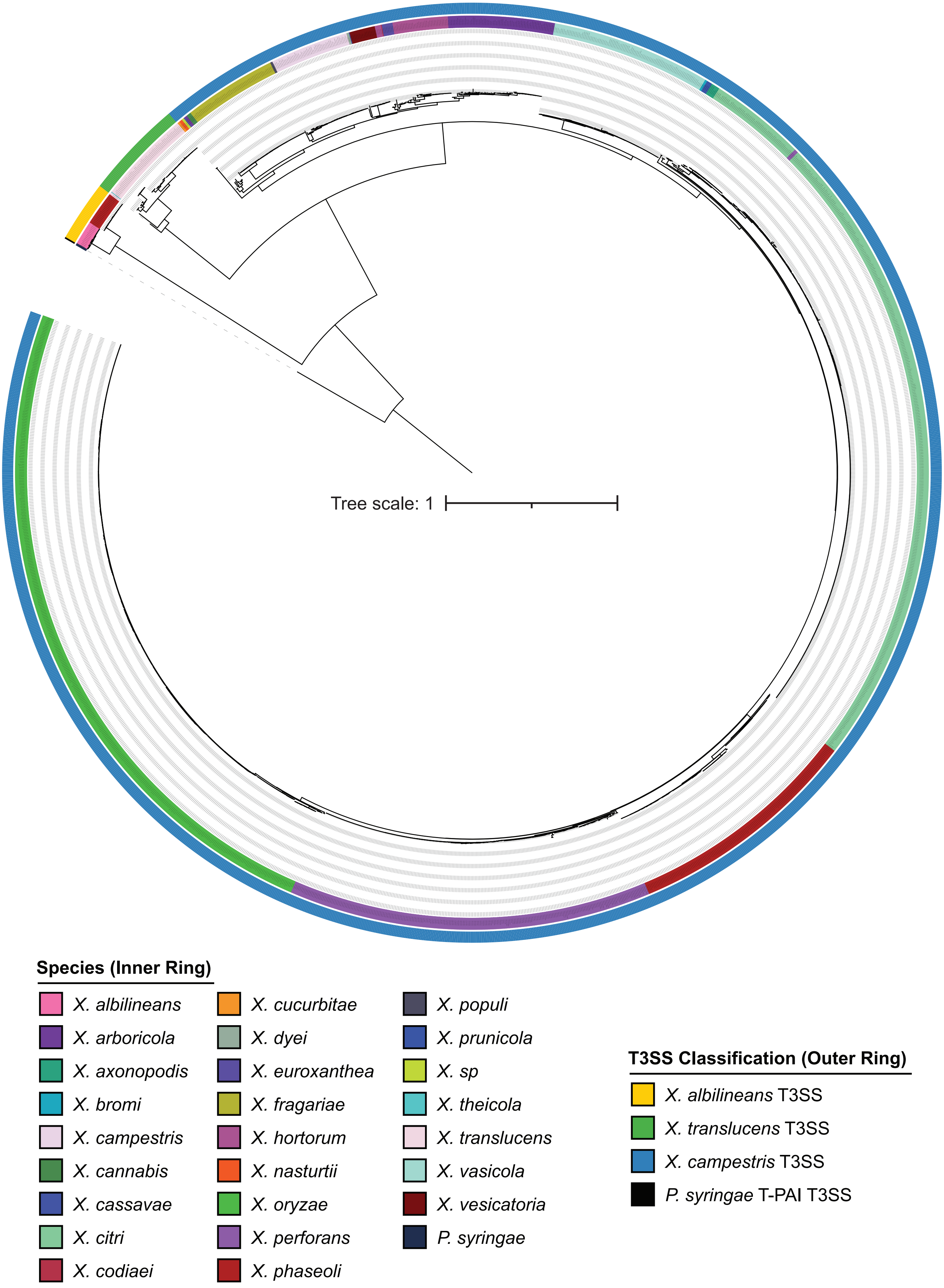
Tree scale: 1
Species (Inner Ring)
X. albilineans
X. arboricola
X. axonopodis
X. bromi
X. campestris
X. cannabis
X. cassavae
X. citri
X. codiaei
X. cucurbitae
X. dyei
X. euroxanthea
X. fragariae
X. hortorum
X. nasturtii
X. oryzae
X. perforans
X. phaseoli
X. populi
X. prunicola
X. sp
X. theicola
X. translucens
X. vasicola
X. vesicatoria
P. syringae
T3SS Classification (Outer Ring)
X. albilineans T3SS
X. translucens T3SS
X. campestris T3SS
P. syringae T-PAI T3SS
