# Supplementary material for: Pangenome insights into the diversification and disease specificity of worldwide Xanthomonas outbreaks
Source: Front Microbiol. 2023 Jul 5;14:1213261. doi: 10.3389/fmicb.2023.1213261 (PMC10356107; doi:10.3389/fmicb.2023.1213261)
Supplement: Supplementary file 11 [file Table_1.DOCX]

**Supplemental Table S1:** Representative *hrp* and *hrc* genes used for the identification of type III secretion system (T3SS) pathogenicity islands in this study.

| Primary Name | Alternate Name(s) | Annotation | Protein Sequence |
| --- | --- | --- | --- |
| HrcC | YscC | Outer membrane ring protein | MAYACPPVHRHRRAPLAAALLLGLLPLLPPHANAASVPWHSRSFKYVADRKDLKEVLRDLSASQSITTWISPEVTGTLSGKFEATPQKFLDDLSGTFGFVWYYDGSVLRIWGANETKNATLSLGAASTSALRDALARMRLDDPRFPVRYDETAHLAVVSGPPGYVDTVAAIAKQVEQVARQRDATEVQVFQLHYAQAADHTTRIGGQDIQVPGMASLLRNIYGVRGAPTAALPGPGANFGRVQPIGGGSSNTFGNSGQRQSGGSGILGLPASWFGAGSPSERVPVSPPLPGSGNSANAPASVWPEMSQARRDAPLAVDAGSGGELASDAPVIEADPRTNGILIRDRPERMAAYGTLIQQLDNRPKLLQIDATIIEIRDGALQDLGVDWRFHSRRVDVQTGDGRGGQLGYDGSLSGAAAAGAAAPLGGTLTAVLGDAGRYLMTRVSALEQTNKAKIVSTPQVATLDNVEAVMDHKQQAFVRVSGYASADLYNLSAGVSLRVLPSVVPGSPNGQMRLDVRIEDGQLGANTVDGIPVITSSEITTQAFVNEGQSLLIAGYASDTDQTDLNNVPGLSRIPLVGNLFKHRQQSGSRLQRLFLLTPHIVSP |
| HrcJ | - | Outer membrane ring protein | MRTLRYLVVLLLALLLSGCDQQLYSGLTENDANDMAVLLTAGVDAEKLTPDDGKTWAVNAPHDQVAYALNVLRTHGMPHERHANLGEMFKKDGLISTPTEERVRFIYGVSQQLSQTLSNIDGVIAADVQIVLPNNDPLSASVKPSSAAVFIKFRVGSDLTSLVPSIKTLVMHSVEGLTYENVSVTLVPGGAESDAQFAASAPPRAWAWPWLVGCALALCVAVAAAALYWWPSANARRWGGWQRLRALSRKHAG |
| HrcN | - | ATPase | MLAEMPLLQTTLERELAALAFGRRYGKVVEVIGTMLKVAGVQVSLGEVCELRQRDGTLLQRAEVVGFSRTLALLAPFGELVGLSRQTRVIGLGRPLAVPVGSALLGRVLDGLGEPADGQGPLAGDDWVQIQAQAPDPMRRRLIEQPLPTGVRIVDGLMTLGEGQRMGIFAAAGVGKSTLIGMFARGTQCDVNVIVLIGERGREVREFIEMILGPDGLARSVVVCATSDRSSIERAKAAYVGTAIAEYFRDRGMRVLLMMDSLTRFARAQREIGLAAGEPPTRRGFPPSVFAELPRLLERAGMGETGSITAFYTVLAEDDTGSDPIAEEVRGILDGHLILSREIAARNQYPAIDVLGSLSRVMSQIVSAEQRQYAGQLRRLLAKHNEVETLLQVGEYRHGSDAVADEAIARIDAIRDFLSQPTDQLSDYDTILEQLAGVIDDA |
| HrcQ | - | - | MLSTEPSLTSPARDLSQALTHVPAVRAQLGRVLCDPRAAQRCGYTAQRRGIRAADAARLQLQFDSGSLELRIAARDGLALLLNEADDALRVAIAGVLLSDDLRALEPLGLGAAEVVAFERCADAVDRLDIGITLGGIDAIAETASPLLLAALQTASAALAQPSPLPAWLSALRVTTRLRIGQRTATAALLQSLRPGDVLLHALATAPVRSGELLWGIPGGAVLRAPVRLTLQQMILETAPTMQHDMPASDSSSSATDVAALELPVQLEVDQLALSLSVLSGLQPGQILELSVPVDQADIRLVVYGQTIGIGRLLAVGEHLGVQILSMSETAHADA |
| HrcR | - | Export apparatus subunit | MQMPDVGSLLLVVIMLGLLPFAAMVVTSYTKIVVVLGLLRNAIGVQQVPPNMVLNGVALLVSCFVMAPVGMEAFKAAQNYSPGADNSRVVVLLDACREPFRQFLLKHTREREKAFFIRSAQQIWPKDKADTLKPDDLLVLAPAFTLSELTEAFRIGFLLYLVFIVIDLVVANALMAMGLSQVTPTNVAIPFKLLLFVALDGWSMLIHGLVLSYR |
| HrcS | - | - | MDHDDLVRFTSEALLLCLKVSLPVVGVAAVAGLLIAFIQAVMSLQDASISFALKLVVVVAAIAVTAPWGASAIMQFGQALMQAAFP |
| HrcT | HrpB8 | Export apparatus subunit | MSDTATALLALSSQGVSLLTLLALCGVRVFVLFFVLPATAQDSLPGMTRNGVIYVLSSFIAYGQPADALARIEAAGLVGLVFKEAFIGLLIGFAASTVFWVAESVGLLIDDVSGYNNVQMINPLSGEQSTPVSTVLMQLAIVSFYALGGMLMLLGALFESFRWWPLSQLMPDMGAIGESFVIQQTDGMMAAIVKLSAPVMLVLVLVDLAIGFVARAADKLDPSNLSQPIRGVLALLLLALLTSVFIAQSGDALGFLHFQQQLHDAANASAKGGASH |
| HrcU | - | Export apparatus subunit | MSDEKTEKPTEKKLQDARRDGEVPISPDVTAAAVLLAALLVMKLAGSYFVEHLRALMSIGFDFTTNTRDATALHRALGRIGIQGVLLTLPFVTACLAAGLIGTFVQTGLNASLKPVTPKFDSLNPVNGVKKLFSLRSLINLLKLGIKAAVIGVVLWYGLRALMPTIIGLAYQPPADIAQIGWRALGILCALAVLVFVLVGAADWSVQHWLFIRDKRMSKDEQKREHKESEGDPEVKGKRKEFAKELVFGDPRERVAKAKVMVVNPTHYAVALAYEPDGFGLPQVVAKGVDEGALELRAYAHNQGIPIVANPPLARALHEVELGEAVPESLFETVAVVLRWVDELGRDNDEGSGPLPC |
| HrcV | FHIPEP | - | MRVTRYFAYTGEVAIAALVVAVIGLMILPLPTPLIDTLLGINITLSVVLLMVTMYVPDSISLSSFPSLLLFTTLLRLSLNIASTKSILLHAEAGHIIESFGELVVGGNLVVGLVVFLIITTVQFIVIAKGSERVAEVGARFTLDAMPGKQMSIDADLRGGNLTADEARRKRARLAIESQLHGGMDGAMKFVKGDAIAGLVITMVNILAGIVVGVTYHGMSAGEAANRFAILSVGDAMVSQIASLLISVAAGVMITRVANENETKISSLGLDIGRQLTSNARALMAASVLLACFAFVPGFPALLFLLLAAAVGAGGYTIWRKQRDTSGSDQPALPSTSRKGAKGDAPHIRKSAPDFASPLSMRLSPQLAARLDPALLDQAIESERRQLVELLGLPFPGIAIWQSESLQGLQYEVLIHDVPETRSALSDTADMQKALAQQAIAPLHARAHLFVGIQETQWMLEQVGADYPGLVAEVNKAMPAQRIADVLRRLLEERIPVRNIKSILESLVVWGPKEKDLLMLTEYVRCDLGRYLAHTATAGTGQLPAVMLDHAVEQLIRQSIRATPAGNFLALPPEQANQLVEQVERIVEDQARHPLAVVASMDVRRYVRRMIEARLNWLEVYSFQELGAEVQLQPIGRVVA |
| HrpB1 | - | - | MEKIECPGSVVSGLIELITVGLTHEKIEEAAAVLAAVRVLRPELKALDTFDAWIAIKRGSYLEGARLLRELEADAGSEPLCKALYACCLFAVGDPSWHGIAEGLIEEDADADAVGLVKALSGRYTPAPAPLEAAAESAAPMDVPNAQYLRA |
| HrpD5 | HPr kinase, HrpQ | Kinase | MTMQLRVLTGIHAGARLDLQPGSYTLGADPQAEIRIEDWPDCSLIIEVDADGQVCYRSEALPTTAFVALHPVRFGPLVLCMGDAAADWPDDVALLEQLLSPAATPAAPSPRRSRRTALRAVVGAMLALAAAALLPSLLPAFLSDAAPPRSQDNQLNQVRFVLKRLGLREARVEQVGSRVRVEGLVTSSADAARLRAQLHRDQHAVTVDVVVVDEVLATLRDTLADRDLSVRYDGQGVFSIAGSSDNAERATRRIADLRSDLGPEIRTLHVEITQQDPSVKPPANYDAALLADGLHYVETPDGTKHLTSLPQQAAP |
| HrpF | - | Serine kinase | MSLNTLSTGSTAGLFLPLTDDASSPGLLGSDSAMNDSDLLLAMDNLFLQQIYRLIAATYGNTSLNGPGSGIPGLDTPSADDLQASQPIEKRTSWPTLSAPFNVKDIKGSRLPPAVDGSSVTWEGGTLTPSELQIVSTLNQHKDKTPLEFAKLDDKINDPSTPPDLKSALQGLQKDPRLFFAIGSQGDGKCGGKIKAGDLWDFADHHQQVTALGGKNAEFNPKNIKGATPPPAAEGSSVTWDGGTLTQSQLEIVSTLNQHRDMMPIEFAKLDEKINDPATPPDLKKALQGLQQDPGLFFAMASQGHGKHHHDDQGKCNGKLIADNLYDFADRHPQVTAQGGKNATYNPEKMKGRDLPPPVDGSSVTWDGGTLTQNELEIVATLNRHKDKCPVKWTDLDAKSKDPAIPPDLQKAFADLQQDPALFHAIGAQGSKGSCDGKFTEKDLTRFSVPEKHAQIAQYAEQQAKGYTQNYVASDSPDKTEPTVMTESDAMRELYRYSDYLPKDLNQDAFKQLVEGDSTTKKSPPQVIAAAQYFREHPDQWKALAGDKESMSTADFLQKSTSEMHLTAPELKTLDTINSHQEAFFGDGKEVTRDKLDTIVKDDKADPAVRDAAKQLLGDPLLFGLLNNAITGYKKPHSFFGGGHVVDSGKISNKDFQQFYEHMTAVNKTLDTPPTHAATSPEQKKAVADMLMGKADQPEIKRKKHDVGTFSKGLHEFLKWDSKILDGISVALSAMNGIPLIGEVADAAALAFESEAQAAQVIDTALQGGNLSLAWKLAGINMAGAVVGAVGGPTARLAAKGAAKGVAEGAAKGATQGTTKGAAKGGGKGVAEREKPLDIAKGYIIGTSINRPTEMLKTPVLAGLHYEEVRLDKEKKKGEIRKNLEAAGGVPLGKQFIPKAIADNFEADTKENLRHVRGRRK |
